# Diversity and carbon storage across the tropical forest biome

**DOI:** 10.1038/srep39102

**Published:** 2017-01-17

**Authors:** Martin J. P. Sullivan, Joey Talbot, Simon L. Lewis, Oliver L. Phillips, Lan Qie, Serge K. Begne, Jerôme Chave, Aida Cuni-Sanchez, Wannes Hubau, Gabriela Lopez-Gonzalez, Lera Miles, Abel Monteagudo-Mendoza, Bonaventure Sonké, Terry Sunderland, Hans ter Steege, Lee J. T. White, Kofi Affum-Baffoe, Shin-ichiro Aiba, Everton Cristo de Almeida, Edmar Almeida de Oliveira, Patricia Alvarez-Loayza, Esteban Álvarez Dávila, Ana Andrade, Luiz E. O. C. Aragão, Peter Ashton, Gerardo A. Aymard C., Timothy R. Baker, Michael Balinga, Lindsay F. Banin, Christopher Baraloto, Jean-Francois Bastin, Nicholas Berry, Jan Bogaert, Damien Bonal, Frans Bongers, Roel Brienen, José Luís C. Camargo, Carlos Cerón, Victor Chama Moscoso, Eric Chezeaux, Connie J. Clark, Álvaro Cogollo Pacheco, James A. Comiskey, Fernando Cornejo Valverde, Eurídice N. Honorio Coronado, Greta Dargie, Stuart J. Davies, Charles De Canniere, Marie Noel Djuikouo K., Jean-Louis Doucet, Terry L. Erwin, Javier Silva Espejo, Corneille E. N. Ewango, Sophie Fauset, Ted R. Feldpausch, Rafael Herrera, Martin Gilpin, Emanuel Gloor, Jefferson S. Hall, David J. Harris, Terese B. Hart, Kuswata Kartawinata, Lip Khoon Kho, Kanehiro Kitayama, Susan G. W. Laurance, William F. Laurance, Miguel E. Leal, Thomas Lovejoy, Jon C. Lovett, Faustin Mpanya Lukasu, Jean-Remy Makana, Yadvinder Malhi, Leandro Maracahipes, Beatriz S. Marimon, Ben Hur Marimon Junior, Andrew R. Marshall, Paulo S. Morandi, John Tshibamba Mukendi, Jaques Mukinzi, Reuben Nilus, Percy Núñez Vargas, Nadir C. Pallqui Camacho, Guido Pardo, Marielos Peña-Claros, Pascal Pétronelli, Georgia C. Pickavance, Axel Dalberg Poulsen, John R. Poulsen, Richard B. Primack, Hari Priyadi, Carlos A. Quesada, Jan Reitsma, Maxime Réjou-Méchain, Zorayda Restrepo, Ervan Rutishauser, Kamariah Abu Salim, Rafael P. Salomão, Ismayadi Samsoedin, Douglas Sheil, Rodrigo Sierra, Marcos Silveira, J. W. Ferry Slik, Lisa Steel, Hermann Taedoumg, Sylvester Tan, John W. Terborgh, Sean C. Thomas, Marisol Toledo, Peter M. Umunay, Luis Valenzuela Gamarra, Ima Célia Guimarães Vieira, Vincent A. Vos, Ophelia Wang, Simon Willcock, Lise Zemagho

**Affiliations:** 1 School of Geography, University of Leeds, Leeds, UK; 2 Department of Geography, University College London, London, UK; 3 Plant Systematic and Ecology Laboratory, University of Yaounde I, Cameroon; 4 Université Paul Sabatier CNRS, Toulouse, France; 5 United Nations Environment Programme World Conservation Monitoring Centre, Cambridge, UK; 6 Jardín Botánico de Missouri, Oxapampa, Perú; 7 Universidad Nacional de San Antonio Abad del Cusco, Cusco, Perú; 8 CIFOR, Bogor, Indonesia; 9 College of Marine and Environmental Sciences, James Cook University, Cairns, Australia; 10 Naturalis Biodiversity Center, Leiden, Netherlands; 11 Ecology and Biodiversity Group, Utrecht University, Utrecht, Netherlands; 12 Agence Nationale des Parcs Nationaux, Libreville, Gabon; 13 Institut de Recherche en Ecologie Tropicale, Libreville, Gabon; 14 School of Natural Sciences, University of Stirling, Stirling, UK; 15 Mensuration Unit, Forestry Commission of Ghana, Kumasi, Ghana; 16 Graduate School of Science and Engineering, Kagoshima University, Japan; 17 Instituto de Biodiversidade e Floresta, Universidade Federal do Oeste do Pará, Santarém, Brazil; 18 Universidade do Estado de Mato Grosso, Nova Xavantina, Brazil; 19 Center for Tropical Conservation, Duke University, Durham, NC, USA; 20 Red para la Mitigación y Adaptación al Cambio Climático de la UNAD, Bogota, Colombia; 21 Instituto Nacional de Pesquisas da Amazônia, Manaus, Brazil; 22 Geography, College of Life and Environmental Sciences, University of Exeter, Exeter, UK; 23 Department of Organismic and Evolutionary Biology, Harvard University, Cambridge, MA, USA; 24 Programa de Ciencias del Agro y el Mar, Herbario Universitario, Barinas, Venezuela; 25 CIFOR, Conakry, Guinea; 26 Centre for Ecology and Hydrology, Penicuik, UK; 27 International Center for Tropical Botany, Department of Biological Sciences, Florida International University, Miami, FL, USA; 28 UMR AMAP, IRD, Montpellier, France; 29 UPR BSEF, CIRAD, Montpellier, France; 30 The University of Edinburgh, School of GeoSciences, Edinburgh, UK; 31 Biodiversity and Landscape Unit, Gembloux Agro-Bio Tech, Université de Liège, Gembloux, Belgium; 32 INRA, UMR EEF, Champenoux, France; 33 Forest Ecology and Forest Management group, Wageningen University, Wageningen, The Netherlands; 34 Instituto Nacional de Pesquisas da Amazônia, Projeto Dinâmica Biológica de Fragmentos Florestais, Manaus, Brazil; 35 Herbario Alfredo Paredes, Universidad Central del Ecuador, Quito, Ecuador; 36 Rougier-Gabon, Libreville, Gabon; 37 Nicholas School of the Environment, Duke University, Durham, NC, USA; 38 Jardín Botánico Joaquín Antonio Uribe, Medellín, Colombia; 39 Inventory & Monitoring Program, National Park Service, Fredericksburg, VA, USA; 40 Andes to Amazon Biodiversity Program, Puerto Maldonado, Perú; 41 Instituto de Investigaciones de la Amazonia Perúana, Iquitos, Perú; 42 Smithsonian Tropical Research Institute, Washington, DC, USA; 43 Landscape Ecology and Vegetal Production Systems Unit, Universite Libre de Bruxelles, Brussels, Belgium; 44 Department of Botany & Plant Physiology, Faculty of Science, University of Buea, Buea, Cameroon; 45 Forest Ressources Management, Gembloux Agro-Bio Tech, University of Liege, Belgium; 46 Smithsonian Institution, Washington, DC, USA; 47 Wildlife Conservation Society-DR Congo, Kinshasa I, Democratic Republic of Congo; 48 Centre de Formation et de Recherche en Conservation Forestiere (CEFRECOF), Democratic Republic of Congo; 49 Institute of Biology, UNICAMP, Campinas, Brazil; 50 Centro de Ecologia, Instituto Venezolano de Investigaciones Cientificas, Caracas, Venezuela; 51 Institut für Geographie und Regionalforschung, Geoökologie, University of Vienna, Vienna, Austria; 52 Smithsonian Tropical Research Institute, Panamá, Republic of Panama; 53 Royal Botanic Garden Edinburgh, Edinburgh, UK; 54 Lukuru Wildlife Research Foundation, Kinshasa, Gombe, Democratic Republic of Congo; 55 Division of Vertebrate Zoology, Yale Peabody Museum of Natural History, New Haven, CT, USA; 56 Herbarium Bogoriense, Indonesian Institute of Sciences, Bogor, Indonesia; 57 Integrative Research Center, The Field Museum, Chicago, IL, USA; 58 Tropical Peat Research Institute, Biological Research Division, Malaysian Palm Oil Board, Selangor, Malaysia; 59 Kyoto University, Kyoto, Japan; 60 Centre for Tropical Environmental and Sustainability Sciences and College of Science and Engineering, James Cook University, Cairns, Australia; 61 Wildlife Conservation Society, Kampala, Uganda; 62 Department of Environmental Science and Policy, George Mason University, Fairfax, VA, USA; 63 Faculté des Sciences Agronomiques, Université de Kisangani, Kisangani, Democratic Republic of Congo; 64 School of Geography and the Environment, University of Oxford, Oxford, UK; 65 Universidade Federal de Goiás, Goiânia, Brazil; 66 Flamingo Land Ltd, Kirby Misperton, UK; 67 CIRCLE, Environment Department, University of York, York, UK; 68 Salonga National Park, Kinshasa I, DR Congo; 69 Sabah Forestry Department, Sabah, Malaysia; 70 Universidad Autónoma del Beni, Riberalta, Bolivia; 71 Instituto Boliviano de Investigación Forestal, Santa Cruz de la Sierra, Bolivia; 72 CIRAD, UMR Ecologie des Forêts de Guyane, Sinamary, French Guiana, France; 73 Natural History Museum, University of Oslo, Oslo, Norway; 74 Department of Biology, Boston University, Boston, MA, USA; 75 CIFOR, Bogor, Indonesia; 76 Southern Swedish Forest Research Center, Swedish University of Agricultural Sciences, Alnarp, Sweden; 77 Bureau Waardenburg, The Netherlands; 78 Fundación Con Vida, Medellín, Colombia; 79 Carboforexpert, Geneva, Switzerland; 80 Environmental and Life Sciences, Faculty of Science, Universiti Brunei Darussalam, Brunei, Darussalam; 81 Museu Paraense Emilio Goeldi, Belém, Brazil; 82 FORDA, The Ministry of Forestry and Environment, Bogor, Indonesia; 83 Norwegian University of Life Sciences, Aas, Norway; 84 GeoIS, Quito, Ecuador; 85 Museu Universitário, Universidade Federal do Acre, Brazil; 86 World Wildlife Fund, Washington, DC, USA; 87 CTFS-AA Asia Program, Harvard University, Cambridge, MA, USA; 88 Faculty of Forestry, University of Toronto, Toronto, Canada; 89 Yale School of Forestry & Environmental Studies, New Haven, CT, USA; 90 Centro de Investigación y Promoción del Campesinado - Regional Norte Amazónico, Riberalta, Bolivia; 91 School of Earth Sciences and Environmental Sustainability, Northern Arizona University, Flagstaff AZ, USA; 92 Biological Sciences, University of Southampton, Southampton, UK; 93 School of Environment, Natural Resources and Geography, Bangor University, Bangor, UK

## Abstract

Tropical forests are global centres of biodiversity and carbon storage. Many tropical countries aspire to protect forest to fulfil biodiversity and climate mitigation policy targets, but the conservation strategies needed to achieve these two functions depend critically on the tropical forest tree diversity-carbon storage relationship. Assessing this relationship is challenging due to the scarcity of inventories where carbon stocks in aboveground biomass and species identifications have been simultaneously and robustly quantified. Here, we compile a unique pan-tropical dataset of 360 plots located in structurally intact old-growth closed-canopy forest, surveyed using standardised methods, allowing a multi-scale evaluation of diversity-carbon relationships in tropical forests. Diversity-carbon relationships among all plots at 1 ha scale across the tropics are absent, and within continents are either weak (Asia) or absent (Amazonia, Africa). A weak positive relationship is detectable within 1 ha plots, indicating that diversity effects in tropical forests may be scale dependent. The absence of clear diversity-carbon relationships at scales relevant to conservation planning means that carbon-centred conservation strategies will inevitably miss many high diversity ecosystems. As tropical forests can have any combination of tree diversity and carbon stocks both require explicit consideration when optimising policies to manage tropical carbon and biodiversity.

Biodiversity is threatened by the loss of natural habitats and climate change^1^,^2^,^3^. Tropical forests are under particular pressure, whilst also being the most diverse biomes on the planet^4^. By legally protecting areas, tropical countries can safeguard ecosystems with high biodiversity value^5^, and so address their policy targets to reduce biodiversity loss^6^. Likewise, carbon losses from the conversion of forest to other land-uses represent major emission sources for many tropical countries^7^, and so incentives such as the UN REDD+ policy framework have emerged to help safeguard areas with high carbon stocks^8^. Yet the potential for protection of carbon-rich areas to directly benefit biodiversity, and vice versa, depends critically on the relationship between biomass carbon and tree diversity, at relevant scales. A positive relationship would indicate potential synergies while a negative relationship would indicate difficult trade-offs between biodiversity and carbon conservation^9^. In the absence of any relationship, optimal solutions for protected area placement need to carefully and separately consider the distribution of carbon stocks and the distribution of biodiversity^10^. Understanding these distributions and potential carbon-biodiversity trade-offs is important, as protecting some forest can divert threats onto other unprotected areas^11^.

The expected form of diversity-carbon relationships in tropical forests and the strength and scale-dependence of any underlying mechanisms are uncertain. Numerous experimental studies have demonstrated that plant diversity promotes biomass production, with niche partitioning and positive species interactions allowing diverse communities to exploit available resources more efficiently^12^,^13^. Diversity can also increase productivity through selection effects, where communities that contain a larger sample of the species pool are more likely to contain high functioning species that contribute strongly to ecosystem productivity^14^. Positive diversity-productivity relationships have been found in low diversity mid-latitude forests^15^,^16^,^17^, potentially due to increased canopy packing through complimentary canopy architecture in higher diversity forests^18^. Yet, it is unclear how significant such mechanisms are in diverse tropical forests, as experimental and theoretical work indicates that the positive effect of diversity may saturate at high species richness^12^,^19^. Furthermore, additional traits associated with high-productivity species could conceivably lead to a positive diversity-biomass mortality relationship, as highly productive stands tend to be composed of trees with shorter biomass residence times^20^. Overall, this alongside high-productivity stands consisting of smaller, lighter-wooded trees^21^, may lead to a negative diversity-biomass carbon storage relationship.

Previous studies investigating the tree diversity-carbon stock relationship in tropical forests have reported a positive relationship at fine spatial scales^22^,^23^. However, the form of the relationship at the stand-level (i.e. among 1 ha plots) is less clear (Table 1), as some studies report a continued positive diversity-carbon relationship among sampling locations^23^,^24^,^25^, while one other did not detect a relationship among 1 ha subplots within 25 larger plots^22^. Thus, while there is some evidence that higher tree diversity promotes higher carbon stocks per unit area in diverse tropical forests^22^,^23^,^24^, it is unknown whether any positive effect is strong enough for carbon and diversity to co-vary at scales relevant to conservation planning.

Here we analyse a unique dataset of 360 inventory plots across the three major tropical forest blocs in the Americas, Africa, and the Sundaland biogeographic region in Southeast Asia (subsequently referred to as Asia). Importantly, this dataset greatly improves sampling of the two most extensive contiguous areas of tropical forest in the world, centred on the Amazon and Congo Basins (Table 1). Each plot was surveyed by standardised methods and is of uniform size, allowing robust quantification of co-located aboveground live carbon and tree diversity estimates. We analyse this standardised, multi-continental dataset at three spatial scales. Firstly, we explore forest carbon and diversity patterns within South America, Africa and Asia, in order to characterise among-continent variations in tree alpha diversity, beta diversity, and carbon stocks. Secondly, we assess diversity-carbon relationships across each of the continents, initially by looking at the bivariate association of tree diversity metrics and carbon stocks per unit area, and then re-examining the relationships after controlling for potentially confounding environmental variation and residual spatial autocorrelation. Finally, we investigate fine-scale relationships between tree diversity and carbon within 0.04 ha subsections of 1 ha plots, where environmental differences that may obscure a positive diversity effect on carbon are accounted for. This approach allows us to (1) examine basic patterns of diversity and carbon across the biome, (2) test if more diverse tropical forests are also in fact more carbon dense, and (3) explore whether relationships between diversity and carbon-storage, after accounting for the effect of potentially confounding variables, are consistent with tree diversity having a positive effect on carbon in tropical forests. We conduct additional analyses to assess support for the operation of selection effects and niche complementarity at different spatial scales. We focus on carbon in aboveground live biomass derived using allometric relationships, and diversity metrics relating to taxon richness. We also repeat analyses using alternative diversity metrics that consider species abundance and functional diversity for which results and inferences are similar (see Supplementary Information).

## Results

### Pantropical forest carbon and diversity

**Figure 1:**
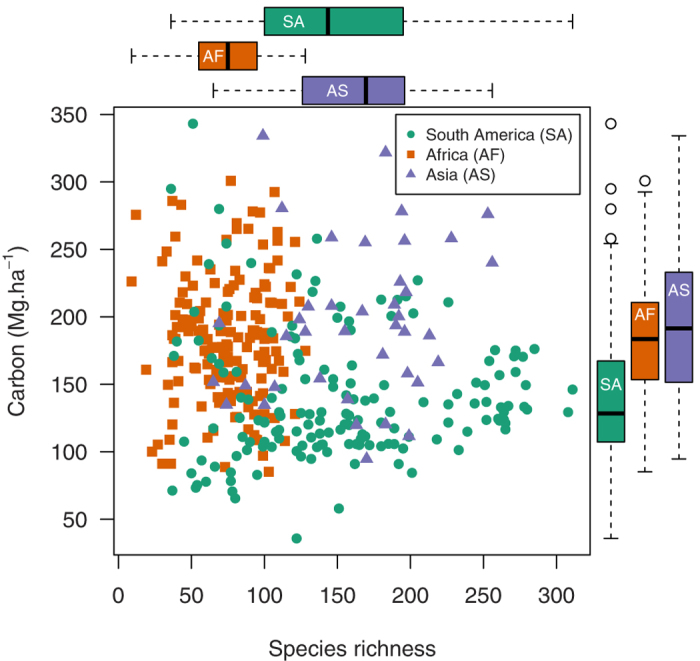
No relationship across the tropical forest biome between carbon stocks per unit area and tree species richness. Green circles = plots in South America (n = 158), orange squares = Africa (n = 162) and purple triangles = Asia (n = 40). Boxplots show variation in species richness and biomass carbon stocks in each continent. Both carbon and species richness differed significantly between continents (Table 2), but no significant correlation exists between carbon and species richness, neither within each continent (τ ≤ 0.132, *P* ≥ 0.12), nor across all three (linear regression weighted by sampling density in each continent, β < −0.001, *t* = 0.843, *P* = 0.4, weights = 1.2 for South America, 0.6 for Africa and 1.8 for Asia). Results for other diversity metrics are similar (Supplementary Fig. S13).

Our standardised methods of inventory reveal great variation in both aboveground live carbon stocks and tree diversity within continents and across the humid tropical forest biome. While it is possible to find almost any combination of both parameters (Fig. 1), the plots reveal large differences in carbon and diversity amongst the three continents (Table 2). African tropical forests are characterised by high carbon storage per unit area and consistently low alpha-diversity (even the most species-rich African plot had fewer species than the median species richness recorded in South America and Asia). By contrast, in South American plots carbon storage per unit area was lower than in African forests (Fig. 1). Nevertheless both diversity and carbon vary greatly within South America, as reflects previously reported gradients in species richness^26^ and biomass^27^,^28^, with some stands in the Guiana Shield region containing carbon stocks comparable to forests in the paleotropics (Fig. 1). Asian forests differ again, having on average both high carbon storage per unit area and high tree diversity. These differences in diversity amongst continents remain when diversity metrics are standardised per 300 stems (Table 2), and when the analysis was repeated only including plots with >90% of stems identified to species level (Supplementary Table 3), thus are robust to differing stem numbers (lower in Africa, negative binomial GLM χ^2^ = 188.6, *P* < 0.001), and are unaffected by our levels of tree identification (not different amongst continents, Kruskal-Wallis test *H* = 2.1, *P* = 0.335). This pantropical assessment of forest carbon stocks and diversity is consistent with previous reports from individual continents, indicating high biomass in forests in Africa^29^ and Borneo^30^,^31^, high diversity in central and western Amazonia^32^ and low diversity in Africa^33^,^34^. Our analysis demonstrates that forests across the Sundaland region of Southeast Asia are not only amongst the most diverse in the tropics, as noted elsewhere^33^, but also amongst the most carbon-dense.

**Figure 2:**
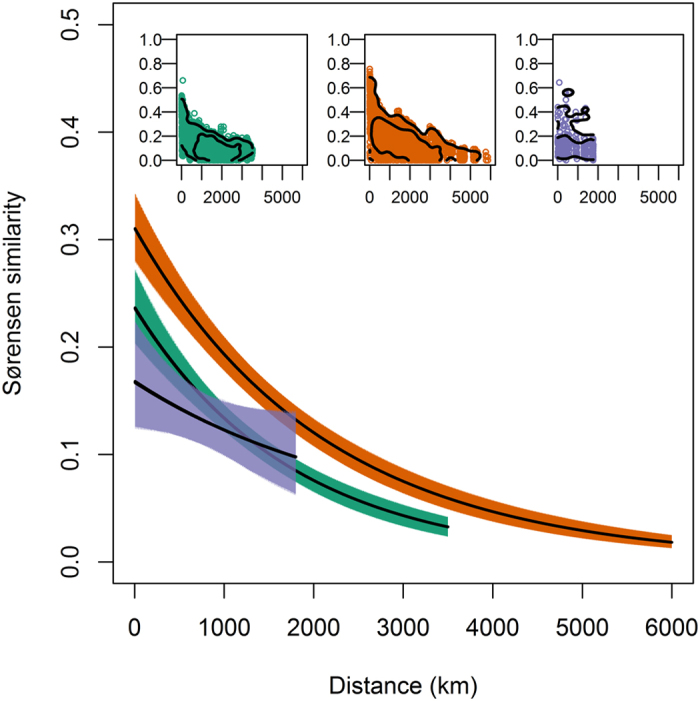
Decay in similarity (Sørensen index) of tree communities with distance in South America (green), Africa (orange) and Asia (purple). Solid lines show fitted relationships of the form ln(similarity) = α + β × distance + ε. Estimated α and β parameters for each continent are given in Supplementary Fig. S12, ε denotes binomial errors. Differences in the α parameter indicate differences in the similarity of neighbouring stands, while differences in the β parameter indicate differences in the distance decay of tree community similarity. Filled polygons show 95% confidence intervals derived from 10000 bootstrap resamples. Data underlying these relationships are shown in insets, with contours (0.05 and 0.25 quantiles) overlain to show the density of points following kernel smoothing.

Beta-diversity also showed contrasting patterns amongst continents. Tree communities in neighbouring forests were least similar in Asia and most similar in Africa, where diversity rapidly saturates over geographic distance and plots (Fig. 2, Supplementary Fig. 11). However while similarity in species composition decayed most strongly with distance in South America, there was weaker distance decay in Asia (Fig. 2, Supplementary Fig. 12). As a result, while adjacent stands differ most in Asia, at distances >1,000 km plots in Asia are no more dissimilar than equidistant plot pairs in South America. Differences in beta diversity could have been driven by differences in gamma diversity^35^. However, local tree communities remained more similar in Africa than other continents when null models were used to account for variation in gamma diversity (Supplementary Fig. 13). Gamma diversity was comparable in South America and Asia^33^, so was also unlikely to drive differences in the distance decay of tree community similarity in those continents.

### Large-scale diversity-carbon relationships

Notably, aboveground carbon stocks in live biomass per unit area was unrelated to tree species richness amongst 1 ha plots, whether analysed within continents or when combining all data in a pan-tropical analysis (Fig. 1, Table 3). Correlations with other diversity metrics varied in sign but were also non-significant (Table 3, Supplementary Fig. 14). Thus, in tropical forests high values of diversity and biomass carbon are associated neither at the biome nor the continental scale; instead they vary independently. We note that while in both South America and Africa there is sufficient statistical power to detect even small effects of diversity had they existed, in Asia power was only sufficient to detect relatively large effect sizes (Table 2).

**Figure 3:**
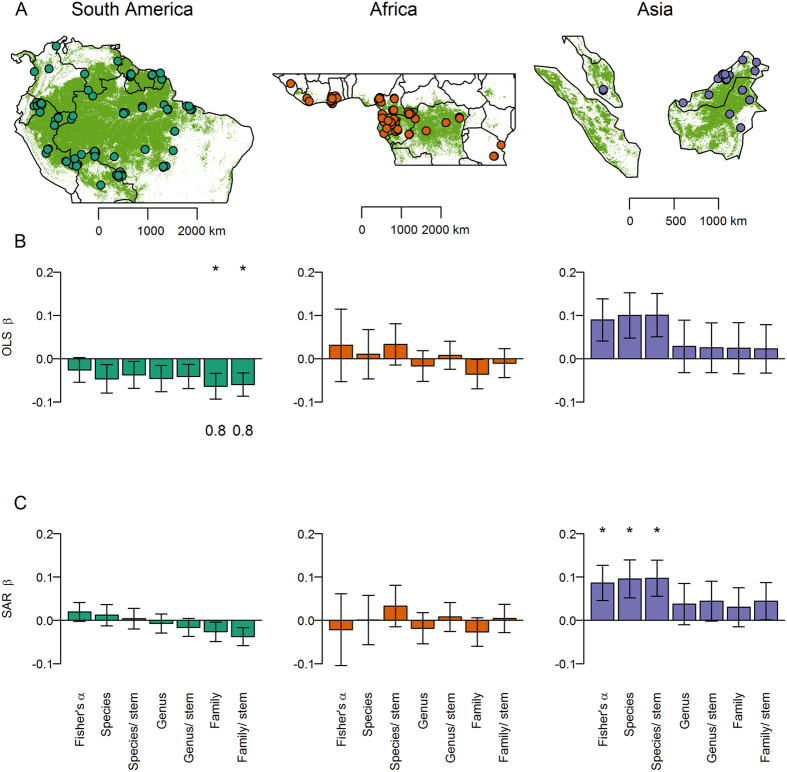
Stand-level effect of diversity on carbon stocks per unit area. (**A**) Location of clusters of forest inventory plots in South America (n = 158 plots), Africa (n = 162 plots) and Asia (n = 40 plots) (some cluster centroids are not visible due to over plotting). (**B** & **C**) Diversity metric coefficients in multiple regressions relating carbon to diversity, climate and soil. Results have been presented for (**B**) non-spatial (OLS) and (**C**) simultaneous autoregressive error (SAR) models. Bars show model-averaged parameter estimates, with error bars showing standard errors. Asterisks denote variables that were significant in the average model (*P* < 0.05), with the summed AIC_C_ weights of models in which a variable appears shown beneath bars (where >0.75). Taxa/stem denotes richness estimates per 300 stems. SAR models indicate that increasing species richness by 1 SD (from 86 to 151 species.ha^−1^) increased carbon by 1.5 Mg.ha^−1^ in South America, 0.2 Mg.ha^−1^ in Africa and 15.8 Mg.ha^−1^ in Asia (note only the relationship in Asia was statistically significant). Green shading in (**A**) shows the extent of broadleaved evergreen and fresh water regularly flooded forest classes from^52^. Model coefficients are given in Supplementary Table 5. Maps were created in R version 3.02 (http://www.R-project.org/)^53^ using base maps from maps package version 2.3–9 (http://CRAN.R-project.org/package=maps)^54^.

Since confounding environmental variables might obscure any underlying effect of tree diversity on carbon stocks, we next applied multiple regression including climate and edaphic variables as covariates to statistically control for environmental variation that might otherwise obscure the effect of diversity. In ordinary least squares multiple regression models, there was a consistent negative relationship between diversity and carbon in South America, and no significant relationship in Africa and Asia (Fig. 3b). When the analysis was repeated using simultaneous autoregressive error models to account for spatial autocorrelation, diversity was not supported as a predictor in South America or Africa (Fig. 3c). In Asia, while there were significant positive relationships between carbon and both Fisher’s α and species richness (Fig. 3c), environmental variables were more important predictors of carbon stocks based on their occurrence in low AIC_C_ models (Supplementary Table 5) and other diversity metrics were not supported as predictors of carbon stocks (Fig. 3c). Thus, overall no consistent pan-tropically applicable relationship between diversity and carbon stocks was observed. Instead, carbon stocks per unit area was influenced by climate and soil (Supplementary Fig. 15, Supplementary Table 5). In South America and Africa annual cumulative water deficit was the strongest environmental predictor of carbon stocks, as indicated by high ∑ AIC_C_ weights (≥0.98), and in South America a positive effect of soil fertility was also evident (Supplementary Fig. 15, Supplementary Table 5). In Asia, where no plots experienced cumulative water deficit, carbon stocks per unit area increased with mean annual precipitation (∑ AIC_C_ weights = 1) and declined with mean annual temperature (∑ AIC_C_ weights = 0.65).

Carbon stocks per unit area was also related to structural attributes, increasing with basal area and basal area-weighted mean wood density, but not with stem density (Supplementary Fig. 16). While consistent with previous studies^23^, this is hardly surprising as both wood density and basal area are constituents of biomass estimates. Critically, these two structural attributes of carbon stocks per unit area were themselves largely unrelated to species richness (Supplementary Fig. 16), indicating that diversity is not a correlate of the key structural factors that lead to high biomass in some tropical forest stands. Stem size inequality, which has been posited as a mechanism linking diversity and carbon in boreal forests^36^, was positively related to carbon but unrelated to species richness (Supplementary Fig. 17). Inclusion of mean wood density (a proxy for stem turnover) in multiple regression models did not affect diversity-carbon relationships (Supplementary Table 6), indicating that the lack of a consistent diversity-carbon relationship is unlikely to be due to variation in mortality. Finally, we also used structural equation modelling to examine the relationship between diversity and carbon while explicitly modelling the effect of climate and soil on both tree species richness and carbon stocks. In this modelling framework, there were non-significant positive relationships between species richness and carbon in Africa and Asia and a significant negative relationship in South America (Supplementary Figure 18).

### Fine-scale diversity-carbon relationships

**Figure 4:**
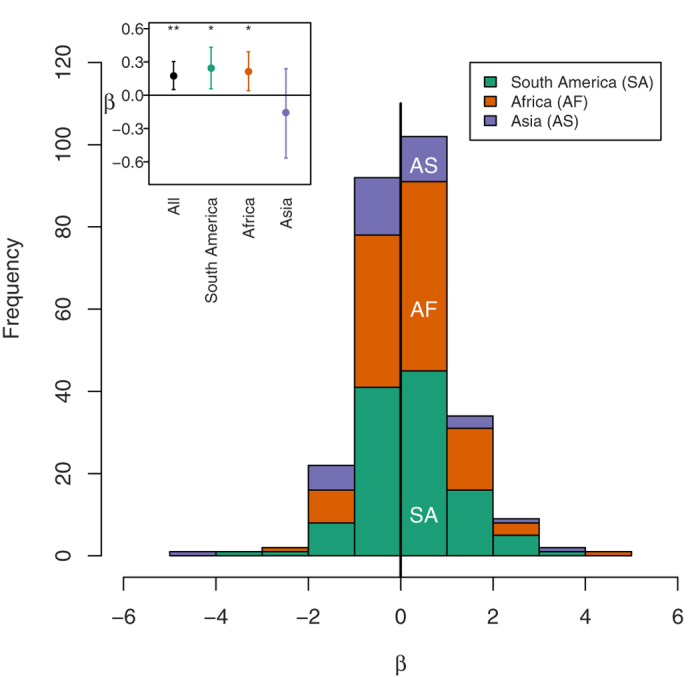
Variation in the coefficient (β) of the relationship between species richness and carbon among 0.04 ha subplots within 266 1 ha plots. Coefficients come from multiple regression models also containing the number of stems as a second-order polynomial term to allow for a saturating relationship. Coefficients from plots in South America are shown in green, Africa in orange and Asia in purple. Mean values of coefficients are shown in the inset, with error bars showing 95% confidence intervals derived from 10000 bootstrap resamples (with replacement) of the dataset, with asterisks denoting significant differences from zero (one-sample Wilcoxon test, **P < 0.01, *P < 0.05). Across all plots, doubling species richness in 0.04 ha subplots increased carbon by 6.9%. The horizontal line in the inset and bold vertical line in the main figure show where coefficients = 0. β is in units of ln(Mg.ha^−1^ carbon) per ln(tree species).

Amongst 0.04 ha subplots within each plot most environmental differences in climate and soil are implicitly accounted for. Here, relationships between species richness and carbon were on average significantly positive when considering all 266 × 1 ha plots for which we had subplot-scale data (one-sample Wilcoxon test, *P* = 0.007), and significant for plots within Africa (*n* = 111 plots, one-sample Wilcoxon test, *P* = 0.022) and South America alone (*n* = 118 plots, one-sample Wilcoxon test, *P* = 0.013, Fig. 4). Within these plots, 148 (55.6%) had a positive richness-carbon relationship and 118 (44.4%) a negative relationship (Fig. 4). Overall the richness-carbon relationship was weak but positive (β = 0.096 ± 0.048 SE). This implies that doubling species richness per 0.04 ha would increase carbon stocks by 6.9%, with similar relationships for other diversity metrics (Supplementary Table 7). This is consistent with an independent within-plot study of 25 plots which showed a 7% effect size of diversity on aboveground biomass at the 0.04 ha spatial scale, but no relationship at the 1 ha scale^22^.

### Examining support for niche complementarity and selection effects

There was a statistically significant positive relationship between a multivariate metric of functional diversity incorporating wood density and maximum diameter traits and carbon stocks at the 0.04 ha scale (linear mixed effects model, *P* < 0.001, Supplementary Figure 1), but this relationship was not significant in any continent at the 1 ha scale (linear regression models, *P* ≥ 0.139, Supplementary Figure 1). Carbon stocks increased with the community weighted means of both wood density and maximum diameter traits at both 0.04 ha (linear mixed effects models, *P* < 0.001, Supplementary Figure 4) and 1 ha scales (linear regression models, *P* ≤ 0.049, Supplementary Figure 4), indicating that carbon stocks was positively related to the functional dominance of potentially large and dense wooded species. The probability of sampling a species with large maximum size or dense wood increased through the range of species richness values typical of 0.04 ha subplots, but tended to saturate by the species richness values typical of 1 ha plots, with the exact form of this relationship depending on the threshold used to define a large or dense wooded species and whether the null model used to sample species randomly selected species from the pool available within a continent or sampled species according to their relative frequency of occurrence (Supplementary Figures 5–10). For example, the expected probability of sampling a tree species with maximum diameter ≥70 cm, as assessed using a null model randomly selecting species from the pool of species recorded in plots within each continent, increased from 0.760 to 0.878 over the interquartile range of species richness found in 0.04 ha subplots (i.e. 11 to 18 species), but was 0.999 by the lower quartile of species richness in 1 ha plots (i.e. 72 species). Likewise, there was a positive relationship between the observed occurrence of potentially large tree species and species richness in 0.04 ha subplots (binomial generalised linear mixed effects models, *P* < 0.001, Supplementary Figure 6), while at 1 ha scale this relationship was no longer evident as all but one 1 ha plot contained a potentially large species. Further details and interpretation of these analyses are given in Supplementary Discussion.

## Discussion

By analysing a large, standardised, pan-tropical dataset of inventory plots we were able to explore large-scale patterns in tropical forest above-ground carbon stocks per unit area and tree diversity, and the large-scale and fine-scale relationships between the two. Carbon and diversity both exhibit remarkable variation across the tropical forest biome. Each continent has a distinctive signature of alpha diversity, beta diversity and carbon-density, and tropics-wide it is possible to find all combinations of diversity and carbon. Yet, these two fundamental attributes of tropical forests are also found to be largely unrelated to one another among stands, whether analysed among-continents or within each one.

Our results contrast with those from an earlier examination of pan-tropical diversity-biomass relationships reporting a positive relationship with genus level diversity^24^ (Table 1). Although both studies statistically control for the effect of climate, we also restricted our analysis to lowland plots and statistically controlled for the effect of soil, which may have improved our ability to account for the effect of environmental variation when examining the effect of diversity on carbon stocks. Additionally, our results are based on an order of magnitude more extensive sampling of the biome (166 locations and 360 plots in this study, compared to 11 locations and 59 plots in ref. 24). Positive stand-scale diversity-carbon stock per unit area relationships have also been reported in the neotropics^23^ and in some Central African forests^25^, but these positive relationships were once again not evident with improved sampling across the whole domain and once spatial autocorrelation is accounted for. Our neotropical dataset differs from Ref. 23 by being concentrated in the Amazon basin rather than including Central America and the Caribbean Islands, and by excluding plots in dry forest; these differences may have reduced the effects of environmental and biogeographic variation in our data.

Our best sampled regional domains - the world’s two largest contiguous regions of tropical forest - show no within-continent diversity-carbon relationship at the 1 ha scale. In our dataset, tropical carbon remained positively but weakly related to diversity in Asia, and this was the exception among major tropical forest regions. Importantly, this lack of a consistent positive relationship between diversity and carbon is robust to analysis method, persisting whether data are analysed using simple bivariate correlations, or with multiple regressions to account for environmental drivers, or by simultaneous autoregressive models to also account for spatial autocorrelation, or when constructing structural equation models to account for environmental effects on diversity. Instead, we found that moisture availability (annual cumulative water deficit in South America and Africa, mean annual precipitation in Asia where plots did not experience cumulative water deficit) was the most important and pantropically consistent environmental driver of spatial variation in aboveground biomass carbon stocks per unit area.

Although tree diversity and carbon stocks were uncorrelated at the stand-level, they were positively correlated within forest stands, so our results are consistent with tree diversity having a positive local effect on carbon in tropical forests, supporting previous studies documenting positive fine-scale relationships^22^,^23^ (Table 1). The presence of a weakly positive (overall, South America, Africa) relationship at 0.04 ha but not at 1 ha scale (overall, South America, Africa) could indicate that the mechanisms driving the diversity-carbon relationship are scale dependent, or could be due to environmental variation acting at larger spatial scales obscuring the mechanistic effects of diversity^22^. Although our multiple regression models applied at 1 ha scale statistically control for important variation in climate, soil texture and soil chemistry, it is clearly not possible to capture all environmental variation that may influence carbon stocks, such as local disturbance history, so we cannot rule out the latter explanation. However, we conducted additional analyses (full details in Supplementary Discussion) to examine possible mechanisms underlying the diversity effects and explore their putative scale-dependency. Carbon stocks increased with the functional dominance of species with high wood density and large maximum diameter at both 0.04 ha and 1 ha scales (Supplementary Figure 4). The effect of functional dominance at 1 ha scale has been found before in tropical forests^24^,^37^, and has been interpreted to support the role of selection effects^16^,^24^. However, this analysis by itself is a test of the biomass ratio hypothesis^37^. For selection effects to operate, the probability of sampling a high functioning species should also increase with species richness. We found that the probability of sampling species with high maximum diameters or high wood density increases with species richness at diversity levels found in 0.04 ha subplots, but saturates at diversity levels below those of 1 ha plots (Supplementary Figures 5–10), indicating that selection effects, as expected, appear to be scale-dependent. Additionally, the effects of niche complementarity may also saturate, as we found a positive relationship between a multivariate functional diversity metric (incorporating wood density and maximum diameter traits) and carbon only at the 0.04 ha scale (Supplementary Figure 1). The absence of a significant relationship between tree functional diversity and carbon stocks per unit area at 1 ha scale is consistent with a previous analysis from three neotropical rainforests^37^. Although the saturating probability of sampling a high functioning species with increasing species richness and the absence of carbon – functional diversity relationships at 1 ha are consistent with both selection effects and niche complementarity being scale-dependent, they are based on correlative analysis of observational data so causal inferences need to be made cautiously. Neither do our analyses test other potentially important ecosystem impacts of diversity, such as on the resistance and resilience of biomass production to climate extremes^38^. Long-term large-scale experiments that manipulate tree diversity in tropical forests^39^ may provide additional mechanistic insights into potential positive effects of tree diversity and their potential saturation with scale.

A caveat with this and other studies using allometric equations to estimate above-ground biomass carbon is that allometric equations do not allow variation in tree architecture with forest structure. For example, Banin *et al*.^40^ found a weak negative relationship between tree height and stem density, meaning that allometric equations may overestimate carbon stocks in plots where stem density is highest. This could increase the chances of finding a spurious positive relationship between diversity and carbon, as we find a weak positive relationship between stem density and species richness (Supplementary Figure 16). This potential bias is unlikely to have impacted our results, as we still find a weak positive diversity-carbon relationship within plots and no relationship among plots when diversity metrics are standardised per *n* stems (Table 3, Supplementary Table 7). Such potential biases could be evaluated in the future if co-located LiDAR based aboveground biomass carbon estimates and ground-based tree diversity measurements are made at sufficient sites. The uncertainty in biomass carbon estimates due to using allometric equations could reduce the chance of finding diversity-carbon relationships by adding noise to the data. Whilst this highlights the need to maximise statistical power with large datasets, we note that the two largest studies investigating diversity-carbon relationships (this study by number of sampling locations across the biome, Ref. 22, by area sampled, see Table 1) converge on a similar result with independent datasets; diversity and carbon are positively related at the 0.04 ha scale but unrelated at the 1 ha scale.

### Conservation implications

Despite the absence of a stand-level diversity-carbon relationship, some forest stands certainly do combine high tree diversity and biomass (Fig. 1), indicating that high value carbon and biodiversity conservation can be simultaneously achieved, but only with confidence if both are considered^9^,^10^. We note that conservation strategies will also need to consider biodiversity of taxa other than trees, which may also be unrelated to carbon stocks^41^, the conservation value of specific species assemblages^3^, belowground carbon stores such as in tropical peat swamps^42^, and spatial variation in opportunity costs^43^. Methods to select protected areas that consider multiple metrics of conservation value (e.g. aboveground biomass carbon and aspects of biodiversity) are available^10^. Our results support the use of such an approach over carbon-dominated prioritisation incentivised under REDD+^9^. Applying this in practice is challenging as it requires knowledge of spatial variation in tree diversity, composition and carbon stocks, highlighting the importance of careful identifications to species level during forest inventories. As tropical forests can have any combination of tree diversity and carbon both will require explicit consideration when optimising policies to manage tropical carbon and biodiversity.

In sum, our large, pan-tropical analysis reveals that at small scales of less than 1 ha tree diversity is weakly positively correlated with aboveground carbon stocks, potentially due to both niche complementarity and sampling effects. Yet our results show that these processes do not translate to patterns at scales that matter practically for conservation: tree diversity and carbon vary independently among sites, both within continents and across the whole tropical forest biome. Despite the general lack of association between diversity and carbon, our analysis demonstrates that forests in Asia are not only amongst the most diverse in the tropics but also amongst the most carbon-dense. Thus at a global scale a clear synergy emerges, with forests in Asia being both highly speciose and extremely carbon-dense. Asian forests are under substantial threat, particularly from conversion to oil palm plantations and more intensive logging than elsewhere in the tropics. As a triple hotspot for biodiversity, carbon and threat, there is a compelling global case for prioritising their conservation.

## Methods

To permit comparisons among and within continents we utilised 360 forest inventory plots, surveyed using uniform standardised protocols, from three networks, RAINFOR (Amazon Forest Inventory Network; www.rainfor.org, ref. 44), AfriTRON (African Tropical Rainforest Observatory Network; www.afritron.org, ref. 29) and T-FORCES (Tropical Forests in the Changing Earth System; www.tforces.net). The plots were all within closed-canopy lowland (maximum altitude 1217 m above sea level) humid *terra firme* forest (mean annual temperature, MAT, ≥20 °C and mean annual precipitation, MAP, ≥1300 mm), all were 1 ha, except four of 0.96 ha, and none exceeded 500 m in maximum dimension. The rationale for restricting the environmental domain sampled was to minimise the environmental differences among plots and thus reduce the confounding effect of environmental variation on the diversity-carbon relationship; this approach contrasts with previous studies that have sampled along larger elevation (and thus temperature)^24^ and precipitation^23^ gradients In each plot at least 80% of stems were identified to genus and at least 60% to species (mean = 90.3% stems identified to species; 84% of plots had at least 80% stems identified to species, 63% had at least 90% of stems identified to species). All stem diameter measurements follow standard (above buttress) methods (see Supplementary Methods for full protocols). All stems ≥10 cm d.b.h. were measured. Sampling was distributed across the world’s three largest tropical humid forest blocs, with 158 plots in South America, 162 in Africa and 40 in Asia (Fig. 3). These came from 166 discrete localities (South America 80, Africa 67, Asia 19), where a ‘locality’ is defined as clusters of plots with maximum inter-plot distance of 5 km. Plot data were curated in ForestPlots.net^45^ or using equivalent offline procedures, with each plot following the same quality control and subsequent calculation protocol. Aboveground biomass (AGB) was estimated for each stem using the allometric equation AGB = 0.0673 × (ρ*D*^2^*H*)^0.976^, from^46^, where ρ is stem wood density (in g.cm^−3^) obtained from a world database^47^,^48^, D is stem diameter (in cm) at 1.3 m or above buttresses, and H is height (in m), the latter estimated using regional height-diameter Weibull equations^49^. AGB values were converted to estimates of carbon using the mean carbon fraction for tropical angiosperms, 47.1%, from^50^. Taxon richness was estimated as the sum of identified species and morphospecies plus the estimated number of unidentified taxa based on observed richness per stem ratios (details in Supplementary Methods). Richness per 300 stems was estimated using individual based rarefaction.

Differences in diversity and carbon among continents were assessed using analysis of variance. To meet model assumptions, carbon stocks per unit area was log-transformed and Fisher’s alpha square-root transformed, while taxon richness was modelled using a negative binomial error distribution to account for overdispersion. We used log-linear generalised linear models with binomial errors to model the relationship between Sørensen index (beta diversity) and geographical distance between plots in each continent, restricting this analysis to plots with >90% of stems identified to species level (227 plots). Relationships among 1 ha plots were assessed using [1] bivariate Kendall’s τ correlations and [2] multiple regressions of carbon as a function of diversity, climate (cumulative water deficit, MAT, MAP; 1 km resolution) and soil (total exchangeable bases, C:N ratio, soil texture; 0–30 cm depth). We ran all predictor subsets and averaged models where cumulative AIC_C_ weights summed to 0.95. Residual spatial autocorrelation was present, so we repeated the analysis using simultaneous autoregressive error models to explicitly model spatially autocorrelated errors. We also repeated the analysis using structural equation models implemented in the R package lavaan^51^. Relationships amongst 0.04 ha subplots in the 266 plots where subplot level data were also available were examined using multiple regressions of ln(carbon) against ln(diversity) and ln(stem density) for each plot individually, as well as for all plots using a random coefficients mixed effect model with plot identity as a random effect. Finally, we conducted a series of analyses to assess support for possible mechanisms driving diversity-carbon relationships, which are described in full in the Supplementary Discussion. Briefly, we produced separate models of carbon stocks as a function of the community weighted mean (CWM) of wood density, the CWM of maximum stem diameter, the standard deviation of wood density and a functional diversity metric including both these traits. Relationships at 1 ha were modelled using linear regression, relationships at 0.04 ha were modelled using linear mixed effects models with plot identity as a random effect. We related the expected probability of sampling a species with large potential size or high wood density (defined as maximum diameter ≥70 cm or wood density ≥0.8 g.cm^−3^ respectively, other thresholds were also examined) to species richness using null models, and also used binomial generalised linear mixed-effects models to relate the occurrence of these species in 0.04 ha subplots to species richness. Significance testing is based on two-tailed tests, with α = 0.05 used to determine statistical significance. See Supplementary Methods for full details of methods.

### Data availability

Data are available from http://dx.doi.org/10.5521/FORESTPLOTS.NET/2016_3.

## Additional Information

**How to cite this article**: Sullivan, M. J. P. *et al*. Diversity and carbon storage across the tropical forest biome. *Sci. Rep.* **7**, 39102; doi: 10.1038/srep39102 (2017).

**Publisher's note:** Springer Nature remains neutral with regard to jurisdictional claims in published maps and institutional affiliations.

## Supplementary Material

Supporting Material

## Figures and Tables

**Table 1:** Pan-tropical and continental studies assessing the diversity-carbon relationship.

Study	Geographical scope	Number of plots		Number of sampling locations				Taxonomic level	Diversity measures	Minimum identification level	Diversity-carbon relationship	
		1 ha	0.04 ha	Total	Amazon	Congo	Borneo				Within stand	Among stands
**This study**	**Tropics**	**360**	**6536**	**166**	**77**	**52**	**18**	**Species, genus and family**	**Richness, rarefied richness, Shannon diversity, Simpson diversity, Fisher’s alpha and functional diversity**	**80% stems to genus, 60% to species**	**+**	**None**
Ref. 22	Tropical and temperate	688^a^	17200^a^	25	2	1	1	Species	Richness^b^	None given	+	[None]
Ref. 24	Tropics	59	NA	11	3	2	0	Genus	Richness, Shannon diversity, functional diversity	80% stems to family	NA	+
Ref. 23	Tropical America	294	1975^d^	59	47	0	0	Species	Richness, rarefied richness and Shannon diversity	None given	+	+^e^

Sampling locations are groups of plots in close proximity to each other (individual large plots in ref. 22, TEAM study sites in ref. 24, “forest sites” in ref. 23, groups of plots within 5 km of each other in this study). The number of sampling locations in the largest blocs of forest in each continent are given, these are the Amazon basin and surrounding contiguous forest, the Congo basin and surrounding contiguous forest, and Borneo. + indicates a positive diversity-carbon relationship, NA indicates the relationship was not studied at the given scale. In this study, ref. 22 and ref. 24 all stems ≥10 cm d.b.h. were measured, in ref. 23 the minimum stem diameter measured varied among plots (either 5 cm or 10 cm).

^a^Sample size not stated, so maximum possible number of 1 ha and 0.04 ha subplots given.

^b^Stem density was included as a covariate in analysis.

^c^Relationship analysed among 1 ha plots within sampling locations, not among sampling locations.

^d^0.1 ha not 0.04 ha.

^e^Relationship among sampling locations.

**Table 2:** Mean carbon stocks per unit area and tree diversity in forest inventory plots in South America (n = 158), Africa (n = 162) and Asia (n = 40).

Variable	South America	Africa	Asia
Carbon (Mg ha^−1^)	140 (133–148)^A^	183 (176–190)^B^	197 (180–215)^B^
Fisher’s α	80 (71–88)^B^	28 (26–30)^A^	84 (73–96)^B^
Species richness (ha^−1^)	152 (141–163)^B^	74 (70–78)^A^	162 (147–177)^B^
(300 stems^−1^)	109 (102–116)^B^	65 (62–69)^A^	120 (111–130)^B^
Genus richness (ha^−1^)	91 (86–96)^B^	59 (56–62)^A^	87 (81–93)^B^
(300 stems^−1^)	72 (68–75)^B^	54 (51–56)^A^	71 (66–75)^B^
Family richness (ha^−1^)	38 (37–39)^B^	28 (27–28)^A^	40 (38–42)^B^
(300 stems^−1^)	33 (32–34)^B^	26 (25–27)^A^	35 (34–37)^B^

95% confidence limits derived from 10,000 bootstrap resamples of the data (sampling with replacement) are shown in parentheses. Different letters indicate significant differences between continents (ANOVA and subsequent Tukey’s all-pair comparison, *P* < 0.05). Data for other diversity metrics shown in Supplementary Table 2.

**Table 3:** Correlations (Kendall’s τ) between carbon and tree diversity in South America (n = 158 plots), Africa (n = 162) and Asia (n = 40).

Diversity metric	South America		Africa		Asia	
	τ	*P-value*	τ	*P-value*	τ	*P-value*
Fisher’s α	0.083	0.12	0.012	0.821	0.115	0.302
Species richness	0.084 (0.092)	0.12 (0.087)	0.014 (0.031)	0.788 (0.573)	0.132 (0.151)	0.230 (0.174)
Genus richness	0.066 (0.059)	0.223 (0.272)	−0.016 (0.01)	0.765 (0.859)	−0.006 (−0.051)	0.954 (0.652)
Family richness	−0.007 (−0.042)	0.893 (0.43)	−0.051 (−0.036)	0.35 (0.519)	0.087 (0.021)	0.434 (0.862)
Detectable effect size	τ = 0.14 *r* = 0.22		τ = 0.14 *r* = 0.22		τ = 0.28 *r* = 0.43	

Power analysis was used to estimate the minimum effect size (presented as both τ and Pearson’s *r*) detectable with 80% power. Correlations with taxon richness per 300 stems are shown in parentheses. Correlations with other diversity metrics shown in Supplementary Table 4.
