# Supplementary material for: Diversity and carbon storage across the tropical forest biome
Source: Sci Rep. 2017 Jan 17;7:39102. doi: 10.1038/srep39102 (PMC5240619; doi:10.1038/srep39102)
Supplement: Supporting Material [file srep39102-s1.pdf]

Supporting material for Sullivan et al. Diversity and carbon storage across the tropical forest biome

Martin J. P. Sullivan[a,1,*], Joey Talbot[a, 1], Simon L. Lewis[a,b, 1], Oliver L. Phillips[a, 1], Lan Qie[a], Serge K. Begne[a,c], Jerôme Chave[d], Aida Cuni Sanchez[b], Wannes Hubau[a], Gabriela Lopez-Gonzalez[a], Lera Miles[e], Abel Monteagudo-Mendoza[f,g], Bonaventure Sonké[c], Terry Sunderland[h,i], Hans ter Steege[j,k], Lee J. T. White[l,m,n], Kofi Affum-Baffoe[o], Shin-ichiro Aiba[p], Everton C. Almeida[q], Edmar Almeida de Oliveira[kkk], Patricia Alvarez-Loayza[fff], Esteban Álvarez Dávila[kk], Ana Andrade[r], Luiz E.O.C. Aragão[s], Peter Ashton[t], Gerardo A. Aymard C.[u], Timothy R. Baker[a], Michael Balinga[v], Lindsay F. Banin[w], Christopher Baraloto[x], Jean-Francois Bastin[y,z], Nicholas Berry[aa], Jan Bogaert[bb], Damien Bonal[cc], Frans Bongers[dd], Roel Brienen[a], , José Luís C. Camargo[ee], Carlos Cerón[ff], Victor Chama Moscoso[g], Eric Chezeaux[gg], Connie J. Clark[hh], Álvaro Cogollo Pacheco [ppp], James A Comiskey[ii], Fernando Cornejo Valverde[jjjj], Eurídice N. Honorio Coronado[jj], Greta Dargie[a], Stuart J. Davies[ll], Charles De Canniere[mm], Marie Noel Djuikouo K.[nn], Jean-Louis Doucet[oo], Terry L. Erwin[pp], Javier Silva Espejo[g], Corneille E.N. Ewango[qq,rr], Sophie Fauset[a,ss], Ted R. Feldpausch[s], Rafael Herrera[tt,uu], Martin Gilpin[a], Emanuel Gloor[a], Jefferson Hall[vv], David J. Harris[ww], Terese B. Hart[xx,yy], Kuswata Kartawinata[zz,aaa], Lip Khoon Kho[bbb], Kanehiro Kitayama[ccc], Susan G. W. Laurance[ddd], William F. Laurance[ddd], Miguel E. Leal[eee], Thomas Lovejoy[ggg], Jon C.Lovett[a], Faustin Mpanya Lukasu[hhh], Jean-Remy Makana[qq], Yadvinder Malhi [iii], Leandro Maracahipes[jjj], Beatriz S. Marimon[kkk], Ben Hur Marimon Junior[kkk], Andrew R. Marshall[lll,mmm], Paulo S. Morandi[kkk], John Tshibamba Mukendi[hhh], Jaques Mukinzi[qq,nnn], Reuben Nilus[ooo], Percy Núñez Vargas[g], Nadir C. Pallqui Camacho[g], Guido Pardo[qqq], Marielos Peña-Claros[rrr], Pascal Pétronelli[sss], Georgia C. Pickavance[a], Axel D. Poulsen [ttt], John R. Poulsen[hh], Richard B. Primack[uuu], Hari Priyadi[vvv,www], Carlos A. Quesada[r], Jan Reitsma[xxx], Maxime Réjou-Méchain[d], Zorayda Restrepo Correa[kk], Ervan Rutishauser[yyy], Kamariah Abu Salim[zzz], Rafael P. Salomão[aaaa], Ismayadi Samsoedin[bbbb], Douglas Sheil[h,cccc], Rodrigo Sierra[dddd], Marcos Silveira[eeee], J. W. Ferry Slik[zzz], Lisa Steel[ffff], Hermann Taedoumg[c], Sylvester Tan[gggg], John W. Terborgh[hh], Sean C. Thomas[hhhh], Marisol Toledo[rrr], Peter Umunay[iiii], Luis Valenzuela Gamarra[f], Ima Célia Guimarães Vieira[aaaa], Vincent A. Vos[qqq,], Ophelia Wang[kkkk], Simon Willcock[llll], Lise Zemagho[c]

Author affiliations:

[a]School of Geography, University of Leeds, Leeds, UK, [b]Department of Geography, University College London, London, UK,[c]Plant Systematic and Ecology Laboratory, University of Yaounde I, Cameroon, [d]Université Paul Sabatier CNRS, Toulouse, France, [e]United Nations Environment Programme World Conservation Monitoring Centre, Cambridge, UK, [f]Jardín Botánico de Missouri, Oxapampa, Perú,[g]Universidad Nacional de San Antonio Abad del Cusco, Cusco, Perú, [h]CIFOR, Bogor, Indonesia, [i]College of Marine and Environmental Sciences, James Cook University, Australia, [j]Naturalis Biodiversity Center, Leiden, Netherlands,[k]Ecology and Biodiversity Group, Utrecht University, Utrecht, Netherlands, [l]Agence Nationale des Parcs Nationaux, Libreville, Gabon,[m]Institut de Recherche en Ecologie Tropicale, Libreville, Gabon,[n]School of Natural Sciences, University of Stirling, Stirling, UK, [o]Mensuration Unit, Forestry Commission of Ghana, Kumasi, Ghana, [p]Graduate School of Science and Engineering, Kagoshima University, Japan, [q]Instituto de Biodiversidade e Floresta, Universidade Federal do Oeste do Pará, Santarém, Brazil, [r]Instituto Nacional de Pesquisas da Amazônia, Manaus, Brazil, Geography, College of Life and Environmental Sciences, University of Exeter, Exeter, UK, [t]Department of Organismic and Evolutionary Biology, Harvard University, Cambridge, MA, USA, [u]Programa de Ciencias del Agro y el Mar, Herbario Universitario, Venezuela, [v]CIFOR, Guinea, [w]Centre for Ecology and Hydrology, Penicuik, UK, [x]INRA, UMR 'Ecologie des Forêts de Guyane', France, [y]UMR AMAP, IRD, Montpellier, France, [z]UPR BSEF, CIRAD, Montpellier, France, [aa]The University of Edinburgh, School of GeoSciences, Edinburgh, UK, [bb]Biodiversity and Landscape Unit, Gembloux Agro-Bio Tech, Université de Liège, Gembloux, Belgium, [cc]INRA, UMR EEF, Champenoux, France, [dd]Forest Ecology and Forest Management group, Wageningen University, Wageningen, The Netherlands, [ee]Instituto Nacional de Pesquisas da Amazônia, Projeto Dinâmica Biológica de Fragmentos Florestais, Manaus, Brazil, [ff]Herbario Alfredo Paredes, Universidad Central del Ecuador, Quito, Ecuador, [gg]Rougier-Gabon, Libreville, Gabon, [hh]Nicholas School of the Environment, Duke University, Durham, NC, USA, [ii]Inventory & Monitoring Program, National Park Service, Fredericksburgh, VA, USA, [jj]Instituto de Investigaciones de la Amazonia Perúana, Iquitos, Perú, [kk]Servicios Ecosistémicos y Cambio Climático, Jardín

Botánico de Medellín, Medellín, Colombia, [ll]Smithsonian Tropical Research Institute, Washington, DC, USA, [mm]Landscape Ecology and Vegetal Production Systems Unit, Universite Libre de Bruxelles, Brussels, Belgium, [nn]Department of Botany & Plant Physiology, Faculty of Science, University of Buea, Buea, Cameroon, [oo]Forest Ressources Management, Gembloux Agro-Bio Tech, University of Liege, Belgium, [pp]Smithsonian Institution, Washington, DC, USA, [qq]Wildlife Conservation Society-DR Congo, Kinshasa I, Democratic Republic of Congo, [rr]Centre de Formation et de Recherche en Conservation Forestiere (CEFRECOF), Democratic Republic of Congo, [ss]Institute of Biology, UNICAMP, Campinas, Brazil , [tt]Centro de Ecologia, Instituto Venezolano de Investigaciones Cientificas, Caracas, Venezuela, [uu]Institut für Geographie und Regionalforschung, Geoökologie, University of Vienna, Austria, [vv]Smithsonian Tropical Research Institute, Panamá, Republic of Panama, [ww]Royal Botanic Garden Edinburgh, Edinburgh, UK, [xx]Lukuru Wildlife Research Foundation, Kinshasa, Gombe, Democratic Republic of Congo, [yy]Division of Vertebrate Zoology, Yale Peabody Museum of Natural History, New Haven, CT, USA, [zz]Herbarium Bogoriense, Indonesian Institute of Sciences, Indonesia, [aaa]Integrative Research Center, The Field Museum, Chicago, IL, [bbb]Tropical Peat Research Institute, Biological Research Division, Malaysian Palm Oil Board, Selangor, Malaysia, [ccc]Kyoto University, Kyoto, Japan, [ddd]Centre for Tropical Environmental and Sustainability Sciences and College of Marine and Environmental Sciences, James Cook University, Cairns, Australia, [eee]Wildlife Conservation Society, Kampala, Uganda, [fff]Center for Tropical Conservation, Duke University, Durham, NC, USA, [ggg]Department of Environmental Science and Policy, George Mason University, Fairfax, VA, USA, [hhh]Faculté des Sciences Agronomiques, Université de Kisangani, Democratic Republic of Congo, [iii]School of Geography and the Environment, University of Oxford, Oxford, UK, [jjj]Universidade Federal de Goiás, Goiânia, Brazil, [kkk]Universidade do Estado de Mato Grosso, Nova Xavantina, Brazil, [lll]Flamingo Land Ltd, Kirby Misperton, UK, [mmm]CIRCLE, Environment Department, University of York, York, UK, [nnn]Salonga National Park, Kinshasa I, DR Congo, [ooo]Sabah Forestry Department, Sabah, Malaysia, [ppp]Jardín Botánico Joaquín Antonio Uribe, Medellín, Colombia, [qqq]Universidad Autónoma del Beni, Riberalta, Bolivia, [rrr]Instituto Boliviano de Investigación Forestal, Santa Cruz de la Sierra, Bolivia,[sss]CIRAD, UMR Ecologie des Forêts de Guyane, France, [ttt]Natural History Museum, University of Oslo, Oslo,

Norway, [uuu]Department of Biology, Boston University, Boston, MA, [vvv]CIFOR, Bogor, Indonesia, [www]Southern Swedish Forest Research Center, Swedish University of Agricultural Sciences, Alnarp, Sweden, [xxx]Bureau Waardenburg, The Netherlands, [yyy]Carboforexpert, Geneva, Switzerland, [zzz]Faculty of Science, Universiti Brunei Darusallam, Gadong, Brunei, [aaaa]Museu Paraense Emilio Goeldi, Belém, Brazil, [bbbb]FORDA, The Ministry of Forestry and Environment, Bogor, Indonesia, [cccc]Norwegian University of Life Sciences, Aas, Norway [dddd]GeoIS, Quito, Ecuador, [eeee]Museu Universitário, Universidade Federal do Acre, Brazil, [ffff]World Wildlife Fund, Washington, DC, USA, [gggg]CTFS-AA Asia Program, Harvard University, Cambridge, MA, USA, [hhhh]Faculty of Forestry, University of Toronto, Toronto, Canada, [iiii]Yale School of Forestry & Environmental Studies, New Haven, CT, USA, [jjjj]Andes to Amazon Biodiversity Program, Puerto Maldonado, Perú, [kkkk]School of Earth Sciences and Environmental Sustainability, Northern Arizona University, Flagstaff AZ, USA, [llll]Department of Life Sciences, University of Southampton, Southampton, UK

Contents

Supplementary Methods: Page 6

Supplementary Discussion: Examining support for mechanisms underpinning diversity-carbon relationships – includes Supplementary Figure 1 – Supplementary Figure 10: Page 13

Supplementary Figure 11. Increase in diversity (Fisher's α) with increasing sample size of plots and increasing geographic distance around plots: Page 29

Supplementary Figure 12. Coefficients of generalised linear models of species similarity (Sørensen index) against distance: Page 30

Supplementary Figure 13. Variation in beta deviation among continents. Page 31

Supplementary Figure 14. Relationship between carbon and each diversity metric: Page 32

Supplementary Figure 15: Average model coefficients from simultaneous autoregressive error model of carbon storage in 1 ha plots as a function of species richness, climate, and soil: Page 33

Supplementary Figure 16: Relationship between carbon storage and stand structure: Page 34

Supplementary Figure 17: Relationship between carbon storage, species richness and stem size inequality: Page 35

Supplementary Figure 18: Fitted structural equation model: Page 36

Supplementary Figure 19. Coefficients of relationships between diversity metrics and carbon between 1 ha plots within continents: Page 37

Supplementary Table 1. Loadings of first two axes of principal components analysis performed on soil texture data from each 1 ha plot: Page 38

Supplementary Table 2. Mean carbon storage and diversity in 1ha plots in South America, Africa and Asia: Page 39

Supplementary Table 3. Mean carbon storage and tree diversity in forest inventory plots where at least 90% of stems have been identified to species level: Page 40

Supplementary Table 4. Kendall's tau correlations between carbon and diversity metrics in each continent: Page 41

Supplementary Table 5. Averaged multiple regression model for ln(carbon) in South American plots as a function of species richness, climate and soil variables : Page 42

Supplementary Table 6: Averaged simultaneous autoregressive model coefficients for ln(carbon) in 1 ha plots as a function of the community weighted mean of wood density, species richness, climate and soil variable: Page 50

Supplementary Table 7. Relationship between diversity and carbon in 0.04ha subplots within 1ha forest inventory plots: Page 51

Supplementary methods

Plot selection

Plots were obtained from a global dataset of forest inventory plots [1] surveyed using standardised field methods [2]. Plots were 1 ha (except for four that were 0.96ha), and were all located in old-growth, closed-canopy, *terra firme* forests, with mean annual temperature of ≥20°C and mean annual precipitation of ≥1300mm. Thus, montane, swamp, peatland and seasonally flooded forest were excluded. Plots known to have been subject to anthropogenic disturbance were also excluded. This enabled us to focus on carbon-diversity relationships within lowland *terra firme* tropical forest, avoiding major climatic, anthropogenic and hydrological factors that could confound these relationships. Having accurate measures of diversity was important for the purposes of this study, so plots were only included if >80% of trees were identified to genus level and >60% of trees were identified to species level. Identification rates were similar amongst continents (median identification rates to species level: South America = 92.5%, Africa = 93.5%, Asia = 93.1%). We excluded transects >500m in length or <20m in width, and any plot known to contain more than one soil type, and only included non-contiguous samples if within 500m of each other. In each plot all stems ≥100mm diameter were measured, and identified to species level where possible. Where a plot had been surveyed multiple times we normally used the initial census, as these were typically accompanied by botanists so were expected to have the highest proportion of identified stems, except where there was a specific reason (e.g. failure of first census to meet selection criteria) to use a later census.

Environmental variables

We used soil data from 0-30 cm depth, and used total exchangeable bases (TEB; measuring soil fertility), carbon: nitrogen ratio (C:N ratio; a useful proxy of available phosphorus) and soil texture as explanatory variables in analysis. Plots were assigned a reference soil group according to the World Reference Base soil classification system [3], using data from published sources e.g. [4,5] where available. When these data were not available, the reference soil group as mapped in the Harmonised World Soil

Database [6], or SOTER [7] for the Democratic Republic of the Congo, was used. Results are similar when only dominant soil groups are used (Supplementary Fig. 20). Then, the particle size and TEB data for the nearest soil unit of the same reference soil group were extracted from the HWSD or SOTER. C:N data were extracted from the Digital Soil Map of the World, or SOTERLAC or SOTER where available.

We extracted mean annual precipitation (MAP) and mean annual temperature (MAT) from the WorldClim database [8] at 30’ (≈ 1km) resolution. Temperature data were corrected using the lapse rate Δ temperature = 0.005°C m^{-1} to account for differences between plot elevation and the mean elevation of WorldClim grid-cells. We also calculated cumulative water deficit (CWD), a measure of water stress experienced in the dry season. This was done using mean monthly precipitation from WorldClim and mean monthly potential evapotranspiration (PET, 1980-2010 average) from CRU TS3.22 [9]. The water balance for each month (t) was calculated as $CWD_t = \min(0, CWD_{t-1} + Precipitation_t - PET_t)$. This model was run recursively over a period of 12 months, starting in the wettest month of the year, with the starting water balance assumed to be zero. The minimum CWD_t value across the year represents the greatest drought stress experienced by plants, and is referred to as CWD.

Estimating diversity

Although we applied stringent selection criteria to ensure that the diversity measures included in this study were largely based on fully identified taxa, it was seldom possible to fully identify all taxa in a plot, as local species pools frequently exceed 1000 tree taxa in the tropical forest domain [10]. Some unidentified stems could safely be considered to be additional taxa and added to richness estimates as botanists had assigned them to morphospecies, or had identified them to a higher taxonomic level not otherwise represented in the plot. We assigned remaining unidentified stems to discrete taxa based on the ratio of taxa per stem based on stems that were fully identified to a given taxonomic level. This procedure was necessary to ensure that richness estimates did not simply reflect the proportion of

stems that could be fully identified. The formulas for deriving richness estimates at different taxonomic levels are thus:

Species richness = $I_s + M_s + a + b + [U_s \, . \, P_s]$,

Genus richness = $I_g + a + [U_g \, . \, P_g]$,

Family richness = $I_f + [U_f \, . \, P_f]$,

Where I = richness of stems identified to a given taxonomic level, M_s = morphospecies richness, a = richness of stems unidentified to genus level but unique representatives of a particular family, b = richness of stems unidentified to species level but unique representatives of a particular genus, U =number of stems remaining unidentified at a given taxonomic level, P = number of taxa (at given taxonomic level) per identified stem, and s, g and f subscripts denoting species, genus and family respectively. [] denotes rounding to the nearest integer.

These formulas give richness per unit area. Richness per n stems was estimated using individual based rarefaction at both plot (1 ha) and subplot (0.04 ha) scales. At plot scale, richness was expressed per 300 stems, while at subplot scale richness was expressed per 10 stems.

We calculated diversity metrics representing the three most commonly used Hill numbers [11], richness (^0D), Shannon diversity ($^1D = \exp(H')$, where $H' = -\sum p_i \log p_i$, with p_i the proportion of stems belonging to species i) and Simpson diversity ($^2D = 1/\lambda$, where $\lambda = \sum p_i^2$), as these give different weightings to rare versus dominant taxa, with higher Hill numbers giving greater proportional weight to dominant taxa. In addition, we calculated Fisher's α, as it is commonly used to explore diversity in tropical forests. Fisher's α is a constant derived from the log series $S = \alpha \ln(1+N/\alpha)$, where S is the number of species in the sampled community and N is the number of individuals sampled. Analyses with taxon richness (^0D) and Fisher's α have been presented in the main text, with analysis of ^1D and ^2D presented in supporting materials.

Analysing beta diversity

We used Sørensen index to quantify beta diversity between pairs of plots. These pairwise similarities were related to the geographic distance between pairs of plots using a generalised linear model with a binomial errors and a log-link function following [12]. Fitting exponential distance decay models as generalised linear models in this way avoids the problem of log-transforming zero similarity values, with a binomial error structure appropriate as similarity values are bounded to vary between zero and one [12]. Models were constructed for each continent. The significance of parameter estimates was assessed by resampling the data 10000 times with replacement. Following [12] we excluded identical sites pairs with zero geographic distance and identical tree communities from bootstrap samples as these lie outside the original sampling frame.

We also investigated how Fisher's alpha in each continent increased with the number of samples or the distance around a plot, repeating the methods of [13] on our dataset to investigate whether the patterns of diversity accumulation over space they observe are also evident in our data.

Incomplete species identifications pose a challenge to the calculation of beta diversity as it means that not all the species pool have been sampled. A wide range of beta diversity metrics, including Sørensen index, show an approximately linear relationship between undersampling of taxa and bias in the beta diversity metric [14]. Because of this we excluded sites with <90% of stems identified to species level from our analysis of beta diversity; this threshold was a compromise between maintaining a large sample of plots and reducing bias caused by undersampling of taxa. This threshold gave a sample size of 99 plots in South America, 105 plots in Africa and 23 plots in Asia. Synonymous species names pose a further challenge, as treating two synonyms as separate species would inflate beta diversity, and no universal adjudicated list exists for all tropical plants. We used the R package Taxonstand [15] to compare species names with those in The Plant List (www.theplantlist.org) and remove identified synonyms. However, 28.5% of identified stems remained unresolved (i.e. the species name was present in The Plant List but it was uncertain whether the species name was a synonym) in Asia after using Taxonstand, compared to 0.3% in South America and 0.6% in Africa,

indicating that further botanical work is required in Asia to resolve these synonyms. We compared unresolved species in Asia against The Asian Plant Synonym Lookup (phylodiversity.net/fslik/synonym_lookup.htm) in a further attempt to remove synonyms. Following this, 5.2% of identified stems in Asia remained unresolved.

Statistical analysis

We conducted analyses at three spatial scales, firstly comparing carbon and diversity among continents, secondly, assessing relationships between carbon and diversity between 1ha plots within each continent, and finally assessing carbon – diversity relationships between 0.04 ha subplots within 1 ha plots.

Differences in carbon-storage and diversity metrics between continents were assessed by modelling each response variable of interest as a function of continent in a linear modelling framework, where continent was a factor with three levels. Area based taxon richness are count data, so were modelled using generalised linear models with negative binomial errors (due to overdispersion) and a log link function. ^{1}D (species level), ^{2}D (species level) and Fisher's α were square root transformed prior to modelling to homogenise variances and ensure normality of residuals. We tested for significant differences between continents using Tukey's all-pair comparisons, implemented in the R packages multcomp [16].

We then conducted Kendall's tau correlations between carbon and each diversity metric to assess univariate relationships, using plot level data from each continent in turn. Kendall's tau was chosen as it is non-parametric, so does not assume bivariate normality, and can handle ties. This analysis involved computing 13 tests for each continent, so there is therefore some risk of significant relationships appearing by chance. We used false discovery rate control to adjust *P* values for multiple testing, and present both corrected and uncorrected *P* values. We performed power analysis using the R package pwr [17] to assess the smallest effect size (Pearson's *r*) that could be detected with 80% power given the sample size in each continent. Values of *r* were converted to τ using the lookup table in [18].

The univariate correlations examined whether diversity metrics were spatially congruent with carbon. However, other environmental variables acting on carbon or diversity metrics could enhance or obscure any underlying mechanistic relationship. We therefore conducted a multivariate analysis where carbon was modelled as a function of diversity metrics, climate and edaphic variables. This analysis was performed separately for each continent. Diversity metrics were highly correlated with each other (mean Pearson's $r = 0.833$), so one model was constructed per diversity metric. We included cumulative water deficit (CWD), mean annual temperature (MAT), mean annual precipitation (MAP) as climate variables; we did not include other variables relating to precipitation seasonality as they were strongly correlated with CWD. No plots in Asia experienced CWD different from zero, so CWD was not included in models for there. We used Principal Component Analysis to collapse variation in soil texture into two orthogonal axes, which collectively explained 95.4% of variation in soil texture. Axis one (PCA1) was positively correlated with the amount of sand, while axis two (PCA 2) was correlated with the amount of silt and negatively correlated with the amount of clay (Supplementary Table 1). We also included the sum of total exchangeable bases (TEB) and the carbon: nitrogen ratio (C:N). Explanatory variables were centred and scaled to have a mean of zero and a standard deviation of one. The basic equation for these models was thus

$$\log(\text{carbon}) = a + \beta_1\text{Diversity metric} + \beta_2\text{CWD} + \beta_3\text{MAP} + \beta_4\text{MAT} + \beta_5\text{PCA1} + \beta_6\text{PCA2} + \beta_7\text{TEB} + \beta_8\text{C:N} + \varepsilon$$

We used MuMIn [19] to fit all valid simplifications of this global model. Each model was ranked based on AIC_C, from which the Akaike weight of each model i was calculated (ω_i). The parameters of the best supported models (defined as the models required for cumulative sum of w_i =0.95, known as the 95% confidence set) were averaged, while the support for individual explanatory variables was assessed by summing to ω_i of models in which that variable appeared.

Spatial autocorrelation in residuals of these OLS models was examined by plotting correlograms using the R package ncf [20]. Positive short range and negative long-range residual autocorrelation was evident in South America, suggesting the presence of strong environmental gradients. Residual spatial

autocorrelation was less strong in Africa, and weakest in Asia, but was present in all continents. We repeated the above modelling procedures using simultaneous autoregressive error models (SAR), implemented in spdep [21]. These were selected because of good performance in evaluations by [22], with error models selected, as opposed to lag or mixed SARs, as [23] found they performed better regardless of the mechanism generating spatial autocorrelation. We selected the best neighbourhood distance for each global model by fitting models with maximum neighbourhoods distances varying in 20km increments from 20km to 1000km, and selecting the neighbourhood distance that gave the lowest AIC_C. Although all SAR models had lower AIC values than OLS models, we present results from both OLS and SAR models, as it has been argued that spatial models are not necessarily more correct than non-spatial models [24].

We assessed fine-scale relationships between diversity and carbon by using multiple regression to model ln (carbon) in 0.04ha subplots as a function of diversity and the number of stems in the subplot, with a second order polynomial used for the number of stems to capture potentially saturating relationships. Explanatory variables were natural log transformed to allow comparison with results of [25]. We ran these models in each 1ha plot where subplot level was available (n = 266). We tested whether the mean coefficient was different from zero using one-sample Wilcoxon tests, and calculated 95% confidence intervals from 10000 bootstrap resamples with replacement. Running separate models for each plot allowed us to capture variability in fine scale relationships between plots. However, the overall mean relationship between diversity and carbon at subplot scale could be more robustly assessed using mixed effects models with random coefficients. This assumes that coefficients in plot j come from a normal distribution, $\beta_j \sim \text{Normal}(\mu_\beta\ \sigma^2_\beta)$, where μ_β is the mean value of the coefficient across plots, and σ^2_β is the variance of the coefficient across plots. We relax the assumption of independence between coefficients, so that pairs of coefficients in the same plot are assumed to come from a multivariate normal distribution with correlations between coefficients estimated in a variance-covariance matrix. Mixed effects models were implemented using the R package lme4 [26].

Supplementary Discussion – Examining support for mechanisms underpinning diversity-carbon relationships

Our results show a weak positive relationship between diversity and carbon storage at small spatial scales (among 0.04 ha subplots within 1 ha plots), but no pan-tropically consistent relationship among 1 ha plots, even after controlling for potentially confounding environmental variation and spatial autocorrelation. These results pose two questions. Firstly, which mechanisms underlie the positive diversity-carbon relationship between 0.04 ha subplots? And secondly, why do these mechanisms appear to only operate at small spatial scales? These questions are best investigated with long-term experiments in tropical forests, however, we can evaluate whether correlative results from our observational dataset are consistent with the operation of niche complementarity and selection effects at 0.04 ha and 1 ha scales.

Evidence for niche complementarity

Positive relationships between biodiversity and ecosystem function have been hypothesised to arise through two general mechanisms, niche complementarity and the selection effect. The niche complementarity hypothesis proposes that differences in resource use by species allows diverse communities to use available resources more efficiently than less diverse communities [27]. For example, in low diversity temperate forests, complimentary canopy architecture has been found to drive a positive relationship between diversity and productivity [28]. In tropical forests attempts to assess the role of niche complementarity have focused on relating above-ground live carbon storage to the functional diversity of tree communities [29,30], with the expectation that more functionally diverse species assemblages should be able to partition resources more effectively. However, these studies found no relationship between carbon storage and functional diversity [29,30].

Quantifying functional diversity in tropical forests is challenging due to the shortage of available trait data. We used two approaches to quantify functional diversity, (i) the standard deviation of wood density (SD_{WD}) in a subplot or plot, and (ii) a multivariate functional diversity metric (FDM) using both the wood density and the maximum diameter of each species in a subplot or plot.

For the SD_{WD} we used published wood density values [31,32], and commonly used methods to select genera-level wood density in cases when literature values for a given species were unavailable [33-35]. It would be preferable to use local trait data [36] but as these are not available for many plots it is necessary to use literature values for pan-tropical studies [29]. Wood density provides a proven proxy for life history strategy in tropical forests [37], since denser wooded trees tend to be slower growing, less light demanding and potentially larger than species with lower wood density [38,39], and variation in wood density is closely related to variation in leaf traits [40] and demographic traits [41]. We therefore expect that the potential for niche complementarity is greater in species assemblages with more variation in wood density.

The relationship between carbon storage and SD_{WD} at the 0.04 ha scale within 1-ha plots was variable but significantly negative overall. At the 1 ha scale the relationship was significantly negative in all three continents (Fig. S1). At both scales, SD_{WD} was negatively related to mean wood density (Fig. S2), indicating that the more variable plots were increasingly composed of species with ‘fast’ life history strategies. These plots potentially have high rates of stem turnover, and thus shorter biomass residence time [42]. When we included community weighted mean wood density as a covariate to account for this, the negative relationship between carbon storage and wood density standard deviation among 0.04 ha subplots was weaker but still significantly negative ($P < 0.001$). Negative relationships among 1 ha plots also weakened in all continents (non-significantly negative in South America and Africa ($P \geq 0.177$), significantly negative in Asia ($P = 0.004$). SD_{WD} was also negatively related to the community weighted mean of maximum diameter at both scales (Fig. S3), indicating that plots with a greater variety of tree life history strategies were increasingly composed of smaller tree species.

We then estimated functional diversity using the FDM, calculated following [43]. For this, we follow Cavanaugh et al. [29] and define functional diversity in terms of the wood density and maximum diameter of each species in an assemblage (they worked at genus level). Thus, following Fauset et al. [44], we estimated maximum diameter as the 95th percentile diameter of species with a least 20 stems in the dataset. We used species-level maximum diameters were available, with genus-level estimates

used for species that occurred too infrequently to estimate species level maximum diameter, and family-level estimates used when there was no genus-level estimate. We estimated maximum diameter for each genus and family using the same methods as for species-level estimates. We used this trait data to construct a functional dissimilarity matrix, where the dissimilarity of pairs of species based on their traits was quantified using Gower distance. This dissimilarity matrix was converted into a dendrogram using average linkage. FDM was calculated as the sum of branch lengths of a dendrogram containing all species in a plot or subplot divided by the sum of branch lengths of a dendrogram containing all species in the potential source pool, defined as all species in our dataset found in a given continent. Thus, FDM is equal to one when all the trait diversity in the source pool of species is found in in the subset of species in a subplot or plot, and decreases towards zero as increasingly large amounts of trait diversity are missing from the subset of species.

Our FDM metric showed an overall weak positive relationship between functional diversity and carbon storage at the 0.04ha scale (Fig. S1), which remained when community-weighted mean wood density was included as a covariate ($\beta = 2.6$, $P < 0.001$). However, at the 1 ha scale, FD and carbon storage were unrelated, even when community-weighted mean (CWM) wood density was included as a covariate ($P \geq 0.118$). This is consistent with results of previous studies at this scale [29,30], which found no relationship between functional diversity and carbon storage. At both scales FDM was weakly negatively related to community-weight mean wood density (Fig. S2), indicating that the most functionally diverse stands were composed of species with fast life-history strategies. FDM was positively related to the community weighted mean of maximum diameter at 0.04 ha scale but not at 1 ha scale. This indicates that at small scales trees are on average larger in more functionally diverse stands. The weak positive relationship between FDM and carbon storage at 0.04 ha but not 1 ha scale is consistent with the scale-dependent operation of niche complimentarily.

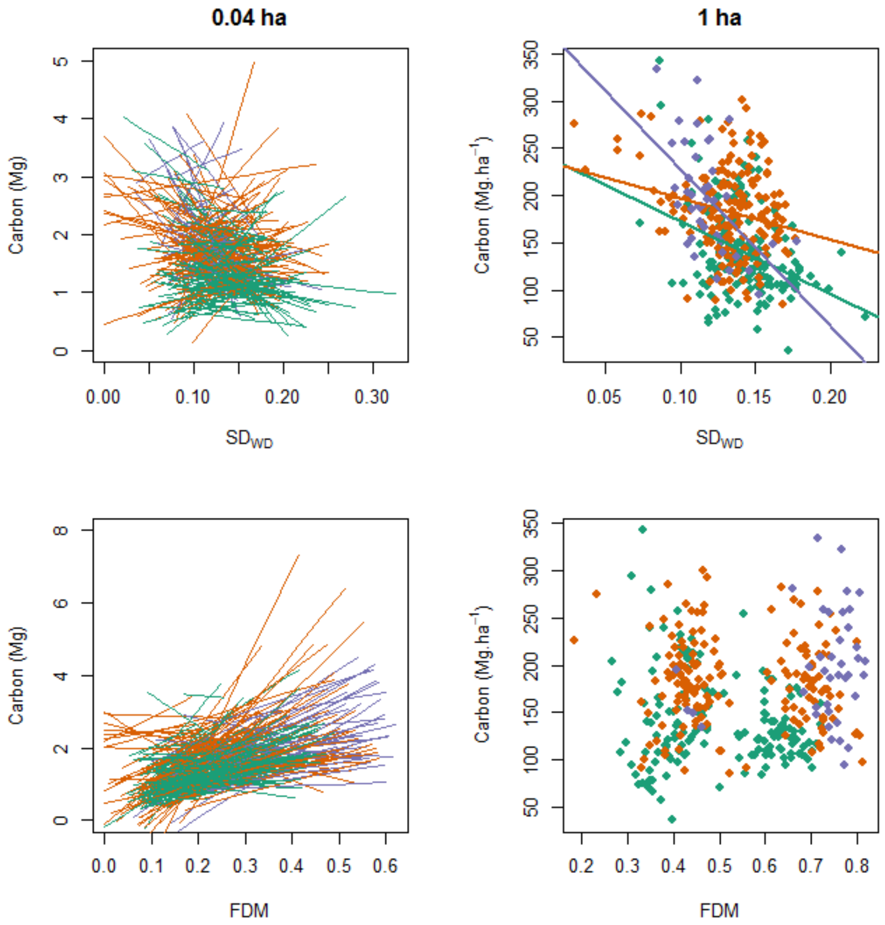

Supplementary Figure 1. Relationship between carbon storage and functional diversity. Functional diversity is quantified either as the standard deviation of wood density among stems within a plot/ sub-plot (SD_{WD}), or using a dendrogram based method where species are clustered according to their wood density and maximum diameter traits (FDM). Relationships are shown for 1 ha plots in each continent (data from South America are shown by green circles, Africa by orange squares, and Asia by purple triangles, regression lines are shown for significant relationships ($P < 0.05$)), and for 0.04 ha subplots in 1 ha plots (regression lines shown for each 1 ha plot, colour scheme same as before). Relationships between wood density SD and carbon are: South America 1 ha, $\beta = -5.0$, $P < 0.001$; Africa 1 ha $\beta = -2.3$, $P = 0.006$; Asia 1ha, $\beta = -9.1$, $P < 0.001$; 0.04 ha mixed effects model, $\beta = -1.1$, $P < 0.001$. Relationships between functional diversity and carbon are: South America 1 ha, $\beta = -0.3$, $P = 0.139$; Africa 1 ha, $\beta = -0.1$, $P = 0.687$, Asia 1 ha, $\beta = 0.5$, $P = 0.236$; 0.04 ha mixed effects model, $\beta = 2.5$, $P < 0.001$.

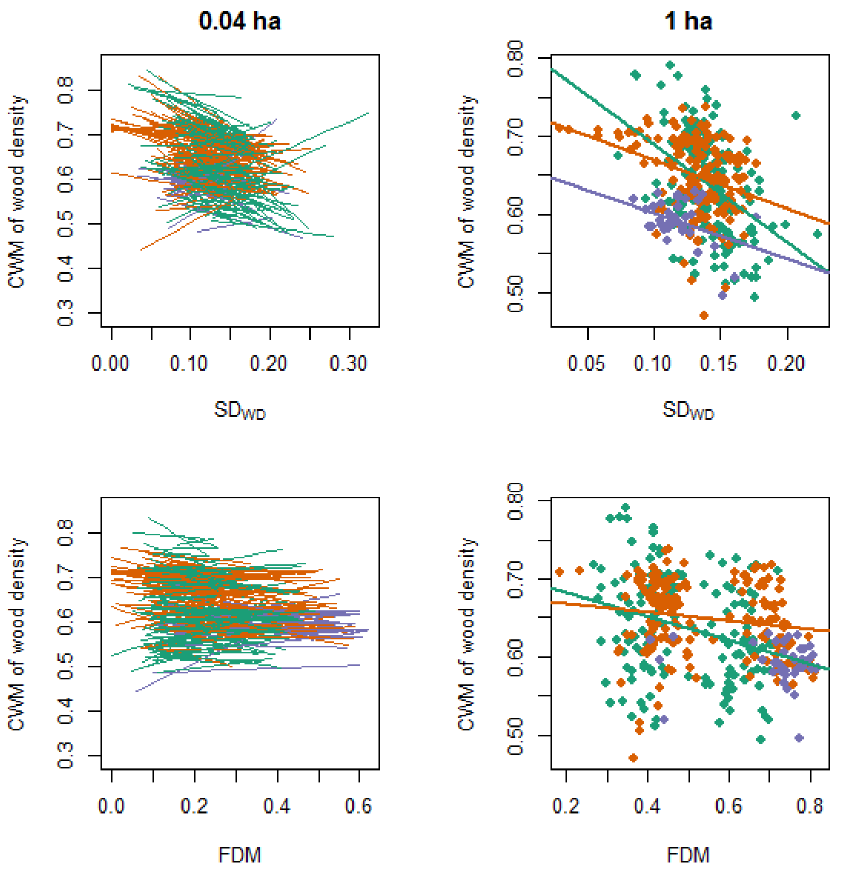

Supplementary Figure 2. Relationship between measures of functional diversity the community weighted mean of wood density. Symbols as in Fig. S1. Relationships between SD_{WD} and CWM of wood density are: South America 1 ha, β = -1.3, $P < 0.001$; Africa 1 ha, β = -0.6, $P < 0.001$; Asia 1 ha, β = -0.6, $P = 0.012$; 0.04 ha mixed effects model, β = -0.31, $P < 0.001$. Relationships between FDM and CWM of wood density are: South America 1 ha, β = -0.2, $P < 0.001$; Africa 1 ha, β = -0.1, $P = 0.0371$; Asia 1 ha, β = <-0.1, $P = 0.768$; 0.04 ha mixed effects model, β = -0.02, $P = 0.005$.

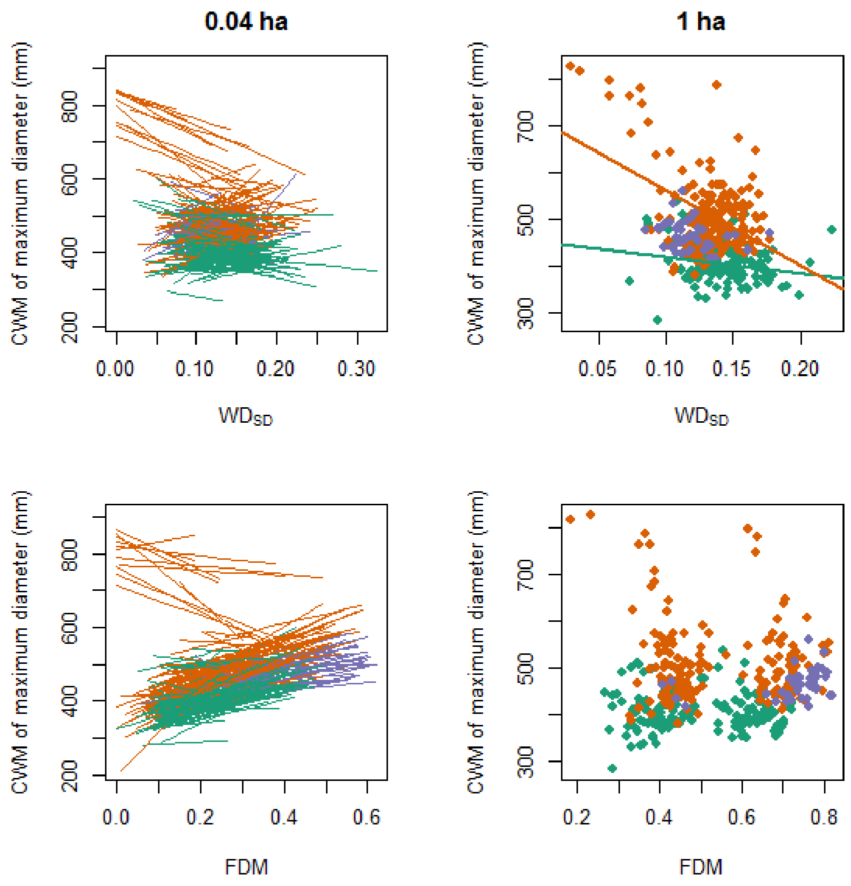

Supplementary Figure 3. Relationship between measures of functional diversity and the functional dominance of species with large maximum diameters. Symbols as in Fig. S1. Relationships between SD_{WD} and community-weighted mean (CWM) of maximum diameter are: South America 1 ha, β = -349, *P* = 0.0095; Africa 1 ha, β = -1623, *P* < 0.001; Asia 1 ha, β = -387, *P* = 0.156; 0.04 ha mixed effects model, β = -34, *P* < 0.001. Relationships between FDM and CWM of maximum diameter are: South America 1 ha, β = -33, *P* = 0.181; Africa 1 ha, β = -71, *P* = 0.137; Asia 1 ha, β = 84.7, *P* = 0.074; 0.04 ha mixed effect model, β = 263, *P* < 0.001.

<u>Evidence for the selection effect</u>

A second mechanism by which positive diversity-ecosystem function relationships might be generated is via the selection effect. The selection effect hypothesis proposes that diverse communities, by containing a greater proportion of the overall species pool, are more likely to contain dominant species that contribute strongly to ecosystem function [27]. Species contributions to ecosystem function are known to be highly uneven in Amazonian forests, where approximately 1% of species are responsible for 50% of carbon storage [44]. Furthermore, maximum diameter is known to be the most

important determinant of species' contribution to carbon storage [44], aside from overall abundance, while pan-tropically plot-level carbon stocks are closely related to the density of large trees (defined as d.b.h. ≥ 70 cm) [45]. Thus any positive diversity-carbon relationship could plausibly arise through diverse plots being more likely to contain species with large maximum diameters.

Previous attempts to evaluate whether selection effects occur in tropical forests have tested the prediction that carbon storage is related to the functional dominance of species with large maximum diameters or dense wood [29,30]. To begin with, we therefore repeated this approach with our larger pan-tropical dataset (360 plots), using the community weighted mean of wood density and maximum diameter as a measure of functional dominance. We found that at both scales carbon storage increased with the community weighted mean of maximum diameter, as found by previous studies at 1 ha scale [29,30], and also that it increased with the community weighted mean of wood density (Fig. S4), which the previous studies did not detect as a driver of carbon storage [29,30] . However, while this approach is useful and interesting, strictly it is a test of the biomass ratio hypothesis, by which ecosystem function is related to the traits of dominant taxa [46], rather than a test of the selection effect *per se*.

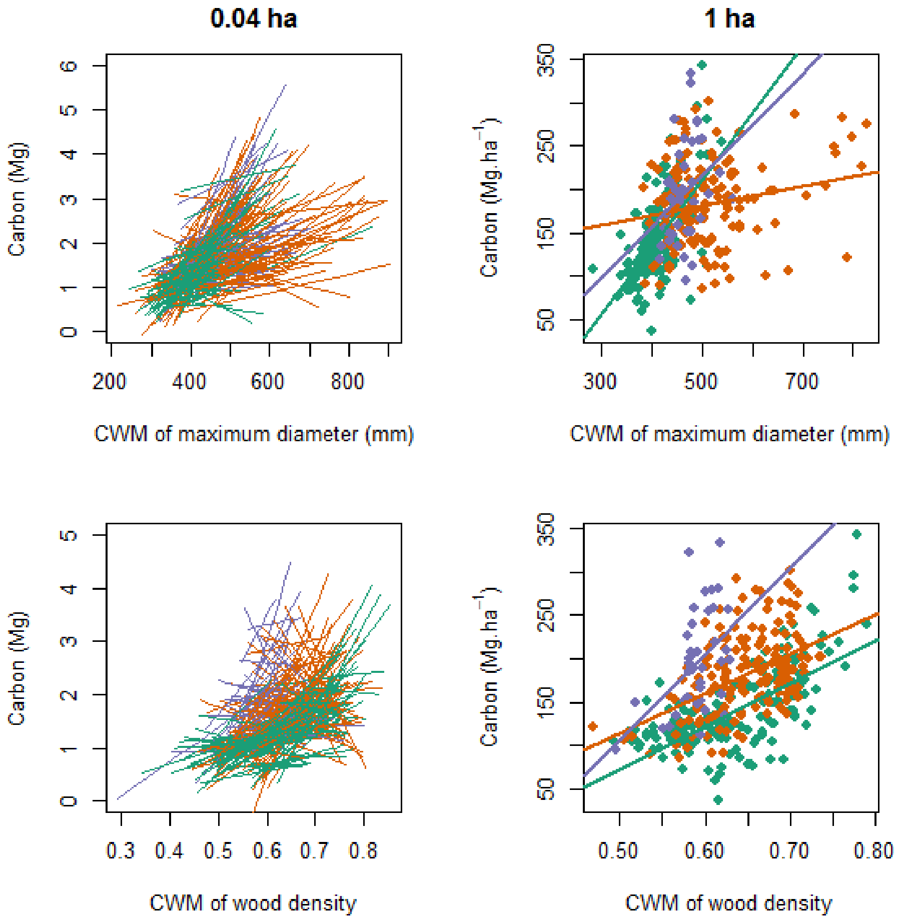

Supplementary Figure 4. Relationship between community weighted mean (CWM) of species traits and carbon storage. The community weighted mean of traits indicates the dominance of species with different trait values within a community. Symbols as in Fig. S1. Relationships between CWM of maximum diameter and carbon are: South America 1 ha, $\beta = 0.0049$, $P < 0.001$; Africa 1 ha, $\beta = 0.0006$, $P = 0.022$; Asia 1 ha, $\beta = 0.0030$, $P = 0.049$; 0.04 ha mixed effects model, $\beta = 0.0029$, $P < 0.001$. Relationships between CWM of wood density and carbon are: South America 1 ha, $\beta = 3.1$, $P < 0.001$; Africa 1 ha, $\beta = 2.8$, $P < 0.001$; Asia 1 ha, $\beta = 5.7$, $P = 0.0005$; 0.04 ha mixed effects model, $\beta = 2.4$, $P < 0.001$.

Another, and directly testable, prediction of the selection effect is that the probability of a community containing a functionally dominant species increases with species richness. Maximum diameter has been found to be an important determinant of species' contribution to carbon storage. Of our 1441 species for which the species level maximum diameter could be estimated, 169 (11.7%) had maximum diameters ≥ 70 cm. The probability of a random sample of *s* species from this species pool containing a potentially large tree species (L) is thus $L = 1 - 0.883^{s}$. This means that the probability of sampling a potentially large tree species rapidly saturates with species richness. For example, at 14

species (the median species richness of 0.04 ha subplots) $L = 0.826$, while at 100 species (the median species richness of 1 ha plots) $L > 0.999$. This calculation ignores differences in species composition between continents, so we also estimated the probability of samples of different species richness containing a potentially large species by sampling the tree species in our dataset 3000 times for each species richness increment, with each sample restricted to contain species from a single continent (1000 samples for each continent). We also repeated this procedure with the probability of sampling a species weighted by that species' frequency in a continent. Both approaches gave a similar rapidly saturating curve (Fig. S4), and with a slightly higher probability of sampling large species when species frequency was maintained. Importantly, the probability of a sample containing a potentially large species increases substantially through the inter-quartile range of 0.04 ha species richness values, but for the whole inter-quartile range of 1 ha species richness values samples were almost certain to contain a potentially large species (Fig. S5). Similar inferences obtain when we modelled the probability of subplots containing potentially large tree species was as a function of species richness using binomial generalised mixed models (with plot identity as a random effect): the probability of sampling a large tree species in a 20x20m subplot increased with species richness (Fig. S6), but at the 1 ha scale all but one of our 360 plots contains a potentially large tree species. This further supports the inference that selection effects could plausibly lead to a relationship between diversity and carbon storage at 0.04 ha scale, but that tropical forest 1 ha plots are sufficiently diverse for selection effects to have potentially saturated.

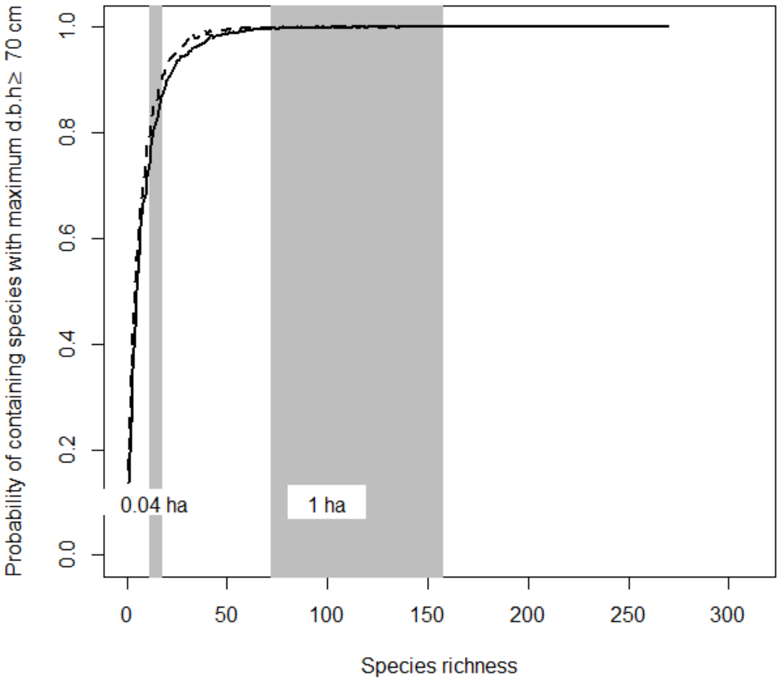

Supplementary Figure 5. Relationship between the species richness of a sample and the probability of that sample containing a potentially large tree species (maximum diameter ≥ 70 cm). Probabilities were estimated by randomly sampling the species pool in each continent 1000 times for each continent and species richness increment, with the probability of selecting a species either equal for all species in a continent (solid line) or weighted by the proportion of plots that species was recorded in (dashed line). The interquartile range of species richness in 0.04 ha subplots and 1 ha plots are shown by grey shading.

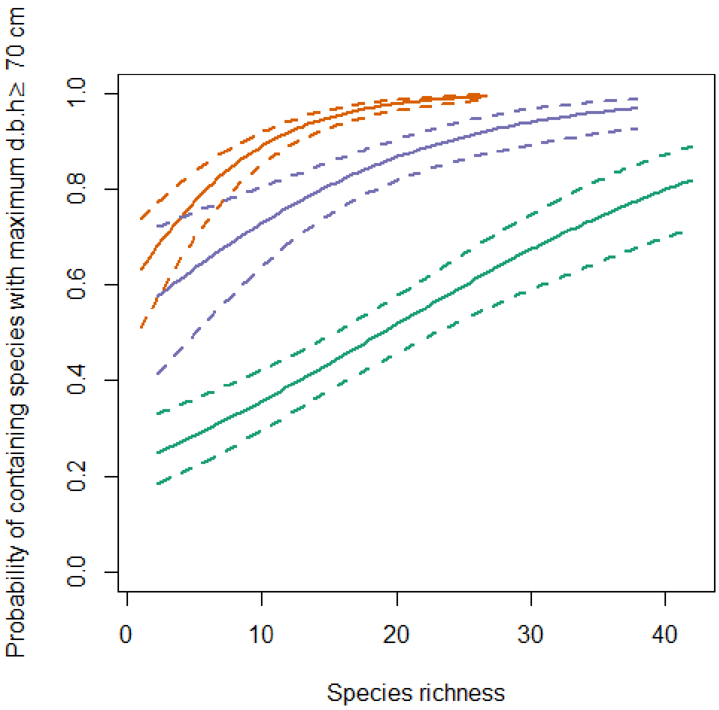

Supplementary Figure 6. Observed relationship between species richness of 0.04 ha subplots and the probability of that subplot containing a potentially large species (maximum diameter ≥ 70 cm). Fitted relationships in each continent are from generalised linear mixed effects models with binomial errors (green = South America, orange = Africa, purple = Asia). Standard errors are shown with dashed lines. Model coefficients are: South America, $\beta = 0.066$, $P < 0.001$; Africa, $\beta = 0.173$, $P < 0.001$; Asia, $\beta = 0.088$, $P < 0.001$. Relationships are not shown for 1 ha plots, as all but one of our 360 plots contained a potentially large species.

We find similar results when evaluating the probability of sampling a species with high wood density. Thus, the probability of sampling a species with wood density ≥ 0.8 $g.cm^{-3}$ increases with species richness through the range of species richness values found in 0.04 ha subplots, but saturates by the species richness values found in 1 ha plots (Fig. S7). All but one 1 ha plot contains a species with wood density ≥ 0.8 $g.cm^{-3}$, however at 0.04 ha scale there is a positive relationship in all continents (Fig. S8).

Although the choice of 70 cm as a threshold for maximum diameter is supported by previous work demonstrating the contribution of trees of this size class to overall biomass [45], the thresholds chosen for both maximum diameter and wood density are essentially arbitrary. To explore sensitivity to this choice we also explored the effects using other, substantially different, thresholds. Setting a lower

threshold naturally means that the probability of sampling a high functioning species saturates at lower species richness, while setting a higher threshold means that it saturates at higher species richness (Fig. S9, Fig. S10). However, for all the thresholds which we investigated, the probability of sampling a high functioning species increased more rapidly with species richness though the range of species richness values found in 0.04 ha subplots than the range of species richness values found in 1 ha plots (Fig. S9, Fig. S10).

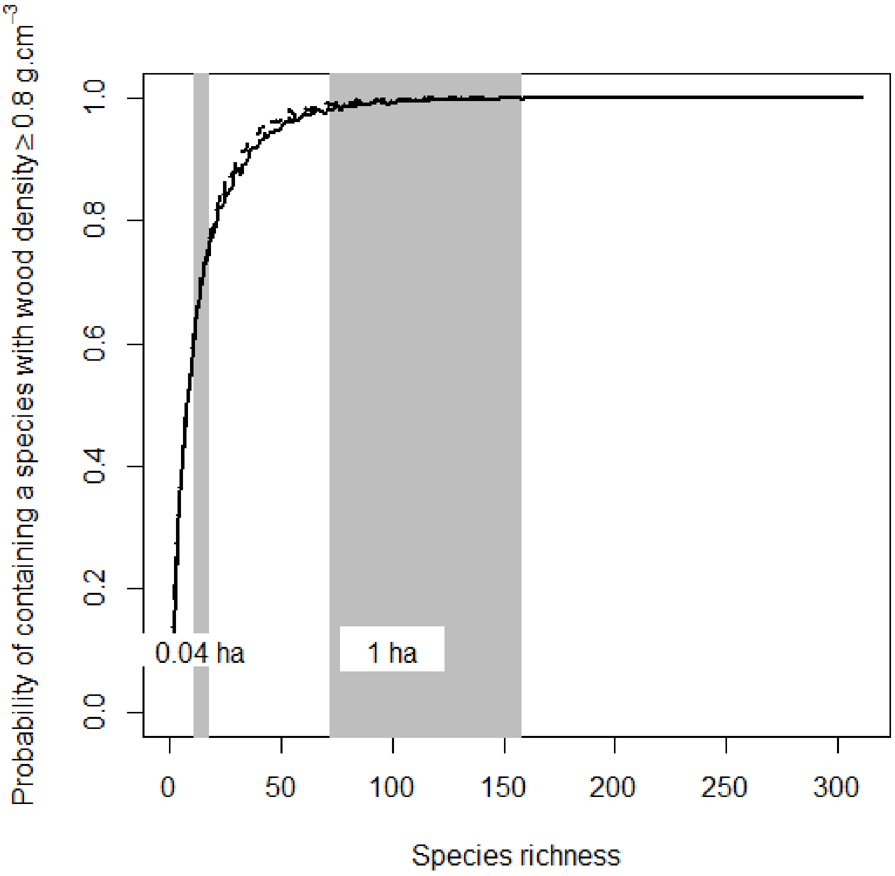

Supplementary Figure 7. Relationship between the species richness of a sample and the probability of that sample containing a species with high wood density (wood density ≥ 0.8 g.cm^{-3}). Probabilities were estimated by randomly sampling the species pool in each continent 1000 times for each continent and species richness increment, with the probability of selecting a species either equal for all species in a continent (solid line) or weighted by the proportion of plots that species was recorded in (dashed line). The interquartile range of species richness in 0.04 ha subplots and 1 ha plots are shown by grey shading.

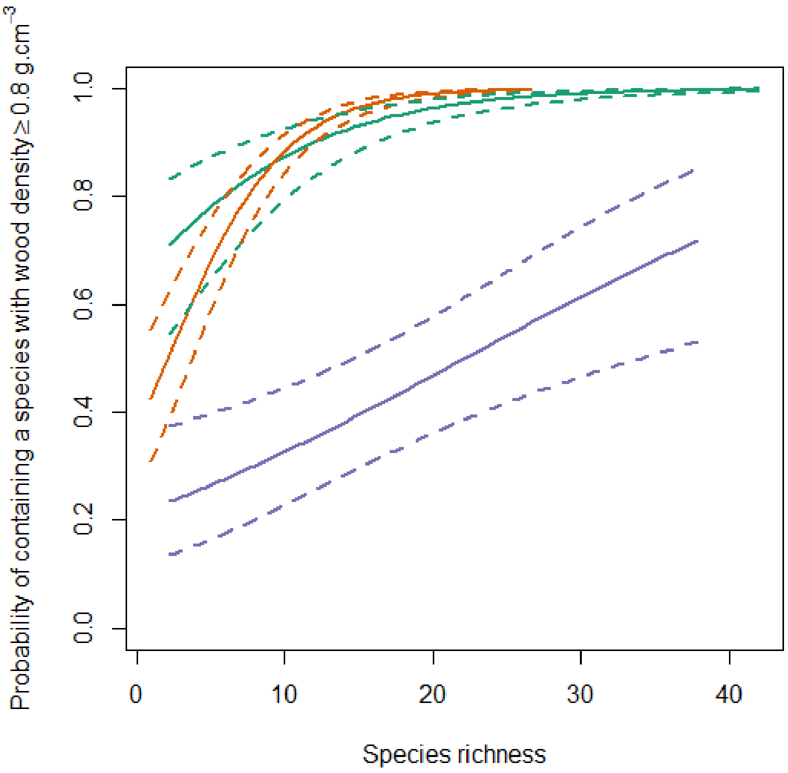

Supplementary Figure S8. Observed relationship between species richness of 0.04 ha subplots and the probability of that sample containing a species with high wood density (wood density ≥ 0.8 $g.cm^{-3}$). Fitted relationships in each continent are from generalised linear mixed effects models with binomial errors (green = South America, orange = Africa, purple = Asia). Standard errors are shown with dashed lines. Model coefficients are: South America, $\beta = 0.135$, $P < 0.001$; Africa, $\beta = 0.258$, $P < 0.001$; Asia, $\beta = 0.059$, $P = 0.001$. Relationships are not shown for 1 ha plots, as all but one of our 360 plots contained a species with high wood density.

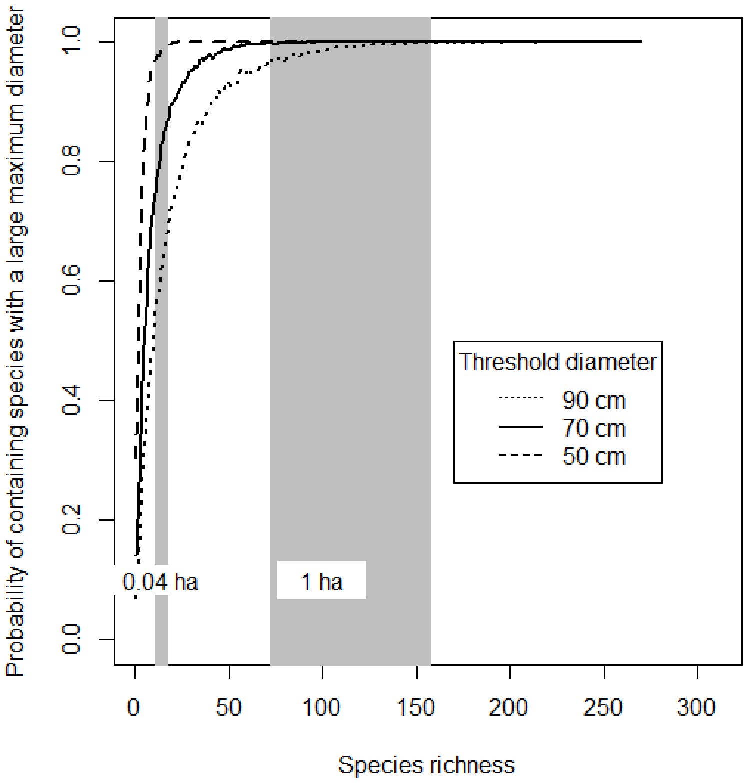

Supplementary Figure 9. Sensitivity of the relationship between the species richness of a sample and the probability of that sample containing a potentially large tree species to the choice of threshold maximum diameter. Probabilities were estimated by randomly sampling the species pool in each continent 1000 times for each continent and species richness increment, with the probability of selecting a species either equal for all species in a continent (solid line). The interquartile range of species richness in 0.04 ha subplots and 1 ha plots are shown by grey shading.

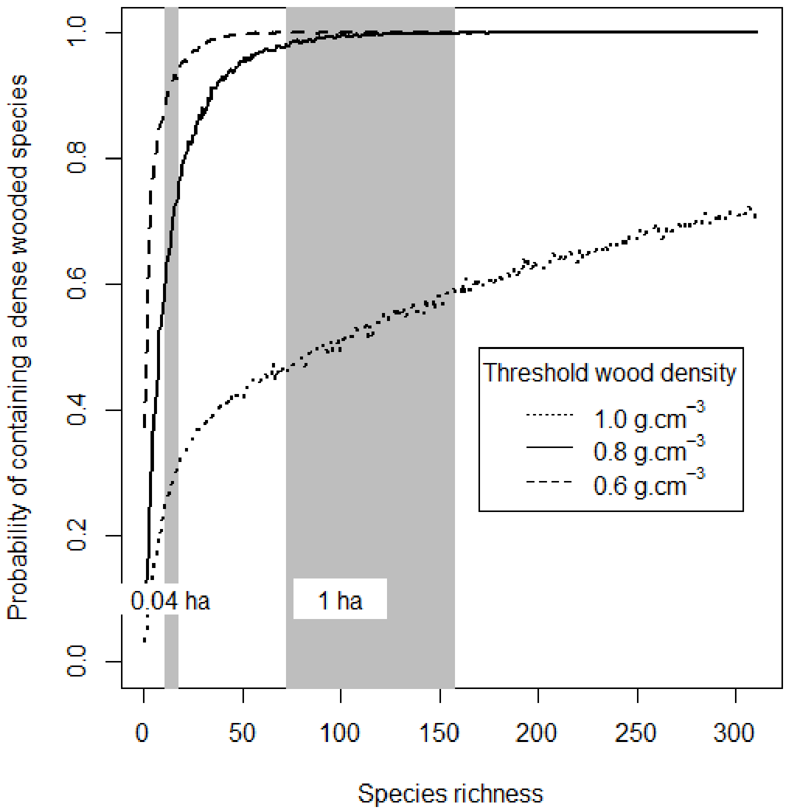

Supplementary Figure 10. Sensitivity of the relationship between the species richness of a sample and the probability of that sample containing a species with high wood density to the choice of threshold wood density. Probabilities were estimated by randomly sampling the species pool in each continent 1000 times for each continent and species richness increment, with the probability of selecting a species either equal for all species in a continent (solid line). The interquartile range of species richness in 0.04 ha subplots and 1 ha plots are shown by grey shading.

Conclusions

Our results are consistent with the weak positive relationship between diversity and carbon storage resulting from niche complementarity and/or selection effects, as at this scale we found a weak positive relationship between carbon storage and functional diversity (Fig. S1, consistent with niche complementarity) and between species richness and the probability of sampling a large tree (Fig. S6, consistent with selection effects). We note that positive diversity-carbon relationships at fine scales could also result from density dependent effects, which could arise if pests and pathogens incur a reduced cost on species with low local densities. We found no evidence of either selection effects or

niche complementarity operating at the 1-ha scale, which is consistent with both mechanisms being scale dependent. For selection effects this potential scale dependency could arise through the greater number of species as spatial scale increases, as we show that 1 ha plots are already sufficiently diverse for plots to be almost certain to contain a potentially large tree species (Fig. S5).

Carbon storage was related to the dominance of wood density and maximum diameter traits in species assemblages (Fig. S3), consistent with the biomass ratio hypothesis where ecosystem function is related to the traits of the dominant taxa. Our results are therefore consistent with previous studies in showing that carbon storage in 1 ha plots is related to functional dominance but not to functional diversity [29,30], and extend these by firstly showing that selection effects potentially saturate so are unlikely to explain functional dominance at 1 ha scales, and secondly by reporting correlations consistent with the operation of both niche complementarity and selection effects at the 0.04 ha scale.

Overall, we find support for the operation of niche complementarity and selection effects at 0.04 ha scale but no evidence for their operation at 1 ha scale, although as firm causal inferences cannot be drawn from correlative observational studies such as this substantial uncertainty remains about the role of niche complementarity and selection effects in tropical forests. The potential scale dependency of both mechanisms is consistent with the central finding of our pan-tropical analysis: except at the very smallest scales, across and within the three main tropical forest continents, above-ground live carbon storage and tree diversity are decoupled.

Additional figures and tables

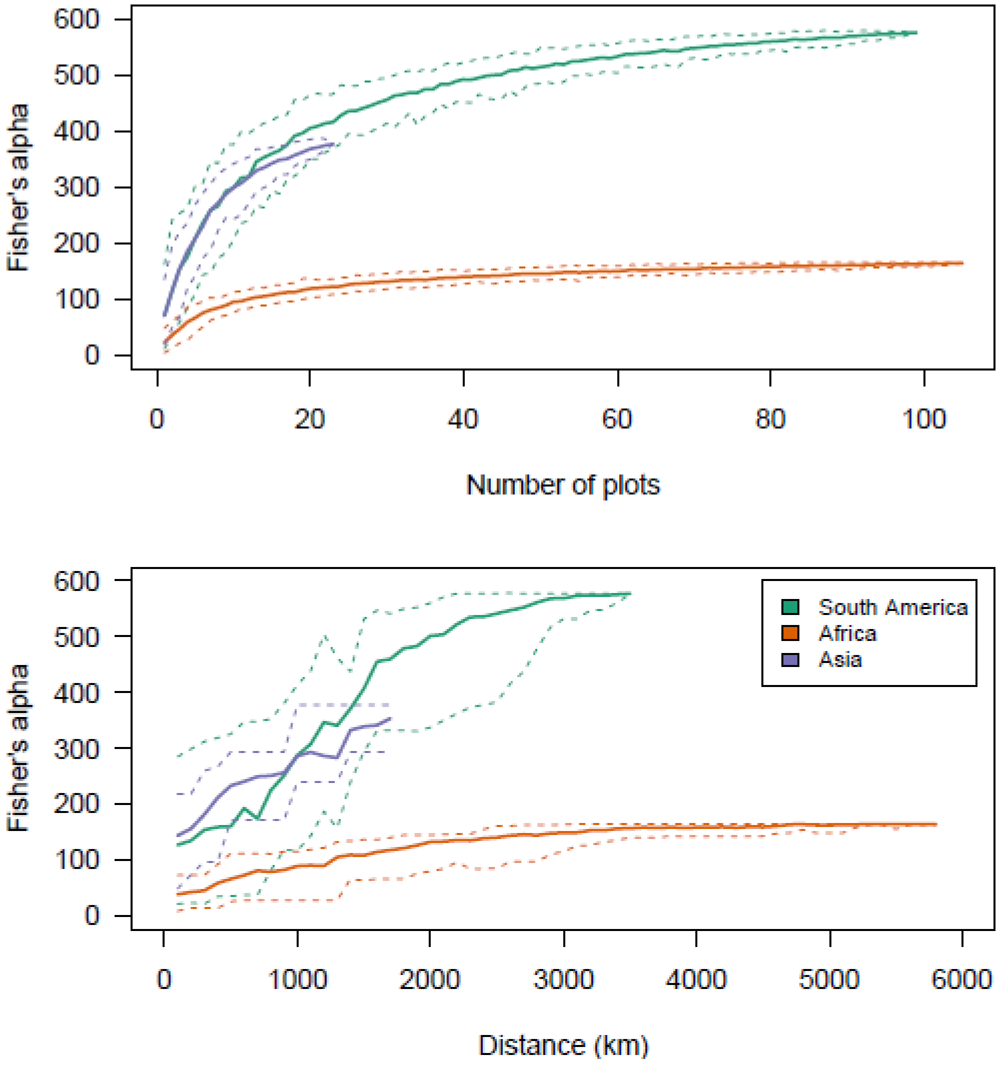

Supplementary Figure 11. Increase in diversity (Fisher's α) with increasing sample size of plots and increasing geographic distance around plots. For each sample size a random selection of *n* plots was drawn from the pool of available plots in each continent, while for each distance, a random plot was selected and all plots within a given distance of it were selected. This was repeated 100 times for each sample size and distance. Solid lines show mean values and dashed lines 95% confidence limits from these samples.

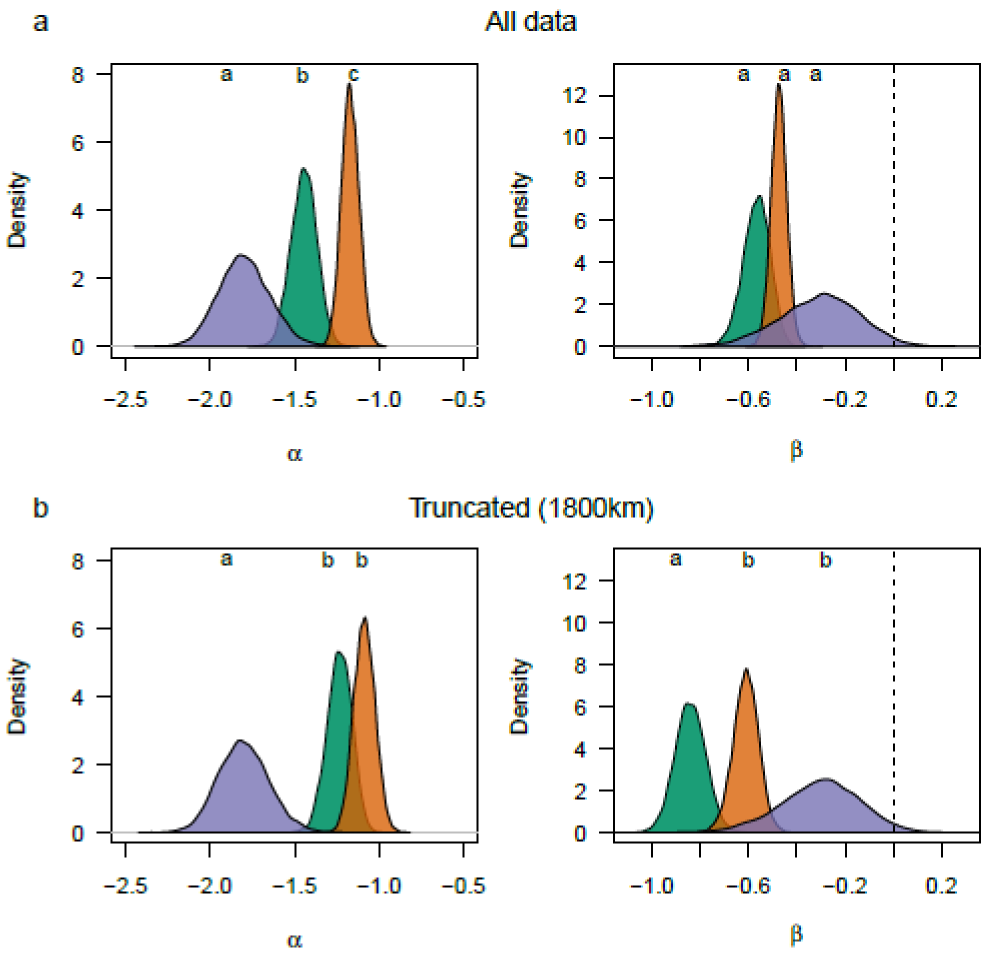

Supplementary Figure 12. Coefficients of generalised linear models of species similarity (Sørensen index) against distance, with α being the model intercept and β being the gradient. The distributions of parameters from 10000 bootstrap samples are shown. Models for South America are in green, Africa orange and Asia purple. Asia has a significantly lower intercept than Africa and South America, and when data were truncated a significantly shallower gradient than South America.

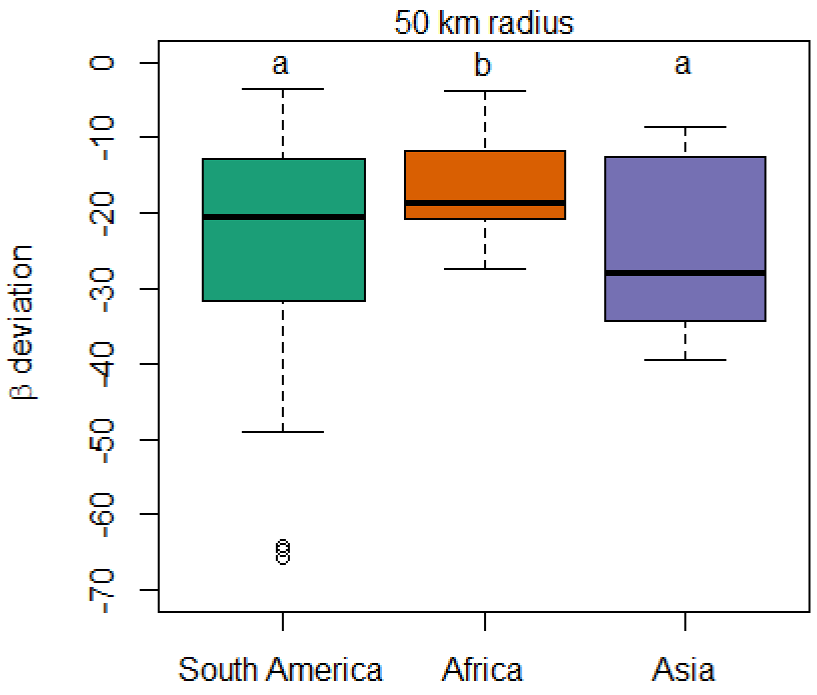

Supplementary Figure 13. Variation in the standardised effect size of the difference between observed and expected Sørensen index values (β deviation). β deviation values below zero indicate that forest stands are less similar than expected after controlling for the effect of gamma diversity. Expected values were generated by using a null model that randomly shuffles individual trees among plots within a sample area, while maintaining the number of stems in each plot and the overall gamma diversity and relative abundance of species in the sample area [47]. β deviation was estimated for each plot, with the sample area defined as a 50 km radius around that plot. The null model was run for 1000 iterations for each plot. Beta deviation differed significantly amongst continents (Kruskal-Wallis, $\chi^2 = 13.7$, $P = 0.001$). Different letters indicate significant differences between continents (pairwise Mann-Whitney tests with false discovery rate correction, $P < 0.05$). Beta deviation in all continents was significantly lower than zero (one sample Wilcoxon tests, $P < 0.001$).

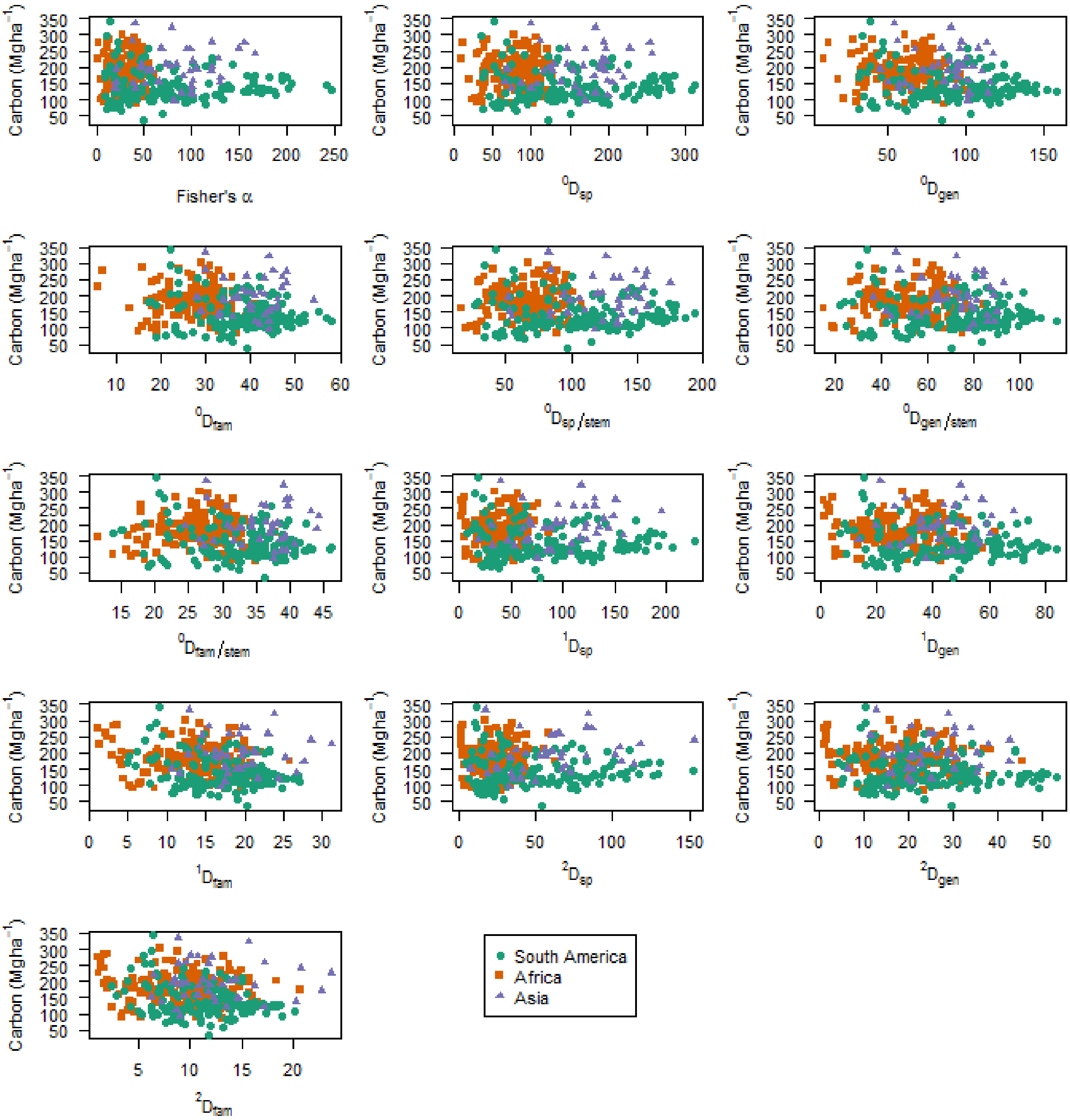

Supplementary Figure 14. Relationship between carbon and each diversity metric in plots in South America (green circles), Africa (orange squares and Asia (purple triangles). Diversity Hill numbers have been calculated for different taxonomic levels (sp= species, gen = genus, fam = family) and by area or per 300 stems (taxa/ stem). There are no significant relationships between any of these diversity metrics and forest biomass carbon.

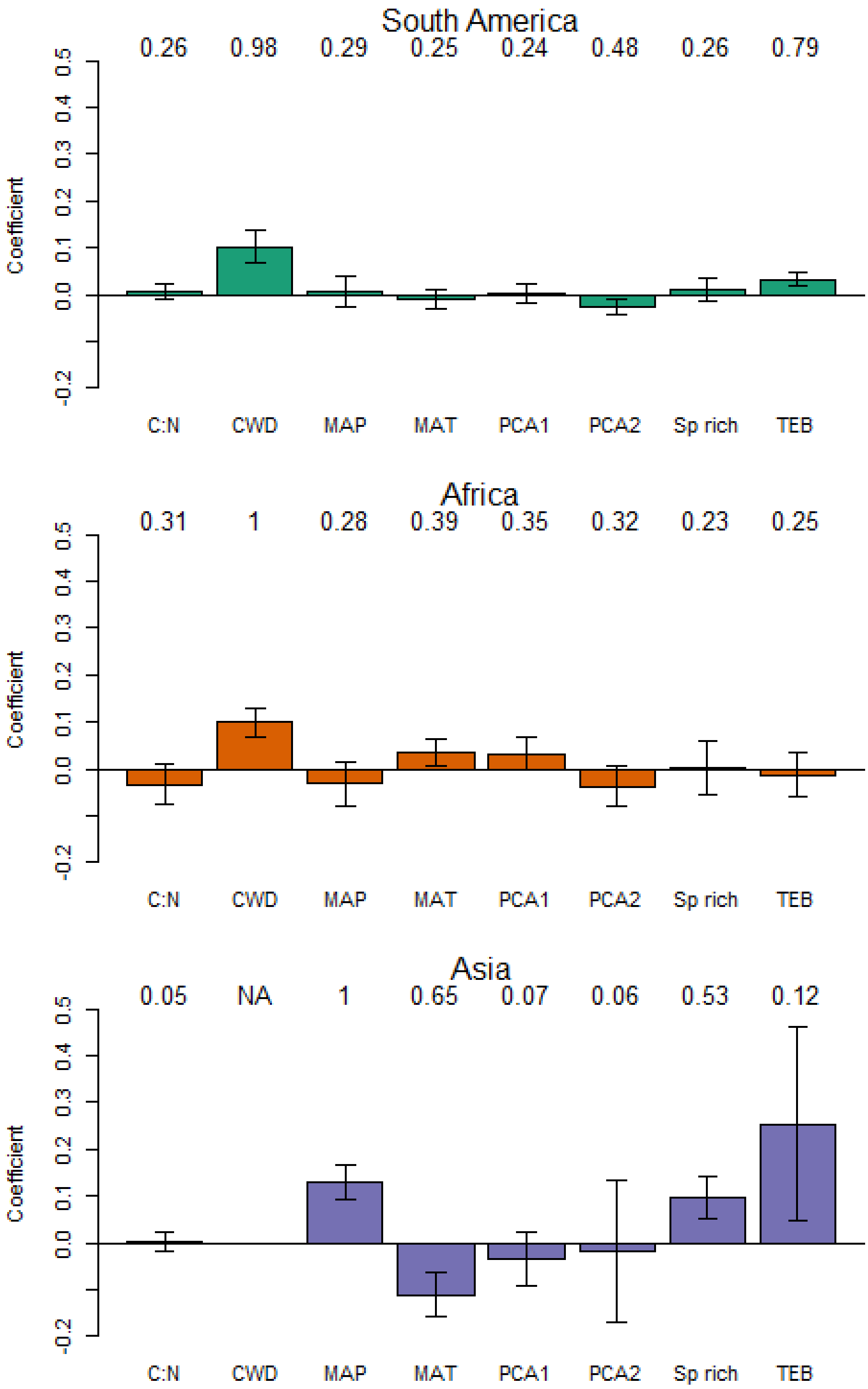

Supplementary Figure 15. Average model coefficients from simultaneous autoregressive error model of carbon storage in 1 ha plots as a function of species richness (Sp rich), climate (CWD, MAP, MAT) and soil (C:N, PCA1, PCA2, TEB). The sum of AIC_C weights of models containing a variable are shown above each variable. Coefficients of ordinary least squares models and models with other diversity metrics are presented in Table S4.

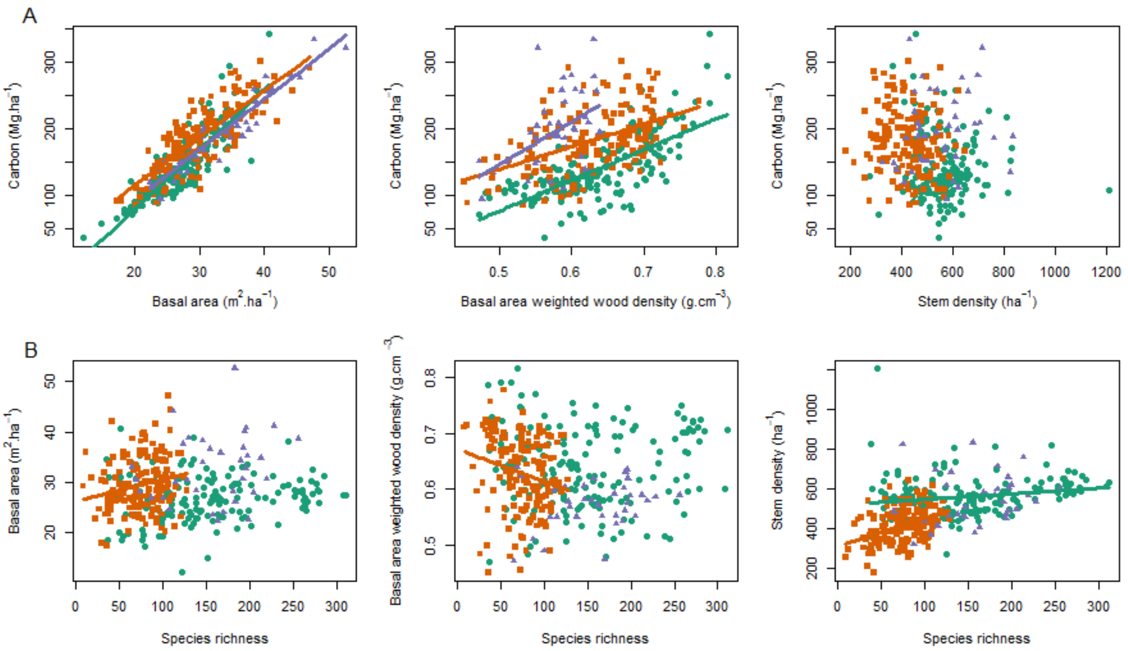

Supplementary Figure 16. (A) Relationship between carbon storage and stand structure and (B) relationship between stand structure and species richness in 1 ha plots. Data from South America are shown by green circles, Africa by orange squares, and Asia by purple triangles. Regression lines show significant relationships.

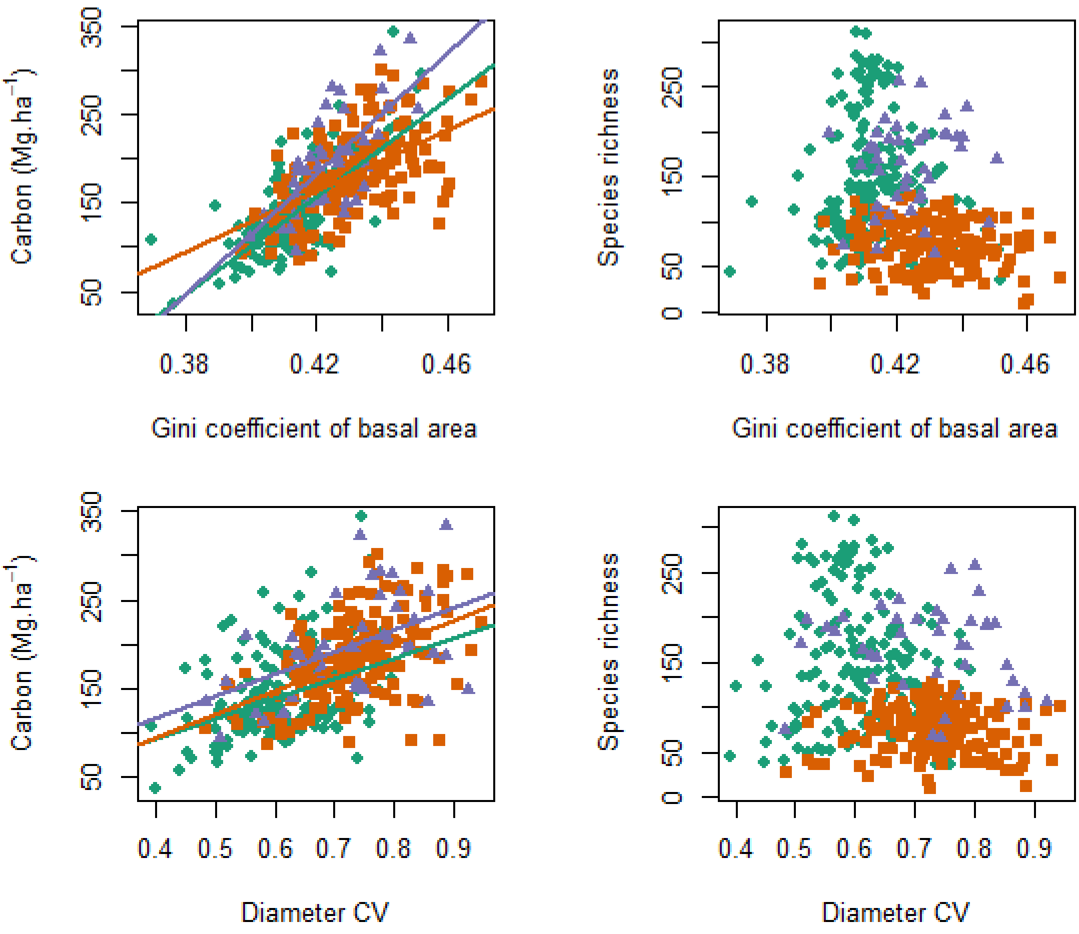

Supplementary Figure 17. Relationship between carbon storage, species richness and stem size inequality among 1 ha plots. Stem size inequality has been quantified as either the Gini coefficient of stem basal area or the coefficient of variation in stem diameters. For both metrics, higher values indicate greater inequality in stem size within a stand. In all continents, log-transformed carbon storage was positively related to both the Gini coefficient of basal area (South America: $\beta = 18.53$, $P < 0.001$; Africa: $\beta = 10.02$, $P < 0.001$; Asia: $\beta = 17.45$, $P < 0.001$) and the coefficient of variation in stem diameters (South America: $\beta = 1.74$, $P < 0.001$; Africa: $\beta = 1.49$, $P < 0.001$; Asia: $\beta = 1.37$, $P < 0.001$). There were no significant relationships between species richness and either metric of stem size inequality in any continent (negative-binomial GLM, $P \geq 0.078$).

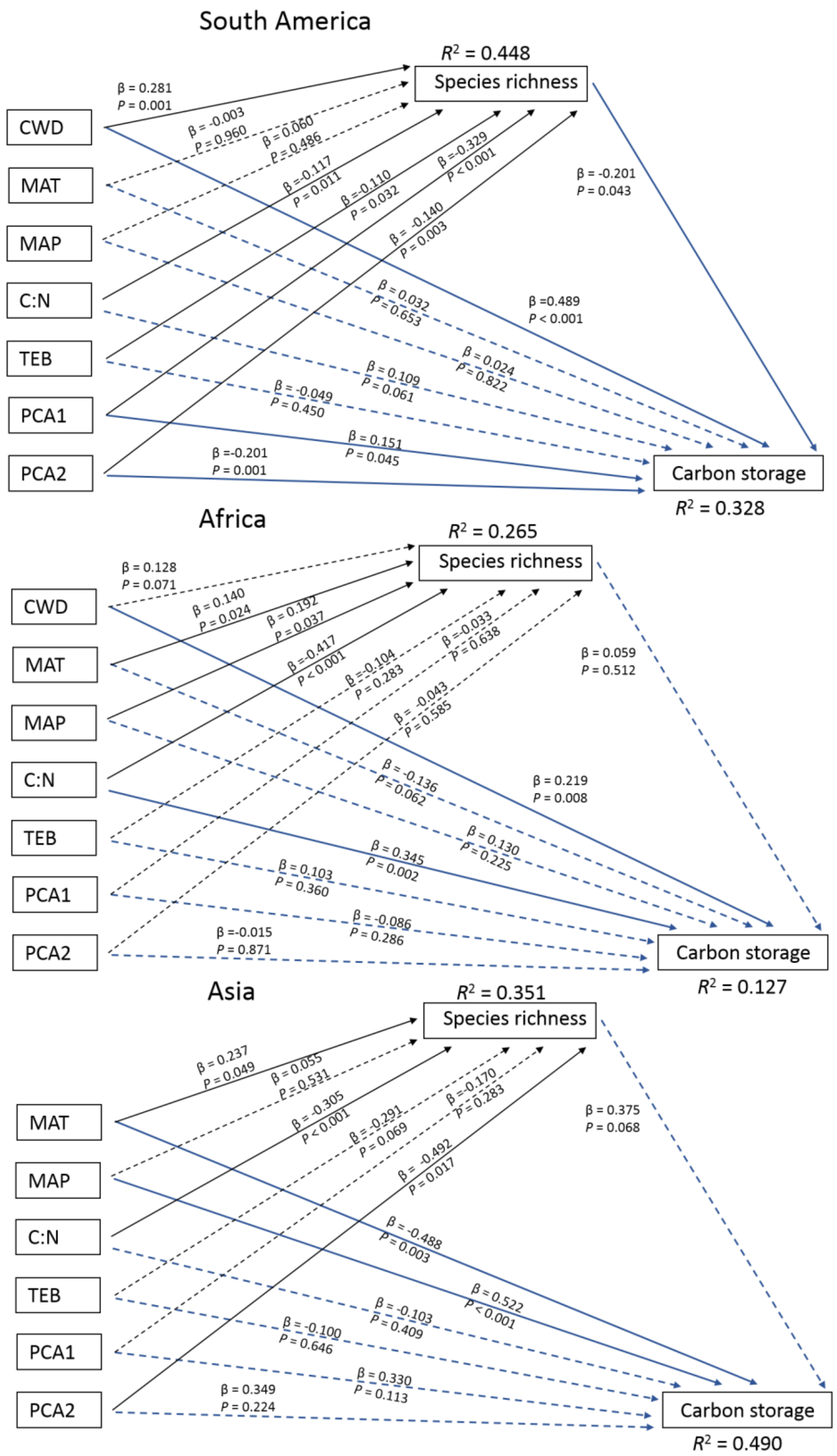

Supplementary Figure 18. Fitted structural equation models, where both species richness and carbon storage were modelled as a function of climate and soil, with carbon storage also a function of species richness. Models were parameterised separately for each continent, using the lavaan R package to define and parameterise paths. All variables were scaled and centred prior to analysis to have a mean of zero and standard deviation of one, with species richness and carbon storage also natural log transformed.

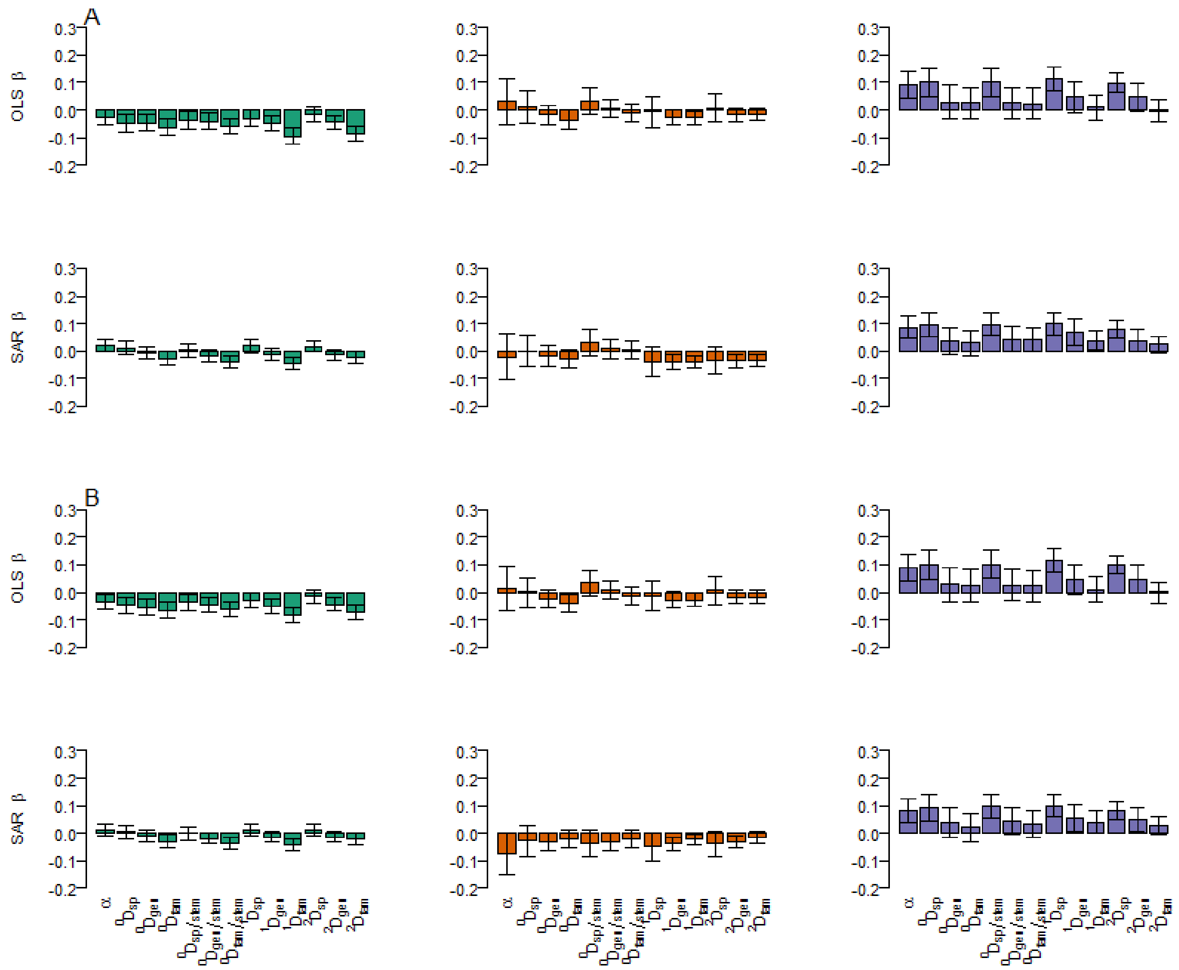

Supplementary Figure 19. Coefficients of relationships between diversity metrics and carbon between 1 ha plots within continents (green = South America, orange = Africa, purple = Asia) from multiple regression models also incorporating climate and edaphic variables. Results are shown for models run using (a) the best estimate soil class from the Harmonised World Soil Database and (b) the dominant soil class from the Harmonised World Soil Database. Diversity Hill numbers have been calculated for different taxonomic levels (sp= species, gen = genus, fam = family) and by area or per 300 stems (taxa/ stem).

Supplementary Table 1. Loadings of first two axes of principal components analysis performed on soil texture data from each 1 ha plot.

Soil texture class (%)	PC1	PC2
Sand	0.82	-0.07
Silt	-0.33	0.75
Clay	-0.47	-0.65
Proportion of variance explained	0.64	0.32

95.4% of variance was explained by first two axes.

Supplementary Table 2. Mean carbon storage and diversity in 1ha plots in South America, Africa and Asia. 95% confidence limits derived from 10000 bootstrap resamples of the data (sampling with replacement) are shown in parentheses. Different letters indicate significant differences between continents (ANOVA and subsequent Tukey's all-pair comparison, $P < 0.05$).

Variable	South America	Africa	Asia
Carbon (Mg. ha^{-1})	140 (133 – 148) ^{A}	183 (176 – 190) ^{B}	197 (180 - 215) ^{B}
^{0}D (species level)	152 (141 – 163) ^{B}	74 (70 – 78) ^{A}	162 (147 - 177) ^{C}
^{0}D (genus level)	91 (86 – 96) ^{C}	59 (56 – 62) ^{A}	87 (81 - 93) ^{B}
^{0}D (family level)	38 (37 – 39) ^{B}	28 (27 – 28) ^{A}	40 (38 - 42) ^{B}
^{1}D (species level)	85 (77 – 93) ^{B}	37 (34 – 40) ^{A}	98 (86 – 110) ^{B}
^{1}D (genus level)	43 (41 – 46) ^{B}	28 (26 – 30) ^{A}	43 (39 – 47) ^{B}
^{1}D (family level)	17 (17 – 18) ^{B}	13 (12 – 14) ^{A}	19 (18 – 21) ^{C}
^{2}D (species level)	48 (42 – 53) ^{B}	22 (20 – 24) ^{A}	60 (51 – 70) ^{C}
^{2}D (genus level)	25 (23 – 26) ^{B}	17 (16 – 18) ^{A}	24 (22 – 27) ^{B}
^{2}D (family level)	11 (11 – 12) ^{B}	9 (8 – 10) ^{A}	12 (11 – 14) ^{B}
Fisher's α	80 (71 – 88) ^{B}	28 (26 – 30) ^{A}	84 (73 - 96) ^{B}
^{0}D (species level) / 300 stems	109 (102 – 116) ^{B}	65 (62 – 69) ^{A}	120 (111 - 130) ^{B}
^{0}D (genus level) / 300 stems	72 (68 – 75) ^{B}	54 (51 – 56) ^{A}	71 (66 - 75) ^{B}
^{0}D (family level) / 300 stems	33 (32 – 34) ^{B}	26 (25 – 27) ^{A}	35 (34 - 37) ^{B}

Supplementary Table 3. Mean carbon storage and tree diversity in forest inventory plots in South America (n = 99), Africa (n = 105) and Asia (n = 23) where at least 90% of stems have been identified to species level. 95% confidence limits derived from 10000 bootstrap resamples of the data (sampling with replacement) are shown in parentheses. Different letters indicate significant differences between continents (ANOVA and subsequent Tukey's all-pair comparison, $P < 0.05$). Changing the species identification cut-off level for including plots has no impact on continental patterns of diversity and carbon (compare to Table 1 in main manuscript).

Variable	South America	Africa	Asia
Carbon (Mg. ha^{-1})	133 (125 – 142) ^{A}	177 (168 – 187) ^{B}	180 (159 - 202) ^{B}
Fisher's α	86 (74 – 99) ^{B}	26 (23 – 28) ^{A}	83 (74 - 99) ^{B}
Species richness (ha^{-1})	159 (144 – 174) ^{B}	71 (65 – 76) ^{A}	161 (139 - 183) ^{B}
(300 stems^{-1})	111 (102 – 120) ^{B}	63 (59 – 67) ^{A}	117 (104 - 130) ^{B}
Genus richness (ha^{-1})	93 (87 – 99) ^{B}	57 (53 – 61) ^{A}	89 (80 - 97) ^{B}
(300 stems^{-1})	73 (69 – 77) ^{B}	53 (49 – 56) ^{A}	72 (69 - 77) ^{B}
Family richness (ha^{-1})	39 (38 – 41) ^{B}	27 (25 – 28) ^{A}	41 (38 - 43) ^{B}
(300 stems^{-1})	34 (33 – 35) ^{B}	26 (25 – 27) ^{A}	36 (34 - 38) ^{B}

Supplementary Table 4. Kendall's tau correlations between carbon and diversity metrics in each continent. Both uncorrected *P* values and false-discovery rate corrected *P* values (*P*(fdr)) have been presented. Significant relationships prior to false-discovery rate correction are shown in italics. Power analysis was used to estimate the minimum effect size (presented as both τ and Pearson's *r*) detectable with 80% power.

Diversity metric	South America			Africa			Asia		
	τ	P	P (fdr)	τ	P	P (fdr)	τ	P	P (fdr)
^{0}D (species level)	0.084	0.12	0.223	0.014	0.788	0.999	0.132	0.230	0.598
^{0}D (genus level)	0.066	0.223	0.362	-0.016	0.765	0.999	-0.006	0.954	0.954
^{0}D (family level)	-0.007	0.893	0.956	-0.051	0.35	0.999	0.087	0.434	0.756
^{1}D (species level)	*0.107*	*0.046*	0.223	0	0.999	0.999	*0.218*	*0.048*	0.312
^{1}D (genus level)	0.034	0.521	0.616	-0.032	0.552	0.999	0.082	0.465	0.756
^{1}D (family level)	-0.089	0.096	0.223	-0.046	0.381	0.999	0.010	0.935	0.954
^{2}D (species level)	*0.12*	*0.025*	0.223	0	0.994	0.999	*0.246*	*0.025*	0.312
^{2}D (genus level)	-0.003	0.956	0.956	-0.031	0.559	0.999	0.159	0.153	0.566
^{2}D (family level)	-0.101	0.059	0.223	-0.033	0.529	0.999	-0.028	0.808	0.954
Fischer's α	0.083	0.12	0.223	0.012	0.821	0.999	0.115	0.302	0.654
^{0}D Species/ 300 stem	0.092	0.087	0.223	0.031	0.573	0.999	0.151	0.174	0.566
^{0}D Genus/ 300 stem	0.059	0.272	0.393	0.01	0.859	0.999	-0.051	0.652	0.942
^{0}D Family/ 300 stem	-0.042	0.43	0.559	-0.036	0.519	0.999	0.021	0.862	0.954
Detectable effect size	τ = 0.14<br>*r* = 0.22			τ = 0.14<br>*r* = 0.22			τ = 0.28<br>*r* = 0.43		

Supplementary Table 5 Averaged multiple regression model for ln(carbon) in 1 ha plots as a function of species richness, climate and soil variables. Both non-spatial ordinary least squares (OLS) and spatial simultaneous autoregressive error (SAR) models have been shown. $\sum\omega_i$ is the sum of AIC_C weights in models containing a variable, with values close to 1 indicating strong support for a variable.

	OLS					SAR				
Variable	β	SE	*Z*	*P*	$\sum\omega_i$	β	SE	*Z*	*P*	$\sum\omega_i$
	South America Fisher's α									
(Intercept)	4.931	0.028	176.801	0.000	NA	4.838	0.113	42.778	0.000	NA
C:N ratio	0.050	0.025	2.033	0.042	0.77	0.007	0.018	0.406	0.685	0.26
CWD	0.163	0.030	5.408	0.000	1.00	0.101	0.034	2.994	0.003	0.98
Fisher's α	-0.026	0.029	0.899	0.369	0.35	0.019	0.022	0.886	0.376	0.32
MAP	-0.018	0.034	0.522	0.602	0.27	0.005	0.035	0.150	0.881	0.29
MAT	0.030	0.025	1.187	0.235	0.41	-0.009	0.021	0.441	0.659	0.26
PCA1	0.086	0.026	3.330	0.001	1.00	0.004	0.021	0.175	0.861	0.24
PCA2	-0.041	0.019	2.146	0.032	0.81	-0.025	0.017	1.461	0.144	0.48
TEB	-0.009	0.021	0.423	0.672	0.27	0.034	0.015	2.194	0.028	0.80
	Africa Fisher's α									
(Intercept)	5.195	0.040	129.606	0.000	NA	5.182	0.063	82.417	0.000	NA
C:N ratio	-0.016	0.038	0.426	0.670	0.28	-0.036	0.043	0.818	0.414	0.31
CWD	0.062	0.028	2.194	0.028	0.81	0.099	0.031	3.200	0.001	1.00
Fisher's α	0.031	0.084	0.365	0.715	0.26	-0.022	0.083	0.260	0.795	0.24
MAP	0.065	0.047	1.365	0.172	0.51	-0.032	0.046	0.684	0.494	0.28
MAT	-0.031	0.026	1.194	0.232	0.41	0.034	0.028	1.200	0.230	0.39
PCA1	0.029	0.030	0.944	0.345	0.35	0.034	0.034	0.985	0.325	0.35
PCA2	-0.007	0.039	0.191	0.849	0.25	-0.038	0.043	0.880	0.379	0.32
TEB	-0.070	0.047	1.491	0.136	0.56	-0.011	0.046	0.247	0.805	0.25
	Asia Fisher's α									
(Intercept)	5.073	0.068	72.799	0.000	NA	5.113	0.125	41.019	0.000	NA
C:N ratio	0.015	0.024	0.598	0.550	0.07	0.002	0.020	0.089	0.929	0.06
Fisher's α	0.090	0.049	1.778	0.075	0.42	0.086	0.041	2.118	0.034	0.50
MAP	0.179	0.041	4.222	0.000	1.00	0.128	0.038	3.408	0.001	1.00
MAT	-0.118	0.055	2.083	0.037	0.64	-0.105	0.045	2.303	0.021	0.60
PCA1	-0.025	0.069	0.347	0.729	0.08	-0.032	0.056	0.564	0.573	0.07
PCA2	0.030	0.151	0.194	0.846	0.06	-0.017	0.151	0.110	0.912	0.07
TEB	0.197	0.237	0.804	0.422	0.10	0.258	0.207	1.247	0.212	0.14
	South America Species richness									
(Intercept)	4.936	0.029	169.527	0.000	NA	4.838	0.113	42.930	0.000	NA
C:N ratio	0.049	0.025	1.949	0.051	0.73	0.007	0.018	0.399	0.690	0.26
CWD	0.168	0.031	5.344	0.000	1.00	0.102	0.034	2.997	0.003	0.98
MAP	-0.018	0.034	0.522	0.602	0.26	0.006	0.034	0.188	0.851	0.29
MAT	0.030	0.025	1.185	0.236	0.40	-0.009	0.021	0.427	0.670	0.25
PCA1	0.080	0.027	2.918	0.004	0.97	0.002	0.020	0.116	0.908	0.24
PCA2	-0.044	0.020	2.233	0.026	0.85	-0.025	0.017	1.450	0.147	0.48
Species richness	-0.047	0.033	1.409	0.159	0.50	0.012	0.025	0.487	0.626	0.26
TEB	-0.011	0.021	0.517	0.605	0.28	0.033	0.015	2.177	0.029	0.79
	Africa Species richness									
(Intercept)	5.192	0.035	148.990	0.000	NA	5.185	0.060	85.967	0.000	NA
C:N ratio	-0.017	0.038	0.446	0.655	0.28	-0.034	0.043	0.796	0.426	0.31
CWD	0.062	0.028	2.211	0.027	0.81	0.099	0.031	3.195	0.001	1.00
MAP	0.064	0.047	1.357	0.175	0.50	-0.032	0.046	0.684	0.494	0.28
MAT	-0.031	0.026	1.190	0.234	0.41	0.034	0.028	1.192	0.233	0.39

PCA1	0.029	0.030	0.945	0.344	0.35	0.033	0.034	0.975	0.330	0.35
PCA2	-0.007	0.039	0.183	0.855	0.25	-0.038	0.043	0.890	0.373	0.32
Species richness	0.010	0.057	0.177	0.859	0.25	0.001	0.057	0.017	0.987	0.23
TEB	-0.069	0.047	1.482	0.138	0.55	-0.012	0.046	0.250	0.802	0.25
	Asia Species richness									
(Intercept)	5.069	0.069	71.481	0.000	NA	5.109	0.123	41.417	0.000	NA
C:N ratio	0.015	0.024	0.598	0.550	0.07	0.002	0.020	0.124	0.901	0.05
MAP	0.179	0.041	4.238	0.000	1.00	0.128	0.037	3.436	0.001	1.00
MAT	-0.122	0.055	2.135	0.033	0.67	-0.110	0.046	2.380	0.017	0.65
PCA1	-0.026	0.069	0.359	0.719	0.08	-0.033	0.057	0.585	0.558	0.07
PCA2	0.022	0.150	0.141	0.888	0.06	-0.017	0.151	0.114	0.909	0.06
Species richness	0.100	0.052	1.852	0.064	0.44	0.096	0.044	2.190	0.029	0.53
TEB	0.193	0.236	0.791	0.429	0.10	0.253	0.207	1.224	0.221	0.12
	South America Genus richness									
(Intercept)	4.937	0.029	169.730	0.000	NA	4.841	0.112	43.148	0.000	NA
C:N ratio	0.051	0.024	2.065	0.039	0.78	0.007	0.018	0.399	0.690	0.25
CWD	0.167	0.030	5.518	0.000	1.00	0.103	0.034	3.023	0.002	0.98
Genus richness	-0.046	0.030	1.501	0.133	0.53	-0.007	0.022	0.324	0.746	0.25
MAP	-0.016	0.034	0.464	0.642	0.26	0.008	0.034	0.243	0.808	0.29
MAT	0.028	0.025	1.098	0.272	0.38	-0.010	0.021	0.450	0.653	0.26
PCA1	0.080	0.027	2.932	0.003	0.98	0.000	0.020	0.006	0.995	0.24
PCA2	-0.041	0.019	2.178	0.029	0.82	-0.025	0.017	1.440	0.150	0.48
TEB	-0.010	0.021	0.491	0.624	0.27	0.033	0.015	2.159	0.031	0.78
	Africa Genus richness									
(Intercept)	5.188	0.031	164.165	0.000	NA	5.182	0.059	88.360	0.000	NA
C:N ratio	-0.019	0.038	0.496	0.620	0.29	-0.037	0.044	0.838	0.402	0.32
CWD	0.063	0.028	2.252	0.024	0.82	0.099	0.031	3.207	0.001	1.00
Genus richness	-0.017	0.035	0.475	0.635	0.27	-0.018	0.036	0.503	0.615	0.26
MAP	0.064	0.047	1.346	0.178	0.50	-0.031	0.046	0.679	0.497	0.28
MAT	-0.030	0.026	1.180	0.238	0.41	0.034	0.028	1.200	0.230	0.39
PCA1	0.028	0.030	0.929	0.353	0.34	0.034	0.034	0.995	0.320	0.35
PCA2	-0.006	0.039	0.147	0.883	0.25	-0.037	0.043	0.868	0.385	0.31
TEB	-0.068	0.047	1.448	0.148	0.54	-0.011	0.046	0.238	0.812	0.25
	Asia Genus richness									
(Intercept)	5.092	0.064	77.681	0.000	NA	5.222	0.145	36.096	0.000	NA
C:N ratio	0.014	0.024	0.578	0.563	0.12	-0.010	0.021	0.464	0.642	0.08
Genus richness	0.028	0.061	0.452	0.651	0.10	0.037	0.048	0.785	0.432	0.09
MAP	0.176	0.041	4.119	0.000	1.00	0.081	0.036	2.280	0.023	0.51
MAT	-0.111	0.055	1.953	0.051	0.61	-0.104	0.041	2.537	0.011	0.71
PCA1	-0.019	0.069	0.272	0.785	0.09	-0.108	0.082	1.324	0.186	0.23
PCA2	0.010	0.163	0.058	0.954	0.10	-0.355	0.249	1.424	0.154	0.28
TEB	0.189	0.246	0.745	0.456	0.14	0.561	0.339	1.652	0.099	0.40
	South America Family richness									
(Intercept)	4.948	0.030	163.808	0.000	NA	4.845	0.112	43.193	0.000	NA
C:N ratio	0.050	0.024	2.062	0.039	0.78	0.007	0.018	0.395	0.693	0.25
CWD	0.168	0.028	5.925	0.000	1.00	0.103	0.034	3.009	0.003	0.98
Family richness	-0.064	0.030	2.133	0.033	0.80	-0.026	0.022	1.178	0.239	0.40
MAP	-0.010	0.034	0.292	0.770	0.25	0.011	0.034	0.332	0.740	0.30
MAT	0.022	0.025	0.864	0.387	0.32	-0.012	0.022	0.554	0.579	0.27

PCA1	0.073	0.026	2.757	0.006	0.97	-0.003	0.021	0.160	0.873	0.25
PCA2	-0.041	0.018	2.184	0.029	0.82	-0.025	0.017	1.435	0.151	0.47
TEB	-0.011	0.020	0.520	0.603	0.27	0.033	0.015	2.153	0.031	0.77
	Africa Family richness									
(Intercept)	5.182	0.033	154.128	0.000	NA	5.179	0.059	87.131	0.000	NA
C:N ratio	-0.020	0.038	0.537	0.591	0.29	-0.038	0.044	0.870	0.384	0.32
CWD	0.065	0.028	2.310	0.021	0.84	0.100	0.031	3.221	0.001	1.00
Family richness	-0.036	0.034	1.042	0.297	0.36	-0.027	0.033	0.816	0.414	0.31
MAP	0.063	0.047	1.343	0.179	0.50	-0.031	0.046	0.660	0.509	0.28
MAT	-0.030	0.026	1.162	0.245	0.40	0.033	0.028	1.180	0.238	0.38
PCA1	0.027	0.030	0.891	0.373	0.33	0.035	0.034	1.010	0.313	0.36
PCA2	-0.004	0.039	0.092	0.927	0.25	-0.036	0.043	0.844	0.399	0.31
TEB	-0.066	0.048	1.383	0.167	0.51	-0.011	0.046	0.228	0.820	0.24
	Asia Family richness									
(Intercept)	5.092	0.064	76.789	0.000	NA	5.222	0.145	36.041	0.000	NA
C:N ratio	0.014	0.024	0.578	0.563	0.12	-0.010	0.021	0.478	0.633	0.09
Family richness	0.024	0.059	0.399	0.690	0.10	0.030	0.045	0.673	0.501	0.08
MAP	0.176	0.041	4.124	0.000	1.00	0.081	0.036	2.261	0.024	0.50
MAT	-0.111	0.055	1.950	0.051	0.61	-0.103	0.041	2.532	0.011	0.71
PCA1	-0.019	0.069	0.272	0.785	0.09	-0.108	0.082	1.320	0.187	0.23
PCA2	0.010	0.163	0.058	0.954	0.10	-0.355	0.249	1.424	0.155	0.28
TEB	0.189	0.246	0.745	0.456	0.14	0.560	0.340	1.648	0.099	0.40
	South America Species per 300 stems									
(Intercept)	4.933	0.028	174.606	0.000	NA	4.845	0.109	44.318	0.000	NA
C:N ratio	0.049	0.025	1.997	0.046	0.74	0.007	0.018	0.391	0.696	0.26
CWD	0.165	0.031	5.324	0.000	1.00	0.097	0.033	2.916	0.004	0.97
MAP	-0.015	0.039	0.370	0.711	0.26	0.005	0.035	0.129	0.897	0.27
MAT	0.030	0.025	1.199	0.231	0.42	-0.012	0.022	0.554	0.580	0.27
PCA1	0.083	0.026	3.123	0.002	1.00	0.002	0.020	0.076	0.940	0.24
PCA2	-0.043	0.019	2.194	0.028	0.83	-0.023	0.017	1.321	0.187	0.44
Species per 300 stems	-0.038	0.031	1.202	0.229	0.42	0.004	0.024	0.154	0.877	0.24
TEB	-0.007	0.024	0.286	0.775	0.26	0.030	0.017	1.788	0.074	0.62
	Africa Species per 300 stems									
(Intercept)	5.206	0.037	138.288	0.000	NA	5.197	0.066	79.307	0.000	NA
C:N ratio	-0.051	0.040	1.264	0.206	0.43	-0.043	0.045	0.962	0.336	0.34
CWD	0.010	0.043	0.225	0.822	0.31	0.076	0.037	2.076	0.038	0.76
MAP	0.092	0.043	2.098	0.036	0.78	-0.030	0.050	0.590	0.555	0.27
MAT	-0.009	0.028	0.320	0.749	0.26	0.048	0.030	1.585	0.113	0.52
PCA1	0.029	0.033	0.885	0.376	0.34	0.028	0.034	0.834	0.404	0.31
PCA2	0.020	0.043	0.462	0.644	0.27	-0.018	0.044	0.406	0.685	0.25
Species per 300 stems	0.033	0.047	0.690	0.490	0.30	0.033	0.048	0.690	0.490	0.28
TEB	-0.103	0.044	2.332	0.020	0.90	-0.014	0.055	0.256	0.798	0.26
	Asia Species per 300 stems									
(Intercept)	5.054	0.076	64.759	0.000	NA	5.093	0.127	40.034	0.000	NA
C:N ratio	0.015	0.023	0.622	0.534	0.07	0.003	0.020	0.130	0.896	0.05
MAP	0.181	0.041	4.282	0.000	1.00	0.130	0.037	3.486	0.000	1.00
MAT	-0.117	0.054	2.103	0.035	0.63	-0.104	0.044	2.346	0.019	0.60
PCA1	-0.027	0.069	0.381	0.703	0.08	-0.035	0.056	0.624	0.533	0.07
PCA2	0.037	0.150	0.237	0.813	0.04	0.014	0.127	0.113	0.910	0.05
Species per 300 stems	0.101	0.050	1.941	0.052	0.53	0.097	0.042	2.333	0.020	0.60

TEB	0.201	0.236	0.823	0.411	0.10	0.253	0.194	1.305	0.192	0.12
	South America Genera per 300 stems									
(Intercept)	4.933	0.028	176.185	0.000	NA	4.846	0.109	44.414	0.000	NA
C:N ratio	0.051	0.024	2.058	0.040	0.78	0.007	0.018	0.398	0.690	0.25
CWD	0.165	0.030	5.473	0.000	1.00	0.098	0.033	2.943	0.003	0.97
Genera per 300 stems	-0.041	0.028	1.447	0.148	0.50	-0.016	0.021	0.786	0.432	0.30
MAP	-0.012	0.039	0.311	0.756	0.25	0.006	0.035	0.175	0.861	0.27
MAT	0.028	0.025	1.123	0.261	0.39	-0.013	0.022	0.591	0.554	0.27
PCA1	0.081	0.027	3.003	0.003	0.99	-0.001	0.021	0.067	0.946	0.24
PCA2	-0.041	0.019	2.173	0.030	0.82	-0.023	0.017	1.311	0.190	0.44
TEB	-0.007	0.024	0.292	0.770	0.26	0.029	0.017	1.765	0.078	0.61
	Africa Genera per 300 stems									
(Intercept)	5.202	0.034	152.373	0.000	NA	5.192	0.064	81.491	0.000	NA
C:N ratio	-0.053	0.040	1.315	0.189	0.45	-0.045	0.045	0.996	0.319	0.35
CWD	0.010	0.043	0.236	0.813	0.31	0.076	0.037	2.078	0.038	0.77
Genera per 300 stems	0.008	0.032	0.239	0.811	0.25	0.008	0.033	0.237	0.813	0.24
MAP	0.093	0.043	2.127	0.033	0.78	-0.029	0.050	0.572	0.568	0.27
MAT	-0.008	0.028	0.270	0.787	0.26	0.048	0.030	1.606	0.108	0.53
PCA1	0.029	0.033	0.892	0.372	0.34	0.029	0.034	0.856	0.392	0.32
PCA2	0.022	0.043	0.503	0.615	0.28	-0.016	0.044	0.379	0.705	0.24
TEB	-0.104	0.044	2.346	0.019	0.91	-0.014	0.055	0.257	0.797	0.26
	Asia Genera per 300 stems									
(Intercept)	5.092	0.064	77.413	0.000	NA	5.221	0.145	36.130	0.000	NA
C:N ratio	0.015	0.024	0.598	0.550	0.11	-0.010	0.021	0.490	0.624	0.09
Genera per 300 stems	0.025	0.057	0.427	0.669	0.11	0.044	0.046	0.966	0.334	0.10
MAP	0.176	0.041	4.116	0.000	1.00	0.081	0.035	2.282	0.023	0.51
MAT	-0.111	0.055	1.950	0.051	0.61	-0.104	0.041	2.540	0.011	0.71
PCA1	-0.019	0.069	0.272	0.785	0.09	-0.108	0.082	1.322	0.186	0.22
PCA2	0.010	0.163	0.058	0.954	0.10	-0.355	0.249	1.425	0.154	0.27
TEB	0.189	0.246	0.746	0.456	0.14	0.560	0.339	1.650	0.099	0.39
	South America Families per 300 stems									
(Intercept)	4.940	0.028	175.164	0.000	NA	4.851	0.110	44.195	0.000	NA
C:N ratio	0.050	0.024	2.062	0.039	0.77	0.007	0.018	0.396	0.692	0.25
CWD	0.163	0.028	5.734	0.000	1.00	0.096	0.034	2.850	0.004	0.95
Families per 300 stems	-0.060	0.027	2.182	0.029	0.81	-0.038	0.021	1.798	0.072	0.65
MAP	-0.002	0.040	0.057	0.955	0.24	0.018	0.037	0.497	0.620	0.31
MAT	0.023	0.025	0.913	0.362	0.34	-0.018	0.022	0.812	0.417	0.31
PCA1	0.072	0.026	2.713	0.007	0.97	-0.011	0.023	0.488	0.625	0.28
PCA2	-0.041	0.019	2.192	0.028	0.82	-0.023	0.017	1.307	0.191	0.43
TEB	-0.006	0.023	0.244	0.807	0.25	0.029	0.017	1.759	0.079	0.61
	Africa Families per 300 stems									
(Intercept)	5.199	0.034	151.668	0.000	NA	5.192	0.064	81.181	0.000	NA
C:N ratio	-0.055	0.040	1.362	0.173	0.47	-0.045	0.045	1.001	0.317	0.35
CWD	0.011	0.043	0.251	0.802	0.31	0.076	0.037	2.083	0.037	0.77
Families per 300 stems	-0.010	0.033	0.304	0.761	0.25	0.004	0.033	0.137	0.891	0.23
MAP	0.093	0.043	2.136	0.033	0.79	-0.029	0.050	0.578	0.563	0.27
MAT	-0.006	0.028	0.223	0.824	0.25	0.048	0.030	1.614	0.106	0.54
PCA1	0.029	0.033	0.890	0.373	0.34	0.029	0.034	0.862	0.389	0.32
PCA2	0.023	0.043	0.539	0.590	0.28	-0.016	0.044	0.375	0.708	0.25
TEB	-0.105	0.044	2.355	0.019	0.91	-0.014	0.056	0.254	0.800	0.26

	Asia Families per 300 stems									
(Intercept)	5.092	0.065	76.189	0.000	NA	5.219	0.145	35.990	0.000	NA
C:N ratio	0.015	0.024	0.598	0.550	0.11	-0.009	0.020	0.449	0.654	0.08
Families per 300 stems	0.023	0.056	0.394	0.694	0.10	0.044	0.043	1.028	0.304	0.13
MAP	0.176	0.041	4.123	0.000	1.00	0.080	0.036	2.264	0.024	0.48
MAT	-0.110	0.055	1.948	0.051	0.60	-0.103	0.041	2.532	0.011	0.71
PCA1	-0.019	0.069	0.272	0.785	0.09	-0.107	0.081	1.315	0.189	0.23
PCA2	0.010	0.163	0.058	0.954	0.10	-0.360	0.248	1.449	0.147	0.26
TEB	0.189	0.246	0.746	0.456	0.14	0.556	0.339	1.638	0.101	0.39
	South America Shannon index_sp									
(Intercept)	4.932	0.028	176.548	0.000	NA	4.838	0.113	42.840	0.000	NA
C:N ratio	0.050	0.025	2.017	0.044	0.76	0.007	0.018	0.409	0.682	0.26
CWD	0.165	0.030	5.372	0.000	1.00	0.102	0.034	3.001	0.003	0.98
Shannon index_sp	-0.030	0.029	1.030	0.303	0.38	0.018	0.023	0.786	0.432	0.30
MAP	-0.018	0.034	0.534	0.593	0.27	0.006	0.034	0.172	0.864	0.29
MAT	0.029	0.025	1.167	0.243	0.40	-0.009	0.021	0.420	0.674	0.25
PCA1	0.085	0.025	3.317	0.001	1.00	0.003	0.021	0.154	0.878	0.24
PCA2	-0.042	0.019	2.163	0.031	0.83	-0.025	0.017	1.446	0.148	0.48
TEB	-0.009	0.021	0.427	0.669	0.26	0.033	0.015	2.190	0.029	0.80
	Africa Shannon index_sp									
(Intercept)	5.189	0.033	154.343	0.000	NA	5.179	0.061	84.831	0.000	NA
C:N ratio	-0.018	0.038	0.467	0.640	0.28	-0.037	0.044	0.850	0.395	0.32
CWD	0.063	0.028	2.230	0.026	0.82	0.099	0.031	3.207	0.001	1.00
Shannon index_sp	-0.007	0.055	0.118	0.906	0.24	-0.037	0.053	0.700	0.484	0.29
MAP	0.064	0.047	1.355	0.175	0.51	-0.031	0.046	0.664	0.507	0.28
MAT	-0.031	0.026	1.188	0.235	0.41	0.034	0.028	1.202	0.229	0.39
PCA1	0.029	0.030	0.946	0.344	0.35	0.035	0.035	1.017	0.309	0.36
PCA2	-0.007	0.039	0.168	0.866	0.25	-0.037	0.043	0.851	0.395	0.31
TEB	-0.069	0.047	1.471	0.141	0.55	-0.011	0.046	0.243	0.808	0.25
	Asia Shannon index_sp									
(Intercept)	5.038	0.067	72.796	0.000	NA	5.169	0.129	40.203	0.000	NA
C:N ratio	0.013	0.023	0.567	0.571	0.03	-0.007	0.020	0.375	0.707	0.03
Shannon index_sp	0.114	0.044	2.528	0.011	0.83	0.099	0.039	2.525	0.012	0.64
MAP	0.183	0.039	4.524	0.000	1.00	0.089	0.033	2.710	0.007	0.64
MAT	-0.132	0.052	2.433	0.015	0.76	-0.117	0.040	2.921	0.003	0.84
PCA1	-0.039	0.070	0.541	0.589	0.04	-0.102	0.078	1.299	0.194	0.12
PCA2	0.055	0.150	0.355	0.723	0.03	-0.351	0.257	1.365	0.172	0.11
TEB	0.228	0.231	0.956	0.339	0.05	0.504	0.322	1.567	0.117	0.22
	South America Shannon index_gen									
(Intercept)	4.937	0.028	175.385	0.000	NA	4.841	0.112	43.232	0.000	NA
C:N ratio	0.050	0.024	2.068	0.039	0.78	0.007	0.018	0.395	0.693	0.26
CWD	0.170	0.029	5.718	0.000	1.00	0.102	0.034	3.021	0.003	0.98
Shannon index_gen	-0.049	0.026	1.835	0.067	0.66	-0.012	0.020	0.627	0.531	0.28
MAP	-0.013	0.034	0.392	0.695	0.26	0.009	0.034	0.249	0.803	0.29
MAT	0.024	0.025	0.961	0.337	0.34	-0.010	0.022	0.478	0.633	0.26
PCA1	0.078	0.026	2.930	0.003	0.99	0.000	0.020	0.020	0.984	0.24
PCA2	-0.041	0.019	2.189	0.029	0.83	-0.025	0.017	1.442	0.149	0.48
TEB	-0.008	0.021	0.390	0.697	0.26	0.033	0.015	2.160	0.031	0.78
	Africa Shannon index_gen									
(Intercept)	5.186	0.031	165.462	0.000	NA	5.174	0.060	86.411	0.000	NA

C:N ratio	-0.021	0.038	0.538	0.590	0.29	-0.042	0.044	0.952	0.341	0.35
CWD	0.063	0.028	2.264	0.024	0.83	0.100	0.031	3.238	0.001	1.00
Shannon index_gen	-0.027	0.029	0.933	0.351	0.34	-0.040	0.028	1.437	0.151	0.49
MAP	0.063	0.047	1.341	0.180	0.50	-0.031	0.046	0.665	0.506	0.27
MAT	-0.030	0.026	1.169	0.242	0.40	0.035	0.028	1.233	0.218	0.40
PCA1	0.029	0.030	0.944	0.345	0.35	0.039	0.035	1.117	0.264	0.38
PCA2	-0.004	0.039	0.095	0.925	0.25	-0.033	0.044	0.751	0.453	0.30
TEB	-0.068	0.047	1.447	0.148	0.54	-0.012	0.046	0.250	0.803	0.24
	Asia Shannon index_gen									
(Intercept)	5.092	0.063	77.850	0.000	NA	5.236	0.083	62.925	0.000	NA
C:N ratio	0.015	0.024	0.598	0.550	0.11	-0.023	0.020	1.145	0.252	0.20
Shannon index_gen	0.046	0.055	0.805	0.421	0.13	0.070	0.048	1.478	0.139	0.29
MAP	0.176	0.041	4.129	0.000	1.00	0.093	0.047	1.966	0.049	0.41
MAT	-0.111	0.055	1.960	0.050	0.61	-0.104	0.050	2.062	0.039	0.58
PCA1	-0.019	0.069	0.272	0.785	0.09	-0.058	0.088	0.665	0.506	0.15
PCA2	0.010	0.163	0.058	0.954	0.09	-0.161	0.208	0.773	0.440	0.17
TEB	0.189	0.245	0.744	0.457	0.13	0.293	0.315	0.930	0.352	0.18
	South America Shannon index_fam									
(Intercept)	4.944	0.027	184.020	0.000	NA	4.847	0.110	44.008	0.000	NA
C:N ratio	0.038	0.024	1.579	0.114	0.56	0.004	0.018	0.241	0.809	0.24
CWD	0.166	0.026	6.221	0.000	1.00	0.097	0.034	2.824	0.005	0.95
Shannon index_fam	-0.095	0.028	3.319	0.001	1.00	-0.042	0.022	1.933	0.053	0.71
MAP	-0.002	0.034	0.048	0.961	0.24	0.021	0.036	0.585	0.559	0.33
MAT	0.008	0.025	0.304	0.761	0.24	-0.021	0.023	0.926	0.355	0.34
PCA1	0.059	0.024	2.373	0.018	0.89	-0.010	0.022	0.472	0.637	0.27
PCA2	-0.044	0.018	2.453	0.014	0.90	-0.024	0.017	1.389	0.165	0.46
TEB	-0.004	0.020	0.177	0.859	0.25	0.033	0.015	2.155	0.031	0.77
	Africa Shannon index_fam									
(Intercept)	5.186	0.031	168.170	0.000	NA	5.169	0.061	85.173	0.000	NA
C:N ratio	-0.019	0.038	0.492	0.623	0.28	-0.048	0.045	1.063	0.288	0.37
CWD	0.063	0.028	2.258	0.024	0.83	0.101	0.031	3.278	0.001	1.00
Shannon index_fam	-0.028	0.024	1.137	0.256	0.39	-0.040	0.023	1.781	0.075	0.63
MAP	0.066	0.047	1.390	0.165	0.52	-0.023	0.047	0.494	0.621	0.26
MAT	-0.030	0.026	1.167	0.243	0.40	0.031	0.028	1.101	0.271	0.37
PCA1	0.030	0.030	0.977	0.328	0.36	0.046	0.036	1.282	0.200	0.44
PCA2	-0.002	0.040	0.058	0.954	0.25	-0.026	0.045	0.586	0.558	0.27
TEB	-0.068	0.047	1.436	0.151	0.54	-0.010	0.046	0.208	0.835	0.24
	Asia Shannon index_fam									
(Intercept)	5.093	0.064	77.150	0.000	NA	5.219	0.144	36.124	0.000	NA
C:N ratio	0.014	0.024	0.578	0.563	0.12	-0.010	0.021	0.476	0.634	0.08
Shannon index_fam	0.009	0.047	0.187	0.852	0.09	0.039	0.035	1.128	0.259	0.15
MAP	0.175	0.041	4.121	0.000	1.00	0.080	0.036	2.227	0.026	0.47
MAT	-0.110	0.055	1.942	0.052	0.60	-0.102	0.041	2.485	0.013	0.70
PCA1	-0.019	0.069	0.272	0.785	0.10	-0.106	0.081	1.301	0.193	0.22
PCA2	0.010	0.163	0.058	0.954	0.10	-0.350	0.252	1.389	0.165	0.26
TEB	0.189	0.246	0.745	0.456	0.14	0.552	0.341	1.618	0.106	0.38
	South America Simpson index_sp									
(Intercept)	4.930	0.027	179.125	0.000	NA	4.839	0.113	42.939	0.000	NA
C:N ratio	0.051	0.024	2.068	0.039	0.78	0.007	0.018	0.409	0.683	0.26
CWD	0.162	0.029	5.456	0.000	1.00	0.102	0.034	3.015	0.003	0.98

Simpson index_sp	-0.017	0.027	0.632	0.528	0.29	0.016	0.020	0.800	0.424	0.30
MAP	-0.018	0.034	0.532	0.595	0.26	0.006	0.034	0.179	0.858	0.29
MAT	0.029	0.025	1.150	0.250	0.39	-0.009	0.021	0.422	0.673	0.25
PCA1	0.088	0.024	3.598	0.000	1.00	0.003	0.020	0.135	0.892	0.24
PCA2	-0.040	0.019	2.120	0.034	0.80	-0.025	0.017	1.444	0.149	0.48
TEB	-0.008	0.021	0.392	0.695	0.26	0.033	0.015	2.189	0.029	0.79
	Africa Simpson index_sp									
(Intercept)	5.191	0.032	160.645	0.000	NA	5.180	0.060	86.548	0.000	NA
C:N ratio	-0.017	0.038	0.450	0.653	0.28	-0.037	0.044	0.847	0.397	0.32
CWD	0.062	0.028	2.227	0.026	0.82	0.099	0.031	3.203	0.001	1.00
Simpson index_sp	0.007	0.051	0.139	0.890	0.24	-0.033	0.048	0.698	0.485	0.29
MAP	0.064	0.047	1.352	0.177	0.51	-0.031	0.046	0.667	0.505	0.28
MAT	-0.031	0.026	1.191	0.234	0.41	0.034	0.028	1.197	0.231	0.39
PCA1	0.029	0.030	0.943	0.346	0.35	0.035	0.035	1.021	0.307	0.36
PCA2	-0.007	0.039	0.184	0.854	0.25	-0.037	0.043	0.853	0.394	0.31
TEB	-0.069	0.047	1.471	0.141	0.55	-0.012	0.046	0.252	0.801	0.25
	Asia Simpson index_sp									
(Intercept)	5.041	0.060	81.465	0.000	NA	5.180	0.124	41.909	0.000	NA
C:N ratio	0.009	0.024	0.378	0.705	0.02	-0.012	0.020	0.576	0.565	0.05
Simpson index_sp	0.099	0.034	2.812	0.005	0.92	0.079	0.030	2.620	0.009	0.71
MAP	0.181	0.038	4.594	0.000	1.00	0.084	0.032	2.591	0.010	0.56
MAT	-0.136	0.050	2.610	0.009	0.78	-0.117	0.040	2.924	0.003	0.84
PCA1	-0.071	0.063	1.095	0.273	0.03	-0.103	0.072	1.431	0.152	0.15
PCA2	0.096	0.147	0.635	0.526	0.02	-0.311	0.279	1.117	0.264	0.10
TEB	0.222	0.225	0.953	0.341	0.03	0.482	0.329	1.465	0.143	0.19
	South America Simpson index_gen									
(Intercept)	4.936	0.027	178.797	0.000	NA	4.841	0.112	43.246	0.000	NA
C:N ratio	0.051	0.024	2.097	0.036	0.79	0.007	0.018	0.386	0.700	0.25
CWD	0.168	0.029	5.802	0.000	1.00	0.102	0.034	3.011	0.003	0.98
Simpson index_gen	-0.045	0.024	1.857	0.063	0.67	-0.013	0.018	0.736	0.462	0.30
MAP	-0.014	0.034	0.397	0.692	0.25	0.008	0.034	0.236	0.813	0.29
MAT	0.023	0.025	0.897	0.370	0.34	-0.010	0.022	0.486	0.627	0.26
PCA1	0.081	0.025	3.249	0.001	1.00	0.000	0.020	0.019	0.985	0.24
PCA2	-0.039	0.018	2.105	0.035	0.80	-0.025	0.017	1.454	0.146	0.48
TEB	-0.006	0.020	0.280	0.780	0.25	0.033	0.015	2.176	0.030	0.78
	Africa Simpson index_gen									
(Intercept)	5.188	0.030	170.405	0.000	NA	5.176	0.059	87.222	0.000	NA
C:N ratio	-0.020	0.038	0.512	0.609	0.29	-0.042	0.044	0.956	0.339	0.35
CWD	0.063	0.028	2.240	0.025	0.82	0.100	0.031	3.224	0.001	1.00
Simpson index_gen	-0.019	0.025	0.733	0.464	0.30	-0.035	0.024	1.484	0.138	0.50
MAP	0.064	0.047	1.352	0.176	0.50	-0.032	0.046	0.687	0.492	0.28
MAT	-0.031	0.026	1.186	0.236	0.41	0.034	0.028	1.205	0.228	0.39
PCA1	0.029	0.030	0.953	0.341	0.35	0.040	0.035	1.144	0.252	0.39
PCA2	-0.005	0.039	0.123	0.902	0.25	-0.033	0.043	0.767	0.443	0.30
TEB	-0.070	0.047	1.471	0.141	0.55	-0.013	0.046	0.285	0.776	0.24
	Asia Simpson index_gen									
(Intercept)	5.093	0.062	79.113	0.000	NA	5.223	0.144	36.344	0.000	NA
C:N ratio	0.015	0.024	0.598	0.550	0.11	-0.009	0.020	0.444	0.657	0.08
Simpson index_gen	0.046	0.052	0.866	0.387	0.15	0.037	0.040	0.925	0.355	0.12

MAP	0.175	0.041	4.101	0.000	1.00	0.081	0.036	2.261	0.024	0.49
MAT	-0.111	0.055	1.960	0.050	0.61	-0.103	0.041	2.534	0.011	0.71
PCA1	-0.021	0.069	0.290	0.772	0.10	-0.107	0.081	1.316	0.188	0.23
PCA2	0.022	0.150	0.141	0.888	0.08	-0.352	0.251	1.402	0.161	0.27
TEB	0.180	0.235	0.741	0.459	0.12	0.561	0.341	1.646	0.100	0.38
	South America Simpson index_fam									
(Intercept)	4.940	0.027	184.100	0.000	NA	4.842	0.111	43.608	0.000	NA
C:N ratio	0.034	0.025	1.386	0.166	0.48	0.006	0.018	0.323	0.747	0.25
CWD	0.166	0.027	6.141	0.000	1.00	0.101	0.034	2.965	0.003	0.98
Simpson index_fam	-0.085	0.028	3.036	0.002	1.00	-0.022	0.021	1.072	0.284	0.37
MAP	-0.011	0.033	0.324	0.746	0.25	0.009	0.034	0.266	0.790	0.29
MAT	0.008	0.025	0.318	0.751	0.25	-0.013	0.022	0.586	0.558	0.27
PCA1	0.064	0.024	2.608	0.009	0.94	-0.001	0.020	0.057	0.955	0.24
PCA2	-0.044	0.018	2.451	0.014	0.90	-0.025	0.017	1.429	0.153	0.47
TEB	-0.003	0.020	0.147	0.883	0.25	0.033	0.015	2.174	0.030	0.78
	Africa Simpson index_fam									
(Intercept)	5.189	0.030	171.774	0.000	NA	5.174	0.060	86.253	0.000	NA
C:N ratio	-0.018	0.038	0.472	0.637	0.28	-0.047	0.045	1.024	0.306	0.36
CWD	0.062	0.028	2.233	0.026	0.82	0.100	0.031	3.248	0.001	1.00
Simpson index_fam	-0.017	0.022	0.750	0.453	0.30	-0.034	0.021	1.618	0.106	0.56
MAP	0.065	0.047	1.375	0.169	0.51	-0.026	0.047	0.549	0.583	0.26
MAT	-0.031	0.026	1.190	0.234	0.41	0.031	0.028	1.076	0.282	0.36
PCA1	0.030	0.030	0.974	0.330	0.36	0.047	0.037	1.269	0.204	0.44
PCA2	-0.005	0.039	0.117	0.907	0.25	-0.029	0.044	0.660	0.510	0.28
TEB	-0.069	0.047	1.467	0.142	0.55	-0.011	0.046	0.244	0.808	0.24
	Asia Simpson index_fam									
(Intercept)	5.094	0.064	77.731	0.000	NA	5.221	0.144	36.161	0.000	NA
C:N ratio	0.014	0.024	0.578	0.563	0.12	-0.010	0.021	0.481	0.631	0.08
Simpson index_fam	-0.004	0.039	0.086	0.931	0.09	0.025	0.030	0.830	0.407	0.13
MAP	0.176	0.041	4.121	0.000	1.00	0.081	0.036	2.239	0.025	0.48
MAT	-0.110	0.055	1.945	0.052	0.60	-0.102	0.041	2.475	0.013	0.69
PCA1	-0.019	0.069	0.272	0.785	0.10	-0.106	0.081	1.308	0.191	0.23
PCA2	0.010	0.163	0.058	0.954	0.10	-0.352	0.251	1.401	0.161	0.27
TEB	0.189	0.246	0.745	0.456	0.14	0.556	0.341	1.628	0.103	0.39

Supplementary Table 6. Averaged simultaneous autoregressive model coefficients for ln(carbon) in 1 ha plots as a function of the community weighted mean of wood density (WD CWM), species richness, climate and soil variables.

Variable	β	SE	Z	P	$\sum\omega_i$
		South America			
Intercept	5.64	0.05	112.06	<0.001	NA
MAT	-0.10	0.03	3.86	<0.001	1
CWD	0.10	0.04	2.67	0.008	0.97
MAP	0.08	0.03	2.61	0.009	0.87
WD CWM	0.15	0.02	6.55	<0.001	0.45
Species richness	0.02	0.02	0.64	0.520	0.43
TEB	0.02	0.02	1.23	0.220	0.4
PCA1	0.01	0.02	0.36	0.721	0.29
C:N	0.00	0.02	0.07	0.941	0.25
PCA2	0.01	0.02	0.40	0.693	0.24
		Africa			
Intercept	5.90	0.06	101.31	<0.001	NA
WD CWM	0.18	0.02	7.61	<0.001	1
MAT	-0.03	0.03	1.37	0.172	1
CWD	0.08	0.03	2.79	0.005	0.96
TEB	0.07	0.03	2.41	0.016	0.92
MAP	-0.05	0.04	1.21	0.228	0.42
Species richness	0.06	0.05	1.29	0.198	0.28
PCA1	-0.02	0.03	0.72	0.469	0.25
PCA2	0.01	0.03	0.20	0.839	0.25
C:N	0.00	0.04	0.08	0.935	0.23
		Asia			
Intercept	5.98	0.11	52.26	<0.001	NA
MAP	0.11	0.03	3.35	0.001	0.99
MAT	-0.13	0.04	3.06	0.002	0.96
Species richness	0.11	0.04	2.50	0.012	0.79
WD CWM	0.17	0.07	2.24	0.025	0.65
C:N	-0.03	0.04	0.79	0.431	0.23
TEB	-0.04	0.07	0.66	0.510	0.2
PCA1	-0.03	0.05	0.59	0.554	0.2
PCA2	-0.03	0.07	0.38	0.707	0.18

Supplementary Table 7. Relationship between diversity and carbon in 0.04ha subplots within 1ha forest inventory plots. For each diversity metric, we constructed mixed effects models of ln (carbon) as a function of ln (diversity metric) and ln (stem density) with plot identify as a random effect. Coefficients were assumed to vary between plots, with SD showing the estimated standard deviation of this variation. The effect of doubling a diversity metric on carbon storage was calculated as (2^{β} -1) x 100. ^{0}D is species richness, ^{1}D is Shannon diversity and ^{2}D is Simpson diversity (see SI methods).

Diversity metric	β ± SE	SD	Effect of doubling (%)
^{0}D (species level)	0.096 ± 0.048	0.440	6.9
^{0}D (genus level)	0.110 ± 0.046	0.432	7.9
^{0}D (family level)	0.010 ± 0.039	0.338	0.7
^{1}D (species level)	0.053 ± 0.035	0.324	3.7
^{1}D (genus level)	0.061 ± 0.035	0.331	4.3
^{1}D (family level)	-0.007 ± 0.033	0.300	-0.5
^{2}D (species level)	0.026 ± 0.028	0.262	1.8
^{2}D (genus level)	0.034 ± 0.028	0.267	2.4
^{2}D (family level)	-0.020 ± 0.028	0.260	-1.4
^{0}D (species level) / 10 stems	0.133 ± 0.069	0.585	9.7
^{0}D (genus level) / 10 stems	0.133 ± 0.064	0.572	9.7
^{0}D (family level) / 10 stems	0.017 ± 0.011	0.046	1.2

References

1. Lopez-Gonzalez, G., Lewis, S. L., Burkitt, M. & Phillips, O. L. ForestPlots.net: a web application and research tool to manage and analyse tropical forest plot data. *Journal of Vegetation Science* **22**, 610-613, doi:10.1111/j.1654-1103.2011.01312.x (2011).
2. Phillips, O. L., Baker, T. R., Feldpausch, T. R. & Brienen, R. J. W. RAINFOR field manual for plot establishment and remeasurement. (University of Leeds, Leeds, 2009).
3. IUSS Working GROUP WRB. World reference base for soil resources 2006. (Rome, 2006).
4. Quesada, C. A. *et al.* Variations in chemical and physical properties of Amazon forest soils in relation to their genesis. *Biogeosciences* **7**, 1515-1541, doi:10.5194/bg-7-1515-2010 (2010).
5. Lewis, S. L. *et al.* Above-ground biomass and structure of 260 African tropical forests. *Philosophical Transactions of the Royal Society B: Biological Sciences* **368**, doi:10.1098/rstb.2012.0295 (2013).
6. FAO/ IIASA/ ISRIC/ ISSCAS/ JRC. Harmonized World Soil Database (version 1.2). (Rome, Italy and Laxenburg, Austria, 2012).
7. Batjes, J. & through ISRIC/WDC-Soils. (ed University of Ghent) (Ghent, Belgium, 2007).
8. Hijmans, R. J., Cameron, S. E., Parra, J. L., Jones, P. G. & Jarvis, A. Very high resolution interpolated climate surfaces for global land areas. *International Journal of Climatology* **25**, 1965-1978, doi:10.1002/joc.1276 (2005).
9. Harris, I., Jones, P. D., Osborn, T. J. & Lister, D. H. Updated high-resolution grids of monthly climatic observations ‐ the CRU TS3.10 Dataset. *International Journal of Climatology* **34**, 623-642, doi:10.1002/joc.3711 (2014).
10. Vásquez, R. *et al. Flórula de las reservas biológicas de Iquitos, Perú: Allpahuayo-Mishana, Explornapo Camp, Explorama Lodge*. (Missouri Botanical Garden, 1997).
11. Hill, M. O. Diversity and Evenness: A Unifying Notation and Its Consequences. *Ecology* **54**, 427-432, doi:10.2307/1934352 (1973).
12. Millar, R. B., Anderson, M. J. & Tolimieri, N. Much ado about nothings: using zero similarity points in distance-decay curves. *Ecology* **92**, 1717-1722, doi:10.1890/11-0029.1 (2011).
13. Slik, J. W. F. *et al.* An estimate of the number of tropical tree species. *Proceedings of the National Academy of Sciences*, doi:10.1073/pnas.1423147112 (2015).
14. Beck, J., Holloway, J. D. & Schwanghart, W. Undersampling and the measurement of beta diversity. *Methods in Ecology and Evolution* **4**, 370-382, doi:10.1111/2041-210x.12023 (2013).
15. Cayuela, L., Granzow-de la Cerda, Í., Albuquerque, F. S. & Golicher, D. J. taxonstand: An r package for species names standardisation in vegetation databases. *Methods in Ecology and Evolution* **3**, 1078-1083, doi:10.1111/j.2041-210X.2012.00232.x (2012).
16. Hothorn, T., Bretz, F. & Westfall, P. Simultaneous Inference in General Parametric Models. *Biometrical Journal* **50**, 346-363, doi:10.1002/bimj.200810425 (2008).
17. pwr: Basic functions for power analysis. R package version 1.1.1. htto://CRAN.R-project.org/package=pwr (2012).
18. Gilpin, A. R. Table for Conversion of Kendall'S Tau to Spearman'S Rho Within the Context of Measures of Magnitude of Effect for Meta-Analysis. *Educational and Psychological Measurement* **53**, 87-92, doi:10.1177/0013164493053001007 (1993).
19. Barton, K. (2015).
20. ncf: spatial nonparametric covariance functions. R package version 1.1-5. http://CRAN.R-project.org/package=ncf (2013).
21. spdep: Spatial dependence: weighting schemes, statsitics and models. R package version 0.5-77. http://CRAN.R-project.org/package=spdep (2014).
22. F. Dormann, C. *et al.* Methods to account for spatial autocorrelation in the analysis of species distributional data: a review. *Ecography* **30**, 609-628, doi:10.1111/j.2007.0906-7590.05171.x (2007).

23 Kissling, W. D. & Carl, G. Spatial autocorrelation and the selection of simultaneous autoregressive models. *Global Ecology and Biogeography* **17**, 59-71, doi:10.1111/j.1466-8238.2007.00334.x (2008).

24 Mauricio Bini, L. *et al.* Coefficient shifts in geographical ecology: an empirical evaluation of spatial and non-spatial regression. *Ecography* **32**, 193-204, doi:10.1111/j.1600-0587.2009.05717.x (2009).

25 Chisholm, R. A. *et al.* Scale-dependent relationships between tree species richness and ecosystem function in forests. *Journal of Ecology* **101**, 1214-1224, doi:10.1111/1365-2745.12132 (2013).

26 lme4: Linear mixed-effects models using Eigen and S4 v. R package version 1.1-7 (2014).

27 Loreau, M. & Hector, A. Partitioning selection and complementarity in biodiversity experiments. *Nature* **412**, 72-76, doi:10.1038/35083573 (2001).

28 Jucker, T., Bouriaud, O. & Coomes, D. A. Crown plasticity enables trees to optimize canopy packing in mixed-species forests. *Functional Ecology* **29**, 1078-1086, doi:10.1111/1365-2435.12428 (2015).

29 Cavanaugh, K. C. *et al.* Carbon storage in tropical forests correlates with taxonomic diversity and functional dominance on a global scale. *Global Ecology and Biogeography* **23**, 563-573, doi:10.1111/geb.12143 (2014).

30 Finegan, B. *et al.* Does functional trait diversity predict above-ground biomass and productivity of tropical forests? Testing three alternative hypotheses. *Journal of Ecology* **103**, 191-201, doi:10.1111/1365-2745.12346 (2015).

31 Zanne, A. E. *et al.* (Dryad Data Repository, 2009).

32 Chave, J. *et al.* Towards a worldwide wood economics spectrum. *Ecology Letters* **12**, 351-366, doi:10.1111/j.1461-0248.2009.01285.x (2009).

33 Lewis, S. L. *et al.* Increasing carbon storage in intact African tropical forests. *Nature* **457**, 1003-1006, doi:http://www.nature.com/nature/journal/v457/n7232/suppinfo/nature07771_S1.html (2009).

34 Brienen, R. J. W. *et al.* Long-term decline of the Amazon carbon sink. *Nature* **519**, 344-348, doi:10.1038/nature14283

http://www.nature.com/nature/journal/v519/n7543/abs/nature14283.html#supplementary-information (2015).

35 Chave, J. *et al.* Assessing Evidence for a Pervasive Alteration in Tropical Tree Communities. *PLoS Biol* **6**, e45, doi:10.1371/journal.pbio.0060045 (2008).

36 Baraloto, C. *et al.* Functional trait variation and sampling strategies in species-rich plant communities. *Functional Ecology* **24**, 208-216, doi:10.1111/j.1365-2435.2009.01600.x (2010).

37 Wright, S. J. *et al.* Functional traits and the growth-mortality trade-off in tropical trees. *Ecology* **91**, 3664-3674, doi:10.1890/09-2335.1 (2010).

38 Van Gelder, H. A., Poorter, L. & Sterck, F. J. Wood mechanics, allometry, and life-history variation in a tropical rain forest tree community. *New Phytologist* **171**, 367-378, doi:10.1111/j.1469-8137.2006.01757.x (2006).

39 Falster, D. S. Sapling strength and safety: the importance of wood density in tropical forests. *New Phytologist* **171**, 237-239, doi:10.1111/j.1469-8137.2006.01809.x (2006).

40 Fyllas, N. M., Quesada, C. A. & Lloyd, J. Deriving Plant Functional Types for Amazonian forests for use in vegetation dynamics models. *Perspectives in Plant Ecology, Evolution and Systematics* **14**, 97-110, doi:http://dx.doi.org/10.1016/j.ppees.2011.11.001 (2012).

41 Aubry-Kientz, M., Hérault, B., Ayotte-Trépanier, C., Baraloto, C. & Rossi, V. Toward Trait-Based Mortality Models for Tropical Forests. *PLoS ONE* **8**, e63678, doi:10.1371/journal.pone.0063678 (2013).

42 Galbraith, D. *et al.* Residence times of woody biomass in tropical forests. *Plant Ecology & Diversity* **6**, 139-157, doi:10.1080/17550874.2013.770578 (2013).

43 Petchey, O. L. & Gaston, K. J. Functional diversity: back to basics and looking forward. *Ecology Letters* **9**, 741-758, doi:10.1111/j.1461-0248.2006.00924.x (2006).

44 Fauset, S. *et al.* Hyperdominance in Amazonian forest carbon cycling. *Nat Commun* **6**, doi:10.1038/ncomms7857 (2015).

45 Slik, J. W. F. *et al.* Large trees drive forest aboveground biomass variation in moist lowland forests across the tropics. *Global Ecology and Biogeography* **22**, 1261-1271, doi:10.1111/geb.12092 (2013).

46 Grime, J. P. Benefits of plant diversity to ecosystems: immediate, filter and founder effects. *Journal of Ecology* **86**, 902-910, doi:10.1046/j.1365-2745.1998.00306.x (1998).

47 Kraft, N. J. B. *et al.* Disentangling the Drivers of β Diversity Along Latitudinal and Elevational Gradients. *Science* **333**, 1755-1758, doi:10.1126/science.1208584 (2011).
